# Convergent and divergent brain–cognition development in early adolescence

**DOI:** 10.1038/s41467-026-73668-y

**Published:** 2026-05-26

**Authors:** Yapei Xie, Shaoshi Zhang, Csaba Orban, Leon Qi Rong Ooi, Ru Kong, Dorothea L. Floris, Xi-Nian Zuo, Elvisha Dhamala, Avram J. Holmes, Lucina Q. Uddin, Thomas E. Nichols, Adriana Di Martino, B. T. Thomas Yeo

**Affiliations:** 1 Centre for Sleep and Cognition & Centre for Translational MR Research, Yong Loo Lin School of Medicine, National University of Singapore, Singapore, Singapore; 2 Department of Medicine, Healthy Longevity Translational Research Programme, Human Potential Translational Research Programme & Institute for Digital Medicine (WisDM), Yong Loo Lin School of Medicine, National University of Singapore, Singapore, Singapore; 3 Department of Electrical and Computer Engineering, National University of Singapore, Singapore, Singapore; 4 N.1 Institute for Health, National University of Singapore, Singapore, Singapore; 5 Integrative Sciences and Engineering Programme (ISEP), National University of Singapore, Singapore, Singapore; 6 Methods of Plasticity Research, Department of Psychology, University of Zurich, Zurich, Switzerland; 7 Donders Institute, Centre for Cognitive Neuroimaging, Radboud University, Nijmegen, Netherlands; 8 State Key Laboratory of Cognitive Neuroscience and Learning, Beijing Normal University, Beijing, China; 9 National Basic Science Data Center, Beijing, China; 10 Developmental Population Neuroscience Research Center, IDG/McGovern Institute for Brain Research, Beijing Normal University, Beijing, China; 11 Institute of Behavioral Science, Feinstein Institutes for Medical Research, Manhasset, NY USA; 12 Division of Psychiatry Research, Zucker Hillside Hospital, Glen Oaks, NY USA; 13 Donald and Barbara Zucker School of Medicine at Hofstra/Northwell, Uniondale, NY USA; 14 Department of Psychiatry, Brain Health Institute, Rutgers University, Piscataway, NJ USA; 15 Department of Psychiatry and Biobehavioral Sciences, University of California Los Angeles, Los Angeles, CA USA; 16 Big Data Institute, Li Ka Shing Centre for Health Information and Discovery, Nuffield Department of Population Health, University of Oxford, Oxford, UK; 17 Autism Center, Child Mind Institute, New York, NY USA; 18 Martinos Center for Biomedical Imaging, Massachusetts General Hospital, Charlestown, MA USA

**Keywords:** Cognitive neuroscience, Computational neuroscience

## Abstract

How functional brain networks and cognition co-evolve during adolescent development remains poorly understood. Using baseline and Year 2 data from 2949 individuals in the Adolescent Brain Cognitive Development Study, we trained kernel ridge regression models to predict cognitive ability from resting-state functional connectivity. We find that baseline functional connectivity more strongly predicts future cognitive ability than baseline cognitive ability. Models trained on baseline functional connectivity to predict baseline cognition generalize better to Year 2 functional connectivity and cognition, suggesting that brain–cognition relationships strengthen over time. Intriguingly, baseline functional connectivity outperforms longitudinal functional connectivity change in predicting future cognitive ability. While longitudinal functional connectivity change is less reliable than baseline functional connectivity – intraclass correlation coefficient 0.24 vs. 0.56 – shortening scan duration to reduce reliability of baseline functional connectivity does not eliminate the predictive gap. Furthermore, neither baseline functional connectivity nor functional connectivity change meaningfully predicts longitudinal change in cognitive ability. We also identify converging and diverging predictive network features across cross-sectional and longitudinal brain-cognition models – a multivariate twist on Simpson’s paradox – with clear sex-specific patterns. Overall, in early adolescence, stable individual differences in brain functional network organization play a more critical role than dynamic changes in shaping future cognitive outcomes.

## Abstract (web summary)

As children enter adolescence, thinking becomes more abstract, coinciding with large-scale changes in brain network organization. Here, the authors show that stable brain network organization plays a more critical role than dynamic changes in shaping cognitive outcomes.

## Introduction

A major goal in cognitive neuroscience is to understand how individual differences in brain network development relate to variability in cognitive development. This is particularly salient during the transition from childhood to adolescence, a period marked by a shift from concrete to more abstract and logical thinking, which supports the growing ability to manage complex tasks and navigate increasingly demanding environments^1–4^. Here, we leverage longitudinal resting-state fMRI and cognitive data from 2949 children (ages 8.9–13.5) in the Adolescent Brain Cognitive Development (ABCD) Study at baseline and Year 2 to examine how stable and changing features of brain network organization predict cognitive development during this critical period.

Resting-state fMRI (rs-fMRI) is a powerful tool for examining functional brain network organization^5–8^. Contemporary network neuroscience theories propose that individual differences in cognitive ability arise from corresponding differences in brain network architecture^9^. Consistent with this framework, previous studies have developed cross-sectional brain–cognition models that can predict individual-level cognitive performance in children, adolescents, and young adults based on resting-state functional connectivity (FC)^10–18^. While valuable, these cross-sectional models reflect a single snapshot of brain–cognition relationships, neglecting dynamic changes that can only be revealed in longitudinal designs^19–22^.

**Fig. 1:**
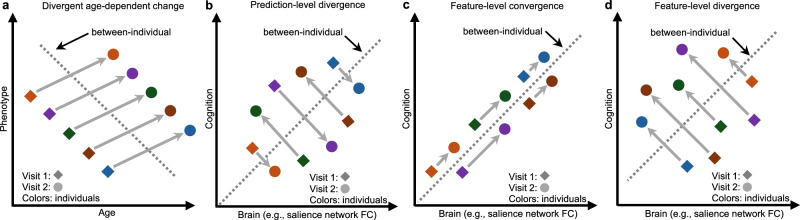
Illustration of how cross-sectional and longitudinal estimates can converge or diverge. Colours represent individual participants; diamond and circular symbols indicate baseline and Year 2, respectively. **a** Classical (Simpson’s paradox) illustration, where within-individual changes (arrows) diverge from between-individual differences (dashed line). **b** Predictive power divergence between cross-sectional and longitudinal predictive models. In this example, salience network FC yields a strong cross-sectional prediction of cognitive ability (dotted line). However, longitudinal within-individual change in salience network FC is not predictive of cognitive ability at visit 2, as well as longitudinal cognitive change (grey arrows). **c** Feature-level convergence between cross-sectional and longitudinal models. For example, greater salience network FC predicts better cognitive ability among children cross-sectionally (dashed line). Assuming a causal relationship, individuals with larger increases in salience network FC should enjoy greater cognitive gains longitudinally (arrows). **d** Feature-level divergence between cross-sectional and longitudinal models. Similar to panel (**c**), greater salience network FC predicts better cognitive ability among children cross-sectionally (dashed line). However, individuals with larger reductions in salience network FC enjoy greater cognitive gains longitudinally (arrows). Fig. S1 illustrates other possible divergences between cross-sectional and longitudinal estimates of the brain–cognition relationship. We hypothesize that convergence and divergence vary across brain networks.

Conceptually, cross-sectional analyses reveal between-individual traits, which might differ from dynamic within-individual changes captured by longitudinal designs^23,24^. Figure 1a illustrates the classic example of how cross-sectional (between-individual) and longitudinal (within-individual) estimates can diverge for a single phenotype, a phenomenon known as Simpson’s paradox^25–27^. When extended to predictive models of brain and cognition, the situation becomes more complex. For example, salience network FC may robustly predict cognitive ability cross-sectionally across individuals (Fig. 1b). However, longitudinal change in salience network FC might not be predictive of future cognitive ability or longitudinal change in cognitive ability (Fig. 1b).

Beyond predictive power, network features supporting prediction might also differ between cross-sectional and longitudinal models. For example, a stronger salience network FC predicted better cognitive ability at the individual level^11^. If this relationship reflects a causal mechanism, one would expect that children with greater increases in salience network FC over time would also show greater cognitive improvement (Fig. 1c). However, cross-sectional and longitudinal models may not always align: it is also possible that children with larger reductions in salience network FC exhibit the most cognitive gains (Fig. 1d). To put it in another way, suppose Luca is a cognitively advanced child whose brain connectivity patterns differ from his peers. As his peers grow older and become more cognitively capable, do their brains become more like Luca’s? If so, this would reflect feature-level convergence between cross-sectional and longitudinal estimates (Fig. 1c). If not, this would indicate feature-level divergence (Fig. 1d).

In this study, we examined how FC and cognition co-evolve during early adolescence using baseline and Year 2 data from 2949 participants in the ABCD Study^28–30^. We first quantified stability and longitudinal change in cognition and FC, observing that FC changes mirrored the sensorimotor–association axis. We then trained kernel ridge regression models to predict cognition from FC. Baseline FC predicted future cognition better than baseline cognition, suggesting that FC–cognition relationships strengthened with development. Baseline FC also outperformed longitudinal FC change at predicting future cognition. Although longitudinal FC change was substantially less reliable than baseline FC—the first such estimate, to our knowledge—matching their reliabilities by shortening baseline scan duration did not close the predictive gap. Neither baseline FC nor FC change meaningfully predicted cognitive change. Finally, contrasting cross-sectional and longitudinal models revealed both convergent and divergent predictive network features, uncovering a multivariate manifestation of Simpson’s paradox, with clear sex-specific differences. Together, these findings reveal how stable architecture and dynamic changes relate to adolescent cognition.

## Results

### Individual differences in longitudinal cognitive changes

We considered a sample of 2949 participants from the ABCD Study (releases 4 and 5), who had seven cognitive measures and rs-fMRI data that survived quality control at both baseline and Year 2 (Table 1). We also computed the first principal component (PC1) of the seven cognitive measures.

**Table 1:** Age, head motion, and cognitive measures at baseline, Year 2, and their longitudinal change

	Baseline	Year 2	Longitudinal change (raw/standardized)	
Participants(F/M)	2949 (1447/1502)	Same as baseline	N.A.	
Age (years)	9.93 (0.62)	11.94 (0.65)	2.01 (0.16)	N.A.
Mean FD (mm)	0.07 (0.04)	0.06 (0.03)	−0.01 (0.04)	−0.25 (1.03)
Std FD (mm)	0.12 (0.09)	0.09 (0.08)	−0.03 (0.10)	−0.28 (1.08)
RAVLT	69.93 (14.96)	69.59 (13.95)	−0.35 (13)	−0.02 (0.87)
LMT	0.61 (0.17)	0.75 (0.18)	0.14 (0.18)	0.82 (1.02)
PicVocab	85.54 (7.97)	89.73 (8.39)	4.19 (6.14)	0.53 (0.77)
Flanker	94.97 (8.33)	100.58 (7.13)	5.61 (8.45)	0.67 (1.01)
Pattern	89.06 (14.06)	104.15 (14.45)	15.09 (14.73)	1.07 (1.05)
Picture	104.15 (12.05)	110.16 (11.93)	6.01 (12.61)	0.5 (1.05)
Reading	91.72 (6.81)	95.42 (6.65)	3.70 (4.74)	0.54 (0.7)
PC1	0.43 (1.55)	1.86 (1.55)	1.43 (0.95)	0.92 (0.61)

Data are shown for 2949 participants (1447 female/1502 male). Values are presented as mean (standard deviation). Raw longitudinal change was computed as Year 2 minus baseline. Standardized longitudinal change was obtained by dividing the raw change by the baseline standard deviation of each measure, enabling comparison of change magnitudes across cognitive measures. Mean FD, mean framewise displacement across volumes; Std FD, standard deviation of framewise displacement across volumes; RAVLT, Rey Auditory Verbal Learning Test (verbal memory); LMT, Little Man Task (spatial reasoning); PicVocab, Picture Vocabulary Task (vocabulary); Flanker, Flanker Task (executive function); Pattern, Pattern Comparison Processing Speed Test (processing speed); Picture, Picture Sequence Memory Test (episodic memory); Reading, Oral Reading Recognition Task (reading ability); PC1, the first principal component of the above seven cognitive measures. Consistent with previous work^84^, we used uncorrected standard scores for each NIH Toolbox task, total correct scores for the RAVLT, and per cent correct scores for the LMT. For all cognitive measures, a greater value indicates better performance. See a more detailed explanation of cognitive measures in Methods.

**Fig. 2:**
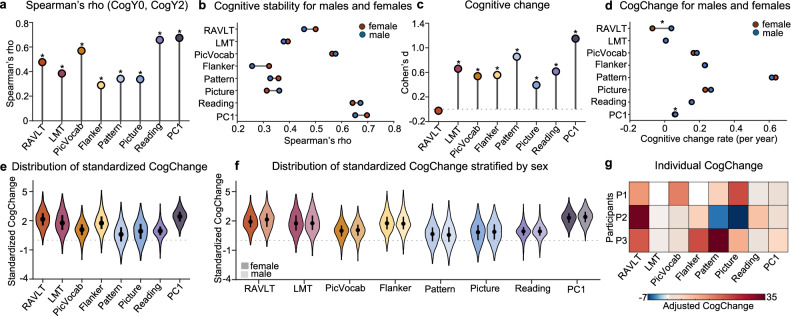
Individual differences in longitudinal cognitive change in early adolescence. **a** Spearman’s correlation between baseline and Year 2 cognitive measures (i.e., cognitive stability). Each marker represents a cognitive measure. Positive correlations indicate that children with higher baseline cognition generally maintained their cognitive advantage over their peers at Year 2. **b** Spearman’s correlation between baseline and Year 2 cognitive measures (i.e., cognitive stability) stratified by sex. Each marker represents a cognitive measure, with horizontal lines connecting female and male estimates. Colours indicate sex (female: red; male: blue). **c** Longitudinal cognitive change at the group level estimated from a linear mixed-effects model. Values represent Cohen’s *d*. **d** Cognitive change rate for males and females estimated from a linear mixed effects model. Values represent the estimated annual rate of change. **e** Individual variability in standardized longitudinal cognitive change. For each individual and each cognitive measure, cognitive change was defined as the difference in scores between the two timepoints. Sex, baseline age, and age interval (between baseline and Year 2) were regressed out. The residual change scores were then standardized by the baseline standard deviation of each measure. Violin plots show the distribution of standardized cognitive change; centre dots denote the median, and vertical bars denote the interquartile range (25th–75th percentile). Colours distinguish different cognitive measures. **f** Individual variability in standardized longitudinal cognitive change stratified by sex. Visualization elements are as in (**e**). **g** Longitudinal changes in the eight cognitive measures for three participants. Colours denote adjusted cognitive change. All analyses were performed on *n* = 2949 participants (female/male:1447/1502), unless otherwise stated. Asterisks (*) indicate statistical significance (two-sided tests) after false discovery rate (FDR) correction^89^ (*q* < 0.05). RAVLT, Rey Auditory Verbal Learning Test (verbal memory); LMT, Little Man Task (spatial reasoning); PicVocab, Picture Vocabulary Task (vocabulary); Flanker, Flanker Task (executive function); Pattern, Pattern Comparison Processing Speed Test (processing speed); Picture, Picture Sequence Memory Test (episodic memory); Reading, Oral Reading Recognition Task (reading ability); PC1, the first principal component of the above seven cognitive measures.

Cognitive ability at baseline and Year 2 was positively correlated, suggesting that individuals with higher baseline cognition generally maintained their cognitive advantage over their peers at Year 2 (Fig. 2a). No significant sex differences were observed in cognitive stability (Figs. 2b and S2).

At the group level, cognitive performance improved from baseline to Year 2 across most measures (Fig. 2c), with the notable exception of the Rey Auditory Verbal Learning Test (RAVLT). A mixed-effects model revealed a significant sex-by-age interval interaction for RAVLT (*t* = −4.41, FDR *q* < 0.05; Table S1), with males showing improvement over time and females exhibiting a slight decline (Fig. 2d). Consequently, there was no improvement in RAVLT for the full sample (Fig. 2c). In addition, males demonstrated a slightly greater rate of longitudinal improvement than females in overall cognition (PC1; *t* = −2.40, FDR *q* < 0.05; Fig. 2d).

Beyond group-level changes, there was substantial individual variability in longitudinal cognitive change across most cognitive measures (Fig. 2e), and within each sex (Fig. 2f). Sex differences in the magnitude of individual variability were observed for RAVLT and Pattern Comparison Processing Speed Test, with males exhibiting greater variability than females for both measures (FDR *q* < 0.05).

To illustrate the inter-individual heterogeneity, Fig. 2g shows the longitudinal changes of the eight cognitive measures for three participants. Pearson’s correlations among the three cognitive change profiles were small (average *r* = 1.9 × 10⁻^4^), suggesting that the patterns of cognitive improvement were highly different across the three participants. For example, compared with their peers, participant 2 became substantially worse in the Picture Sequence Memory Task (“Picture”), but exhibited relatively strong improvement in RAVLT. On the other hand, participant 1 showed great improvements in both the Picture and RAVLT tasks.

### Individual differences in longitudinal FC changes

**Fig. 3:**
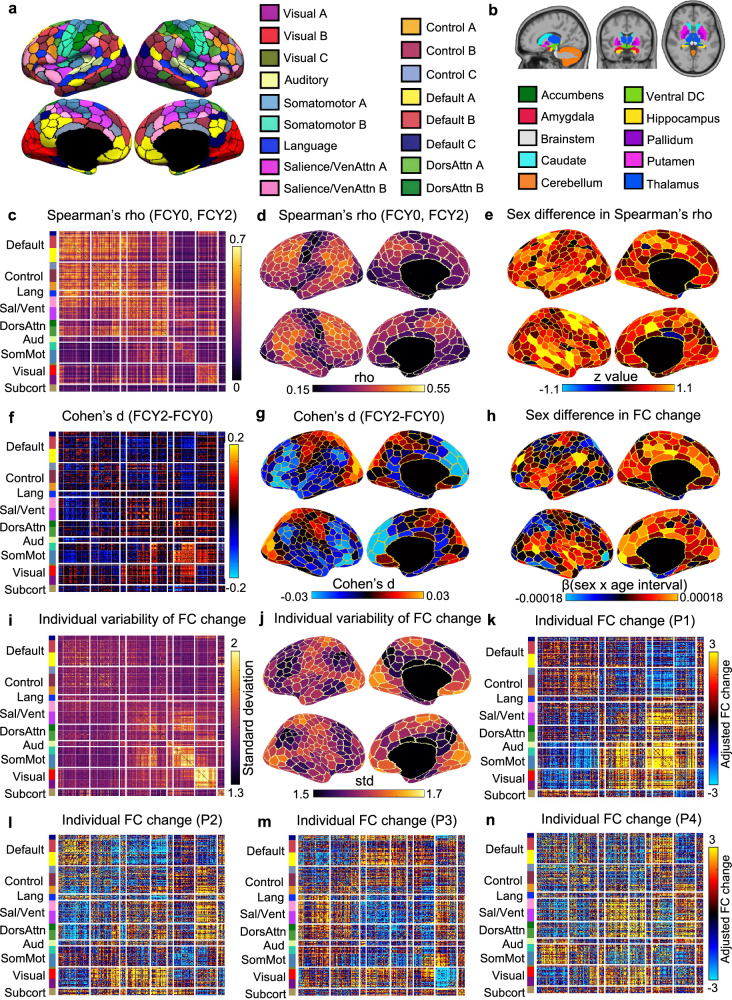
Individual differences in longitudinal FC change. **a** Cortical parcellation of 400 regions^31^, which is the homotopic variant of the Schaefer parcellation^95^. Parcel colours are assigned corresponding to 17 large-scale networks^96^. **b** 19 subcortical regions^32^. Colours denote different subcortical structures. 419 × 419 FC matrices were computed based on the 419 cortical and subcortical regions. **c** Spearman’s correlation (stability) between baseline (FCY0) and Year 2 FC (FCY2) for each FC edge. Positive correlations (rho; warmer colours) indicate that children exhibiting stronger functional brain connectivity at baseline continued to exhibit stronger functional brain connectivity than their peers at Year 2. 99.995% of entries were significant after FDR correction (*q* < 0.05). **d** Visualization of FC stability at the regional level, by averaging the rows of panel **c**. **e** Sex differences in FC stability at the regional level (unthresholded), shown as *z* values (female−male). **f** Longitudinal FC change at the group level based on a linear mixed-effects model. 54.96% of entries were significant after FDR correction (*q* < 0.05). Values are shown as Cohen’s *d*. **g** Visualization of longitudinal FC change at the regional level, by averaging the rows of panel **f**. **h** Sex differences in FC change at the regional level (unthresholded), quantified by the coefficient (*β*) of the sex × age interval term (female−male slope). **i** Individual variability in longitudinal FC change. FC change (*z* value) was computed for each FC edge^86^. Sex, baseline age, age interval (between baseline and Year 2), and head motion at two timepoints were regressed out. Standard deviation was then computed across individuals. **j** Visualization of individual variability in longitudinal FC change at the regional level, by averaging the rows of panel **i**. **k**–**n** Individual-level FC change for four participants. Colours indicate the direction of FC change (warmer, positive; cooler, negative). All statistical tests were two-sided unless otherwise stated.

Turning our attention to brain network organization, the rs-fMRI was used to compute a 419 × 419 FC matrix for each participant and each time point with the 400-region Yan parcellation^31^ (Fig. 3a) and 19 subcortical regions^32^ (Fig. 3b). Similar to cognition, baseline and Year 2 FC was positively correlated, suggesting that children exhibiting stronger functional brain connectivity at baseline continued to exhibit stronger functional brain connectivity than their peers at Year 2 (Fig. 3c).

By averaging the rows of Fig. 3c, clear regional variation was observed along the sensorimotor–association (S–A) axis^33,34^, with heteromodal association cortex being the most stable, while sensory-motor and visual systems were the least stable (Fig. 3d). Similar patterns were observed separately for males and females (Fig. S3a–d). Females exhibited higher FC stability than males across almost all brain regions (Fig. 3e), but these differences were small and few regions survived FDR correction (Fig. S3e–h).

Longitudinal FC change also mirrored the S–A axis: sensory-motor and visual systems showed the greatest longitudinal increase, while heteromodal association cortex exhibited the greatest longitudinal decrease (Fig. 3f and g). Similar patterns were observed separately for males and females (Fig. S3i–l), but females exhibited a statistically lower rate of FC decrease than males in the association cortex, such as the anterior insula and anterior cingulate cortex (Figs. 3h and S3m–p).

Beyond group-level changes, we also observed notable individual differences in FC change, especially for somatomotor and visual networks, as well as medial prefrontal cortex (Figs. 3i and j). A similar pattern was observed for males and females with no statistically significant sex difference (Fig. S3q–t).

To illustrate the inter-individual heterogeneity in FC change, Fig. 3k–n shows the longitudinal FC changes of four participants. Pearson’s correlations among the four FC change profiles were small (average *r* = −0.004), suggesting that the patterns of FC change were highly different across the four participants. For example, relative to their peers, participant 1 exhibited strong increases in FC within the somatomotor network, in contrast to participant 2, who exhibited strong decreases in somatomotor FC.

The substantial individual differences in cognition and FC change motivate the study of how the brain–cognition relationship evolves during the transition from childhood to adolescence.

### The relationship between FC and cognition strengthens with development

We next examined cross-sectional relationships between FC and cognition. We first used kernel ridge regression (KRR) to predict cross-sectional cognition from cross-sectional FC. This was achieved via a leave-3-site-cluster-out nested cross-validation procedure that was repeated 120 times for robustness (see the “Methods” section for details). Care was taken so that participants from the same site were not split between training and test sets, so prediction performance was always measured in out-of-sample sites not used to train the models.

The cross-validation procedure was repeated for all eight cognitive measures. To streamline the result representation, we focus on the first cognitive principal component (PC1) when a similar trend was observed across all eight cognitive measures. PC1 was chosen as the representative measure because PC1 provides a parsimonious summary of general cognitive performance and often yields the highest prediction accuracy. Results for the seven individual cognitive measures are reported in the supplement.

**Fig. 4:**
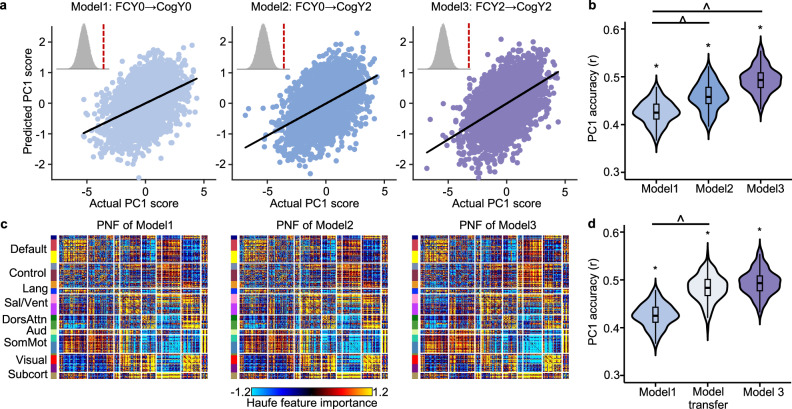
Enhanced FC–cognition relationships during development. **a** Correlation between the actual and predicted cognitive principal component (PC1) scores. Model 1 predicts baseline cognition using baseline FC (FCY0 → CogY0, light blue). Model 2 predicts Year 2 cognition using baseline FC (FCY0 → CogY2, blue). Model 3 predicts Year 2 cognition using Year 2 FC (FCY2 → CogY2, purple). Insets show null distributions based on 1000 permutations, with the red-dashed line corresponding to actual prediction accuracy. **b** Comparison of prediction accuracies across the three models. Each value in the violin plot represents the accuracy (*r*) for a single cross-validation fold. Inset box plots indicate the median (centre line), interquartile range (25th–75th percentile; box), and whiskers extending to 1.5× the interquartile range, with outliers not shown. Asterisks (*) denote above-chance prediction after multiple comparisons correction (FDR *q* < 0.05). Carets (^) denote statistically significant differences between models based on the corrected resampled *t*-test^88^ with FDR *q* < 0.05. **c** Predictive network feature (PNF) matrices for each model. PNFs were computed using the Haufe transformation^35^. Positive values (warmer colours) indicate that higher FC is associated with higher predicted cognitive scores, while negative values indicate associations with lower predicted cognitive scores. For visualization purposes, each predictive network feature matrix was normalized by dividing all values by the standard deviation of the entire matrix. **d** Models trained on baseline FC to predict baseline cognition improve in accuracy when applied to Year 2 FC and Year 2 cognition. Model 1 was used to predict Year 2 cognition from Year 2 FC, which we refer to as “model transfer” (grey violin). Other visualization elements are as in (**b**). Figures S4 and S8 repeat panels **b** and **d** for the other seven cognitive measures, respectively. FC, functional connectivity; PC1, the first principal component of the seven cognitive measures.

Consistent with previous work^11,12^, baseline FC predicted all eight cognitive measures at baseline (FDR < 0.05), with prediction accuracy of *r* = 0.43 for PC1 (Fig. 4a). Baseline FC also effectively predicted all eight cognitive measures at Year 2, with accuracy of *r* = 0.46 for PC1. We additionally found that Year 2 FC predicted all cognitive measures at Year 2, achieving an accuracy of *r* = 0.49 for PC1. Year 2 FC’s prediction of Year 2 PC1 was statistically better than baseline FC’s prediction of baseline PC1, and baseline FC predicted Year 2 cognition more accurately than it predicted PC1 at baseline (FDR < 0.05; Fig. 4b).

Figure S4 shows the comparisons for the other seven cognitive measures. Picture Vocabulary Task and Oral Reading Recognition Task exhibited comparatively high prediction accuracy, consistent with their strong contributions to the general cognitive ability component captured by PC1. All seven cognitive measures exhibited the same trend as PC1, in which the prediction of Year 2 cognition was better than the prediction of baseline cognition.

We further utilized the Haufe transformation^35^ to interpret each predictive model. Briefly, the covariance between each FC edge and the predicted cognition score was computed across participants. This analysis produced a 419 × 419 predictive network feature (PNF) matrix for each model and cognitive measure, where positive values indicate that higher FC were associated with higher predicted cognition scores. Previous studies have demonstrated that the Haufe transformation led to a more valid interpretation of predictive models^35^, which are highly reliable and generalizable across different predictive algorithms^11,36,37^.

Predictive network features were highly similar across all three models, with positive and negative contributions widely distributed across the entire connectome (Fig. 4c). For all three models, within-network FC contributed more towards prediction performance than between-network FC (Fig. S5). Intriguingly, the predictive network features of Model 3 (i.e., FCY2 → CogY2) exhibited a more pronounced contrast (Fig. 4c), with both stronger positive and stronger negative contributions compared to predictive network features of Model 1 (i.e., FCY0 → CogY0) and Model 2 (i.e., FCY0 → CogY2).

In sex-stratified analyses, the three predictive models (FCY0 → CogY0, FCY0 → CogY2, FCY2 → CogY2) were re-trained and re-evaluated separately for males and females. All cognitive measures were predicted better than chance, except that the Pattern Comparison Processing Speed Test was not predicted better than chance in males in the FCY0 → CogY0 model (Figs. S6 and S7). For both sexes, prediction accuracy also improved when predicting Year 2 cognition compared with baseline cognition. There was no sex difference in prediction accuracy for any model or cognitive measure after multiple comparisons correction with FDR *q* < 0.05.

### Models trained on baseline FC to predict baseline cognition improve in accuracy when applied to Year 2 FC and Year 2 cognition

Given the high similarities across the PNF matrices of all three models (Fig. 4c), we hypothesized that the model trained on baseline data would perform well when applied to Year 2 data. Surprisingly, we found that the model trained on baseline FC to predict baseline cognition showed improved accuracy when applied to predict Year 2 cognition from Year 2 FC for PC1 (FDR *q* < 0.05; Fig. 4d).

The other seven cognitive measures showed a similar pattern, although not all differences were significant (Fig. S8). This “model transfer” result suggests that the multivariate relationship between brain and cognition is similar at baseline and Year 2, but the strength of the relationship increases over time, yielding better prediction accuracy. This suggests that early established functional architecture provides a foundation that becomes increasingly effective in supporting cognitive ability.

We note that head motion and head motion variability decreased with age (Table 1), as reflected by reductions in both mean framewise displacement (FD) and the standard deviation of FD (mean FD: *t* = −12.9, *p* < 0.001; std FD: *t* = −13.9, *p* < 0.001). Higher cognitive ability was also associated with lower average head motion and reduced motion variability across participants (Table S2). To ensure that our results were not due to better quality fMRI data at Year 2, we repeated the “model transfer” analysis in this section using a subset of participants with comparable motion levels across the two time points (*p* = 0.18), yielding similar conclusions (Fig. S9).

In addition, we also used Year 2 FC to predict baseline cognitive ability (FCY2 → CogY0). If the higher prediction accuracy at Year 2 were primarily driven by lower motion or improved fMRI quality, then FCY2 should outperform FCY0 in predicting CogY0. However, prediction accuracies of FCY2 → CogY0 were similar to the baseline model (FCY0 → CogY0), but lower than the prediction of Year 2 cognition (FCY2 → CogY2) across all cognitive measures (Table S3). Together with the previous section, these findings collectively suggest enhanced FC–cognition relationships during development that cannot be attributed to head motion.

In sex-stratified analyses, model transfer was also significantly better than chance for all cognitive measures for both females (Fig. S10) and males (Fig. S11). Similar to the full sample combining both males and females, model transfer prediction performance in Year 2 data was generally better than baseline data. There was no sex difference in model transfer prediction performance between males and females after multiple comparisons correction with FDR *q* < 0.05.

### Baseline FC is more predictive of cognition at Year 2 than longitudinal FC change, even accounting for reliability differences

So far, we have demonstrated that there is a strong cross-sectional relationship between FC and cognition, and the relationship strengthens with development (Fig. 4). We hypothesized that longitudinal FC change might also predict future cognitive outcomes.

**Fig. 5:**
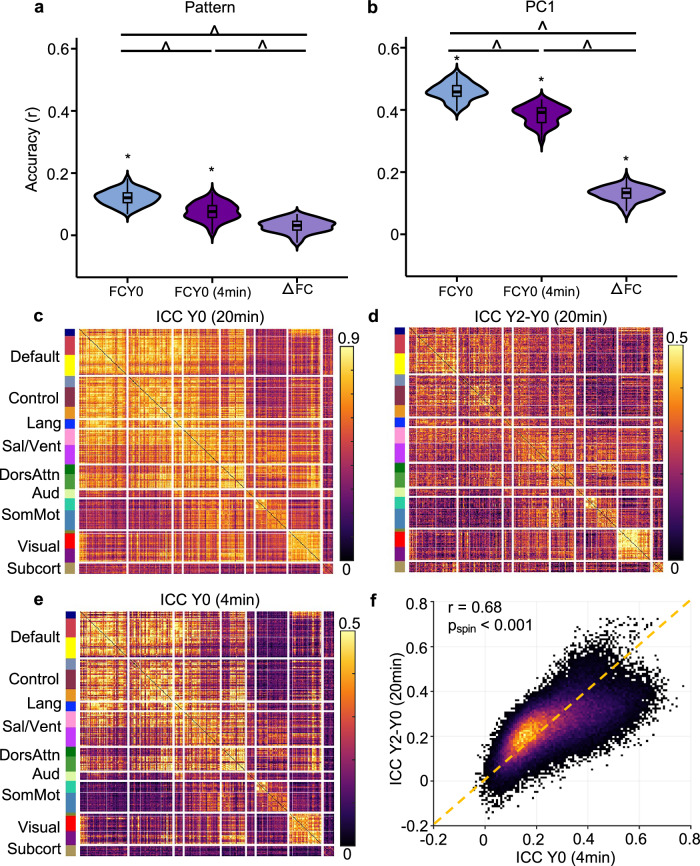
Cross-sectional baseline FC outperforms longitudinal FC change in predicting cognition at Year 2 even accounting for reliability differences. **a** Comparison of prediction accuracies for the Pattern Comparison Processing Speed Test (Pattern) at Year 2 across three models. “FCY0” refers to the model trained on baseline FC to predict Year 2 cognition (FCY0 → CogY2, blue). “FCY0 (4 min)” uses baseline FC computed from the first 4 min of fMRI data to predict Year 2 cognition (FCY0 (4 min) → CogY2, purple). “ΔFC” employs FC change (between Year 2 and baseline) to predict cognition at Year 2 (ΔFC → CogY2, light purple). Each value in the violin plot represents accuracy (*r*) for a single cross-validation fold. Inset box plots indicate the median (centre line), interquartile range (25th–75th percentile; box), and whiskers extending to 1.5× the interquartile range, with outliers not shown. Asterisks (*) denote above-chance prediction after multiple comparisons correction (FDR *q* < 0.05). Carets (^) denote statistically significant differences between models based on the corrected resampled *t*-test (FDR *q* < 0.05). **b** Same as panel (a), but for the first principal component (PC1) of the seven cognitive measures. Fig. S12 repeats panels **a** and **b** for other six cognitive measures. Visualization elements are as in (**a**). **c** Estimated intra-class correlation (ICC) for baseline FC based on 20 min of fMRI data. **d** Estimated ICC for FC change based on 20 min of fMRI data. **e** Estimated ICC for baseline FC based on 4 min of fMRI data. **f** Comparable ICCs were observed for 4-min baseline FC and 20-min FC change. Each dot represents the ICC for an FC edge, computed from baseline (*x*-axis) and FC change (*y*-axis); dot colour indicates point density, with warmer colours reflecting a higher concentration of edges. ICCs were strongly positively correlated (*r* = 0.68 without subcortical regions; *r* = 0.67 with subcortical regions). Statistical significance (one-tailed test) was determined using a spatial permutation (“spin”) test (see the “Methods” section)^90,91^. The yellow dashed line denotes the identity line (*y* =* x*) for reference.

Longitudinal FC change was able to predict all cognitive measures (except for the Pattern Comparison Processing Speed Task) at Year 2 (FDR *q* < 0.05; Figs. 5a and b and S12). However, the best prediction accuracy was only *r* = 0.13 (for PC1). In general, baseline FC outperformed longitudinal FC change in predicting all cognitive measures at Year 2. Predictive network features (PNFs) for PC1 were modestly similar across the two models (*r* = 0.33, *p*_spin_ < 0.001). Similar conclusions were obtained (Fig. S13), when using rate of FC change between Year 2 and baseline (instead of FC change) to predict cognitive measures at Year 2.

The lower predictive accuracy for longitudinal FC change might be due to the lower reliability of FC change compared with cross-sectional FC. We can mathematically express the reliability of FC change in terms of the reliability of baseline FC, reliability of Year 2 FC, and the similarity between the FC of the two timepoints (Eq. (4) in the “Methods” section). Indeed, the high FC stability between baseline and Year 2 (Fig. 3c) is detrimental to the reliability of FC change.

To estimate the reliability of baseline FC (Fig. 5c) and longitudinal FC change (Fig. 5d), we split the four resting-state fMRI runs from each participant into two groups of two runs each, treating them as test and retest sessions. A formula relating FC reliability and scan duration^38^ was then used to extrapolate the reliability estimates to 20 min. Since both test and retest data were acquired within the same session, these estimates likely represent an upper bound on true test-retest reliability.

As expected, the reliability of baseline FC (Fig. 5c) was substantially higher than the reliability of longitudinal FC change (Fig. 5d): mean of 0.56 vs. 0.24, respectively. To assess whether lower reliability might account for the weaker prediction performance of longitudinal FC change, we artificially shortened the fMRI scan duration used to compute baseline FC from 20 to 4 min. The resulting reliabilities were now similar between the 4-min baseline FC and the longitudinal FC change (Fig. 5d–f), with both showing a mean of 0.24 and a correlation of 0.68 (*p*_spin_ < 0.001).

Unsurprisingly, 4-min baseline FC generally showed lower predictive performance than 20-min baseline FC, but was still better than longitudinal FC change (FDR < 0.05; Figs. 5a, b and S12). This implies that the lower prediction accuracy of longitudinal FC change cannot be fully explained by lower reliability. Collectively, our results suggest that stable individual differences in baseline FC exert a stronger influence on future cognitive outcomes than changes in FC over time.

Consistent with the full sample, both baseline FC and 4-min baseline FC were better than longitudinal FC change in predicting Year 2 cognition for both females (Fig. S14) and males (Fig. S15). There was no sex difference in prediction performance for all predictive models after multiple comparisons correction with FDR *q* < 0.05.

### Baseline FC and longitudinal FC change weakly predict cognitive change

**Fig. 6:**
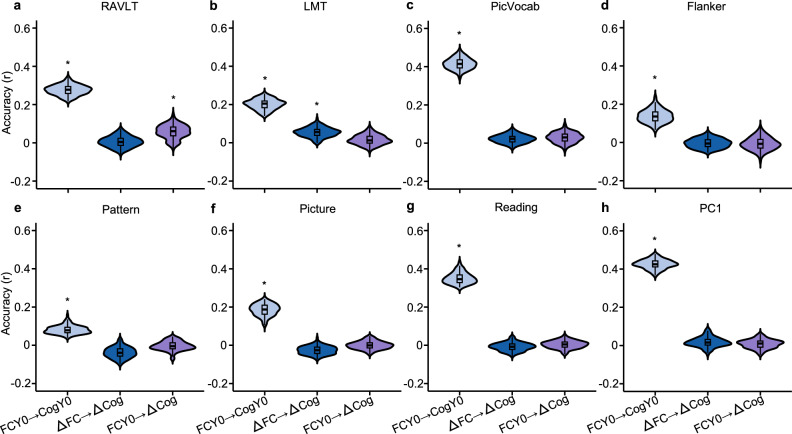
Limited prediction of cognitive change from baseline FC and longitudinal FC change. **a** Prediction accuracy for Rey Auditory Verbal Learning Test (RAVLT; verbal memory). Baseline FC and longitudinal FC change (ΔFC) are used to predict cognitive change (ΔCog). Predictions of baseline cognition from baseline FC are also shown for reference. Colours indicate different prediction settings: baseline FC predicting baseline cognition (FCY0 → CogY0, light blue), longitudinal FC change predicting cognitive change (ΔFC → ΔCog, blue), and baseline FC predicting cognitive change (FCY0 → ΔCog, purple). Each value in the violin plot represents prediction accuracy (*r*) for a single cross-validation fold. Inset box plots indicate the median (centre line), interquartile range (25th–75th percentile; box), and whiskers extending to 1.5× the interquartile range, with outliers not shown. Asterisks (*) denote above-chance prediction after multiple comparisons correction (FDR *q* < 0.05). **b** Prediction accuracy for Little Man Task (LMT; spatial reasoning). **c** Prediction accuracy for Picture Vocabulary Task (PicVocab; vocabulary). **d** Prediction accuracy for Flanker Task (Flanker; executive function). **e** Prediction accuracy for Pattern Comparison Processing Speed Test (Pattern; processing speed). **f** Prediction accuracy for Picture Sequence Memory Test (Picture; episodic memory). **g** Prediction accuracy for Oral Reading Recognition Task (Reading; reading ability). **h** Prediction accuracy for the first principal component (PC1) of the seven cognitive measures. Visualization elements for panels **b**–**h** are as in (**a**).

We have shown that baseline FC robustly predicted future cognitive ability at Year 2. Longitudinal FC change was also able to predict future cognition at Year 2, though not as well as baseline FC. Here, we further explored whether baseline FC and longitudinal FC change could predict cognitive change between baseline and Year 2. We found that baseline FC could only predict cognitive change for the Rey Auditory Verbal Learning Task (RAVLT; FDR *q* < 0.05; Fig. 6). Similarly, longitudinal FC change could only predict cognitive change of the Little Man Task (LMT; FDR *q* < 0.05; Fig. 6).

Importantly, we note that baseline FC did not perform better than longitudinal FC change, suggesting that the weak prediction cannot be fully explained by the lower reliability of longitudinal FC change. However, the weak prediction could be due to the lower reliability of cognition change compared with baseline cognition. Similar conclusions were reached when using the longitudinal rate of changes in FC and cognition between baseline and Year 2 (Fig. S16).

Consistent with the full sample, longitudinal cognitive change was poorly predicted by baseline FC and longitudinal FC change for both females (Fig. S17) and males (Fig. S18). Due to the smaller sample size, no prediction of longitudinal cognitive change was better than chance. There was also no sex difference in prediction performance for all cognitive measures after multiple-comparisons correction with FDR *q* < 0.05.

### Convergent and divergent predictive network features between cross-sectional and longitudinal estimates of brain–cognition relationship

Consistent with previous studies^10–13^, we found strong FC–cognition relationship at baseline (Fig. 4). For example, individuals with stronger FC within the salience network at baseline also exhibited better cognitive performance at baseline (Fig. 4c). Assuming a causal relationship, we might expect individuals with greater increase in salience network FC to exhibit a greater improvement in cognitive performance between the two time points. Since longitudinal FC change only significantly predicted changes in the Little Man Task (LMT; Fig. 6b), we focused our analysis on LMT.

**Fig. 7:**
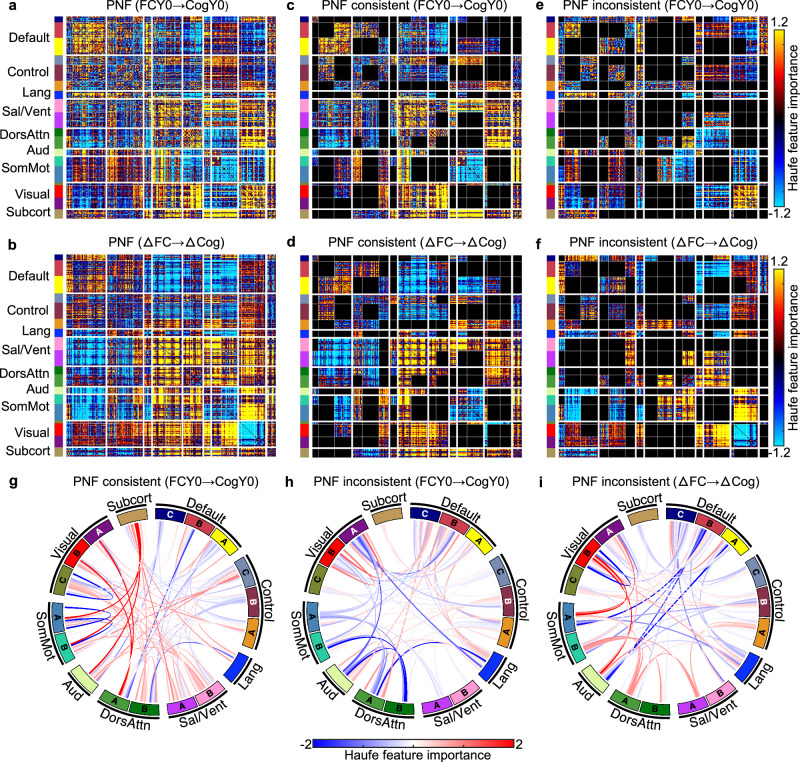
Convergent and divergent predictive network features (PNFs) between cross-sectional and longitudinal estimates of the FC–cognition relationship. **a** PNFs from the cross-sectional model using baseline FC to predict baseline Little Man Task (LMT) score (FCY0 → CogY0). **b** PNFs from the longitudinal model using changes in FC to predict changes in LMT performance (ΔFC → ΔCog) across the two timepoints. **c** Network blocks with consistent PNFs in the cross-sectional model. **d** Network blocks with consistent PNFs in the longitudinal model. PNFs were considered consistent if the average feature value for a network block had the same sign in both cross-sectional and longitudinal models. **e** Network blocks with inconsistent PNFs in the cross-sectional model. **f** Network blocks with inconsistent PNFs in the longitudinal model. PNFs were considered inconsistent if the average feature value had opposite signs across models. **g** Same as (**c**), visualized using a chord diagram. **h** Same as (**e**), visualized using a chord diagram. **i** Same as (**f**), visualized using a chord diagram. Colour indicates Haufe-transformed feature importance (red: positive; blue: negative). For visualization purposes, each predictive network feature matrix was normalized by dividing all values by the standard deviation of the entire matrix.

More specifically, we computed the predictive network features (PNFs) for the model using baseline FC to predict baseline LMT score (Fig. 7a), as well as for the model using longitudinal FC change to predict longitudinal LMT performance change across the two timepoints (Fig. 7b). The two PNFs were only weakly correlated (*r* = 0.14). A spin test excluding subcortical regions confirmed statistical significance (*r* = 0.1; *p*_Spin_ < 0.001).

Regardless of statistical significance, there were clear convergences (Fig. 7c and d) and divergences (Fig. 7e and f) between the cross-sectional and longitudinal PNFs. For example, individuals who exhibited greater increases in FC within the salience network over time also showed larger improvements in LMT performance (Fig. 7d). This aligns with the cross-sectional finding that stronger salience network FC at baseline was associated with better LMT performance (Fig. 7c), indicating convergence between cross-sectional and longitudinal estimates of brain–cognition relationship (Fig. 1c). A chord diagram was used to visualize these convergent links (Fig. 7g).

In contrast, individuals with stronger baseline FC within the visual networks tended to perform

better on the LMT at baseline (Fig. 7e). However, greater longitudinal reduction in visual connectivity predicted larger improvements in LMT performance (Fig. 7f), reflecting divergence between cross-sectional and longitudinal estimates of brain–cognition relationship (Fig. 1d). Chord diagrams were used to highlight the divergent links across models (Figs. 7h and i).

**Fig. 8:**
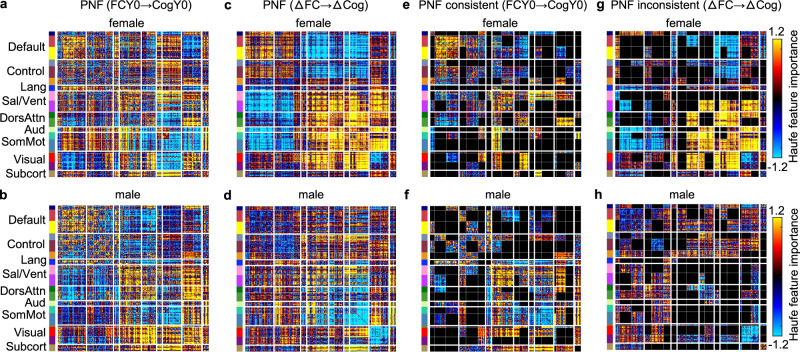
Convergent and divergent predictive network features (PNFs) between cross-sectional and longitudinal estimates of FC–cognition relationship within each sex. PNFs from cross-sectional models using baseline FC to predict baseline Little Man Task (LMT) score (FCY0 → CogY0) in **a** females and **b** males separately. PNFs from longitudinal models using changes in FC to predict changes in LMT performance (ΔFC → ΔCog) across the two timepoints in **c** females and **d** males separately. Network blocks with consistent PNFs between cross-sectional and longitudinal models in **e** females and **f** males. Network blocks with inconsistent PNFs between cross-sectional and longitudinal models in **g** females and **h** males. PNFs were considered consistent if the average feature value for a network block had the same sign in both cross-sectional and longitudinal models. Colour indicates Haufe-transformed feature importance (red: positive; blue: negative). For visualization purposes, each predictive network feature matrix was normalized by dividing all values by the standard deviation of the entire matrix. Thresholded PNFs are shown in Fig. S19.

Consistent with results from the full sample, models trained separately for each sex showed both convergent and divergent predictive network features (PNFs) between cross-sectional and longitudinal analyses. For example, greater FC within the salience network predicted better baseline LMT performance in the cross-sectional models (Fig. 8a and b), and greater longitudinal increases in salience network FC predicted greater improvements in LMT performance (Fig. 8c and d), indicating feature-level convergence between cross-sectional and longitudinal models (Fig. 8e and f). In contrast, although greater visual network FC predicted better baseline LMT performance for both sexes in the cross-sectional models (Fig. 8a and b), greater longitudinal decreases in visual network FC predicted better longitudinal improvement in LMT performance (Fig. 8c and d), reflecting divergence between cross-sectional and longitudinal models (Fig. 8g and h).

Predictive network features were substantially more similar between males and females in the cross-sectional models (*r* = 0.5; Fig. 8a and b) than in the longitudinal models (*r* = −0.03; Fig. 8c and d), indicating greater sex-specific divergence in longitudinal predictions. For example, in both females and males, weaker baseline FC within the somatomotor network predicted better baseline LMT performance in the cross-sectional models (Fig. 8a and b). However, in females, greater longitudinal increases in somatomotor FC predicted greater improvement in LMT performance (Fig. 8c), diverging from the cross-sectional pattern (Fig. 8g). In contrast, in males, greater longitudinal decreases in somatomotor FC predicted greater improvement in LMT performance (Fig. 8d), converging with the male-only cross-sectional model (Fig. 8f).

For completeness, PNFs for the other seven cognitive measures are shown in Fig. S20. Correlations between cross-sectional and longitudinal models of brain–cognition relationship ranged from −0.08 to 0.62. Consistent with LMT, there were clear convergences and divergences between cross-sectional and longitudinal PNFs.

### Replication in a more representative subsample

Filtering participants based on missing data and head motion can reduce the representativeness of the data sample^11,39–42^. To ensure our results are not due to a non-representative sample, we constructed a matched subsample from the sample used in the main analyses by jointly matching age, sex, household income, race/ethnicity, and baseline cognition (PC1) to the full ABCD baseline cohort. This procedure yielded a matched subsample of 2020 participants that approximated the demographic and cognitive distributions of the full ABCD baseline cohort. Included and excluded participants did not differ significantly on any matching variables after multiple comparisons correction with FDR *q* < 0.05 (Table S4).

Across all primary analyses, results obtained from the representativeness-matched subsample closely mirrored those from the full analytic cohort (Figs. S21–S27). More specifically, FC stability and longitudinal change mirrored the S–A axis (Fig. S22). Furthermore, the relationship between FC and cognition strengthened with development (Figs. S23 and S24). Baseline FC was more predictive of cognition at Year 2 than longitudinal FC change (Fig. S25). Both baseline FC and longitudinal FC change weakly predicted cognitive change (Fig. S26). Finally, we observed convergent and divergent predictive network features between cross-sectional and longitudinal models (Fig. S27).

## Discussion

Longitudinal analyses of rs-fMRI and cognitive data from the ABCD Study show that, while cognitive performance improves on average over time, individual differences in cognition remain highly stable. FC exhibits both developmental change and individual stability that are organized along the sensorimotor–association (S–A) axis. Baseline FC predicts future cognitive ability better than it predicts baseline cognitive ability. Prediction accuracy also improves when models trained on baseline data are applied to Year 2 FC and cognition. These results suggest that brain–cognition relationships become more pronounced during early adolescence. In addition, baseline FC surpasses longitudinal FC change in predicting future cognitive ability. Neither baseline FC nor FC change can predict cognitive change well. Beyond differences in predictive power, we also found both overlapping and distinct network predictors across cross-sectional and longitudinal predictive models. Overall, these findings indicate that early adolescent brain–cognition development is driven more by stable individual differences in network organization than by short-term FC changes.

### Sustained early individual differences despite longitudinal change in cognition and FC

Developmental science seeks to uncover both stability and change in individual characteristics over time^43–45^. In our study, a high similarity in cognitive profiles across two timepoints (Fig. 2a) alongside the longitudinal increase in cognitive performance (Fig. 2c) reflect developmental stability and change, respectively. The brain functional network architecture, as measured by resting-state FC, also exhibited strong developmental stability (Fig. 3c) and change (Fig. 3f).

Intriguingly, FC stability and change showed regional variations along the S–A axis (Fig. 3d and g). The S–A axis represents a key axis of hierarchical cortical organization, spanning from primary sensorimotor to transmodal association cortices^33,34^. Many cross-sectional studies have shown that the development of numerous functional and structural brain properties is organized along the S–A axis^46–50^. The current study extends these previous studies by providing longitudinal evidence of greater increases (and lower stability) in sensory-motor regions and greater decreases (and higher stability) in association cortex.

Beyond group-level stability and changes, we also observed substantial individual differences in cognitive (Fig. 2e) and functional network (Fig. 3i) changes. Intriguingly, there were some participants whose patterns of cognitive improvements (Fig. 2g) and FC changes (Fig. 3k–n) were uncorrelated with those of other participants. Previous studies have found that sensorimotor and visual regions exhibit lower inter-individual FC variability than association cortex^51–54^. In contrast, we found that inter-individual differences in FC development were especially pronounced in sensorimotor and visual cortex (Fig. 3j). However, the regional variation did not exactly reflect the S–A axis because certain regions of the default network, such as the medial prefrontal cortex also exhibit high variability in longitudinal FC change (Fig. 3j). Therefore, our results reveal divergences between inter-individual variation in cross-sectional FC and inter-individual variation in longitudinal FC change.

### Strengthened the relationship between FC and cognition during development

Previous studies have shown that brain functional connectomes can reliably predict current and future cognitive performance in children, adolescents, and adults^11–14,17,55^. We extend previous findings by demonstrating that baseline FC more accurately predicts future cognitive performance than current cognition, and models of brain–cognition relationship at baseline generalize better when applied to data two years later (Fig. 4). The results hold when using a subset of participants with comparable head motion (Fig. S9). Thus, our study extends prior understanding by revealing that the brain–cognition relationships strengthen during the transition from childhood to adolescence. These results suggest that functional brain architecture may serve as a stable scaffold that increasingly supports cognitive abilities over time.

### Dominant role of stable individual differences in predicting future cognitive outcomes

A recent study linked individual variability in longitudinal FC change with demographic and maturational factors^56^. We build on this work by examining how baseline FC and longitudinal FC changes relate to future cognitive ability. Two prior studies in infancy had divergent findings: one study reported stronger cross-sectional associations^57^, while another study found stronger longitudinal effects^58^. These discrepancies may reflect differences in study design, including small sample sizes that can inflate effect sizes^59^, as well as variation in the target region of interest (e.g., amygdala vs. hippocampus) and reliance on univariate analyses. By leveraging a large sample and multivariate prediction methods, our study provides definitive evidence on the relative roles of cross-sectional and longitudinal FC in predicting cognitive outcomes.

Our findings showed that longitudinal change in FC between baseline and Year 2 can predict cognitive performance at Year 2. However, baseline FC outperformed longitudinal FC change in predicting Year 2 cognition (Fig. 5a and b). Given that estimates of longitudinal change are often less reliable than cross-sectional estimates due to accumulated measurement noise^60–63^, we developed a method to estimate the reliability of FC change and baseline FC. Even after controlling for reliability, baseline FC still yielded higher predictive accuracy. This suggests that the superior predictive power of baseline FC cannot be fully attributed to reliability differences. Instead, it points to the greater influence of stable individual differences in FC on future cognitive outcomes.

Prior work has shown that individual differences in functional connectome organization are already evident in the third trimester and closely resemble adult-like patterns^51,52^. These early-emerging features, likely shaped by neurogenesis and genetic programming, tend to be relatively stable and less susceptible to postnatal environmental influences^51,64^. In contrast, longitudinal FC change during the transition from childhood and adolescence may reflect more transient, state-dependent dynamics shaped by environmental variability and experience. This contrast may account for the stronger predictive power of baseline FC compared to FC change in forecasting future cognitive outcomes.

Our study also provides estimates of the reliability of longitudinal FC change. Based on 20-min scans, longitudinal FC change exhibits an ICC of 0.24, while baseline FC exhibits an ICC of 0.56. Because the reliability of longitudinal FC change is determined by the reliability of the baseline FC and Year 2 FC, our study suggests that the need for even longer scan duration in longitudinal studies to achieve more reliable estimates of longitudinal FC change.

### Convergent and divergent brain–cognition relationships across cross-sectional and longitudinal models

Surprisingly, longitudinal FC changes showed limited ability to predict cognitive change, with only the Little Man Task reaching statistical significance (Fig. 6). This stands in sharp contrast to the strong cognitive predictive performance achieved using FC, revealing a prediction-level divergence between cross-sectional and longitudinal models (Fig. 1b). Notably, this divergence cannot be fully explained by the low reliability of longitudinal FC change, given that baseline FC is more reliable but still fails to predict cognitive growth. One possibility is that both FC and its longitudinal changes may be relatively insensitive to the dynamic neurocognitive processes that drive cognitive growth during this transitional period. Furthermore, cognitive development may be strongly shaped by external influences, such as environmental conditions, learning opportunities, and social experiences, which may not be fully captured by FC measures.

However, another possibility is that longitudinal cognition changes are less reliable than baseline cognition, which will lead to worse prediction performance^65,66^. Indeed, the formula relating cross-sectional FC and longitudinal FC change (Eq.(4) in the “Methods” section) is also applicable to cognition. The strong correlation of cognitive measures between baseline and Year 2 (Fig. 2a) will greatly reduce the reliability of cognitive change. Because of this potential “confound”, we should not over-interpret prediction accuracy differences between cross-sectional (FCY0 → CogY0) and longitudinal (ΔFC → ΔCog) models of brain–cognition relationship (Fig. 6). However, we could interpret the Haufe-transformed predictive network features of cognitive measures that were successfully predicted (Fig. 7).

Beyond prediction strength, we observed both convergence and divergence between cross-sectional and longitudinal predictive network features, extending the classical Simpson’s paradox^27^ (Fig. 1a) into a multidimensional developmental context. Stronger baseline salience network FC was associated with better baseline LMT performance, and greater increases in salience network FC over time predicted greater LMT improvement, reflecting convergence between cross-sectional and longitudinal predictive models (Fig. 1c). In contrast, while stronger baseline visual FC was associated with better baseline LMT performance, greater reductions in visual connectivity over time predicted greater LMT improvement, indicating divergence between the two estimates (Fig. 1d). These findings provide an empirical demonstration of how cross-sectional and longitudinal brain–cognition relationships can converge and diverge, underscoring the need to disentangle their contributions to cognitive development.

### Sex differences in cognition, FC, and FC–cognition relationships

Previous studies have documented sex differences in cognition, brain function, and their interrelationships^67–72^. However, much of this literature relies on cross-sectional designs spanning broad and heterogeneous age ranges, limiting inferences into sex-specific developmental trajectories. Our study systematically examined longitudinal developmental patterns in cognition, FC, and FC–cognition relationships across sexes during the transition from childhood to early adolescence.

With respect to cognitive development, we did not observe a significant overall longitudinal improvement in RAVLT performance, consistent with a previous ABCD study^73^. Notably, however, we detected a significant sex-by-age interval interaction, with males showing a positive slope and females showing a negative slope over time (Fig. 2). These opposing trajectories likely attenuated sensitivity to longitudinal effects when sexes were analysed together. In addition, sex differences in the variance of individual-level longitudinal cognitive change were observed for RAVLT and the Pattern Comparison Processing Speed Test, indicating measure-specific heterogeneity rather than pervasive sex differences. In the case of longitudinal FC change, females generally exhibited a slower rate of FC decrease than males, especially in the association cortex (Fig. 3).

Turning to brain–behaviour relationships, prior cross-sectional analyses of ABCD data have reported some sex differences^71,74^. Consistent with these findings, we observed sex-specific patterns in cross-sectional predictive models (Fig. 8a and b), alongside substantial overlap in predictive network features, paralleling results from cross-sectional studies in young adults^75^. In contrast, predictive network features diverged more strongly between males and females in the longitudinal models (Fig. 8c and d), giving rise to sex-specific convergences and divergences when compared with cross-sectional models (Fig. 8e–h).

### Limitations and future work

Our analysis was limited to the first two timepoints of the ABCD Study. While this restricts our ability to capture nonlinear or individual-specific trajectories of FC and cognitive development^76,77^, the dataset remains the largest longitudinal sample currently available for investigating brain–cognition relationships during the transition from childhood to early adolescence. Although change estimates derived from only two timepoints may be less precise than those based on more frequent measurements, our findings provide an important early benchmark and a foundation for predictive modelling of developmental processes. Future work leveraging additional timepoints from the ABCD study will be able to explore trajectories that extend further into adolescence.

In summary, leveraging large-scale longitudinal data, we show that brain network organization and cognition change together across early adolescence, yet future cognitive outcomes are shaped predominantly by enduring individual differences rather than short-term fluctuations in functional connectivity. By contrasting cross-sectional and longitudinal predictive frameworks, we further reveal a multivariate manifestation of Simpson’s paradox, in which convergent and divergent predictive network features emerge depending on the temporal lens of analysis. Together, these results highlight the importance of stable functional connectome architecture as a central scaffold for cognitive development during early adolescence, while cautioning against over-interpreting short-term neural change as a primary driver of cognitive growth.

## Methods

### Participants

We considered individuals with rs-fMRI and cognition tests from the ABCD Study across 21 sites. Sex was defined as biological sex at birth, as reported by caregivers at baseline. Ethical approval was granted by the Institutional Review Board (IRB) at the University of California, San Diego, as well as by the IRBs of each participating study site. Written informed consent was obtained from parents and guardians, and assent was obtained from all participants. Participants were compensated for their participation as part of the ABCD study protocol. At the start of this study, the third time point of ABCD imaging data was still in its initial stage. Thus, we included only participants with rs-fMRI and cognition data at baseline and Year 2, ensuring that all participants were unrelated and remained at the same site across the two time points. Specifically, after rs-fMRI quality control of both time points, 4615 participants remained. Excluding participants lacking cognition measures at both time points reduced the sample size to 3455, and further restricting to unrelated participants resulted in 3147 participants. Finally, we excluded participants who were scanned at different sites across the two time points and those from sites with fewer than 10 participants. Our final sample comprised 2949 individuals (Table 1).

### Imaging data preprocessing

Minimally processed T1 and rs-fMRI data were utilized. Details about acquisition protocol and minimal processing can be found elsewhere^30,78^. Resting-state fMRI data preprocessing was in line with our previous study^11,55^. Specifically, (1) rs-fMRI data were aligned to T1 images using boundary-based registration^79^. (2) Respiratory pseudo-motion was filtered by applying a band-stop criteria of 0.31–0.43 Hz^80^. (3) Volumes with framewise displacement (FD) > 0.3 mm or voxel-wise differentiated signal variance (DVARS) > 50 were flagged. Then, each flagged frame, along with the one immediately before and the two immediately after, was censored. Uncensored data segments with fewer than five frames were also censored. (4) Global signal, white matter signal, and ventricular signals, six motion parameters2, as well as their temporal derivatives, were regressed out from the rs-fMRI data, with regression coefficients estimated from uncensored data. (5) The Lomb–Scargle periodogram method^81^ was used to interpolate censored frames. (6) A band-pass filter (0.009–0.08 Hz) was applied. (7) The data were mapped onto fsaverage6 surface space in FreeSurfer and then smoothed with a 6 mm full-width at half-maximum kernel.

### Functional connectivity

We constructed the FC matrix by combining the 400-region Yan parcellation^31^ (Fig. 3a) with 19 subcortical regions of interest (ROIs, Fig. 3b) defined by Fischl et al.^32^. FC was calculated as the Pearson correlation coefficient between the average time series of each ROI pair, resulting in a 419 × 419 FC matrix. Notably, censored frames were excluded when computing FC. We averaged the FC matrices across runs for each participant after Fisher’s *r*-to-*z* transformation and converted back to *r* values after averaging.

### Cognition assessment

We considered seven cognitive tasks administered at both timepoints. Five were drawn from the NIH Toolbox (i.e., Picture Vocabulary Task, Flanker Task, Pattern Comparison Processing Speed Test, Picture Sequence Memory Test, and the Oral Reading Recognition Task)^82^, and two additional tasks included the Rey Auditory Verbal Learning Test (RAVLT) and Little Man Task (LMT)^83^. The Picture Vocabulary Task assesses language skills and verbal intelligence, while the Oral Reading Recognition Task measures reading ability by asking participants to pronounce isolated words. The Pattern Comparison Processing Speed Test evaluates rapid visual processing. The Picture Sequence Memory Test assesses episodic memory through the recall of image sequences. The Flanker Task measures response inhibition and conflict monitoring by requiring participants to modulate responses under congruent versus incongruent conditions. The RAVLT assesses auditory learning, memory, and recognition, whereas the LMT engages visual–spatial processing, specifically mental rotation, across varying levels of difficulty.

Consistent with previous work^84^, we used uncorrected standard scores for each NIH Toolbox task, total correct scores for the RAVLT, and percent correct scores for the LMT in our analyses.

To obtain a measure of overall cognitive ability, we *z*-normalized the seven cognitive measures and then applied principal component analysis to derive the first principal component (PC1) explaining the most variability in cognition across participants. To avoid data leakage, the cognitive component was estimated from 6050 participants at baseline who were not included in the main analysis. The loadings of PC1 were then transferred to derive PC1 for the 2949 participants at baseline and Year 2. We note that the *z*-normalization for the 2949 participants was performed using the mean and standard deviation calculated from the 6050 participants.

### Longitudinal cognitive change

We first applied longitudinal ComBat^85^ to remove site effects from the cognitive scores. Baseline age, age interval between the two timepoints, and sex were included in the ComBat model to preserve biologically meaningful variability. The output consisted of harmonized baseline and Year 2 cognitive scores, with both additive and multiplicative site effects accounted for.

To examine the stability of cognitive performance between baseline and Year 2, for each of the eight cognitive measures, we computed the Spearman’s correlation of the cognitive measures between the two time points (across participants; Fig. 2a). More specifically, we regressed baseline age and sex from the harmonized baseline cognitive scores, as well as Year 2 age and sex from the harmonized Year 2 cognition. Spearman’s correlation was then computed using the resulting residuals.

To examine the longitudinal change in cognitive performance (Fig. 2c), we employed a linear mixed effects model consistent with the longitudinal ComBat model. More specifically, baseline age, age interval between the two timepoints and sex are used as covariates.

1 $${{{{\rm{Cognition}}}}}_{{it}}={\beta }_{0}+{\beta }_{1}{{{{\rm{Age}}}}}_{i1}+{\beta }_{2}\left({{{{\rm{Age}}}}}_{{it}}-{{{{\rm{Age}}}}}_{i1}\right)+{\beta }_{3}{{{{\rm{Sex}}}}}_{i}+{u}_{i}+{\varepsilon }_{{it}},$$

where *i* indexes the *i*th participant, *t* references the time point, *u*_*i*_ is the random intercept and *ε*_*it*_ is the noise term. The *t* statistic for the age interval coefficient (*β*_2_) was subsequently converted to Cohen’s *d* to quantify the longitudinal effect size. Cognition_*it*_ is the cognition of the *i*th participant at timepoint *t* after longitudinal ComBat.

However, we note that Eq.(1) can only capture a group-level estimate of longitudinal change. Therefore, to capture the potential individual differences in longitudinal cognition change, for each participant and each cognitive score, we computed the longitudinal cognition change as: Cognition_*i*2_−Cognition_*i*1,_ where Cognition_*it*_ is the cognition of the *i*th participant at timepoint *t* (from longitudinal ComBat). We then regressed out sex, baseline age and age interval (i.e., between Year 2 and baseline) from the individual-level longitudinal cognition change estimate.

### Longitudinal functional brain network change

We first applied longitudinal ComBat^85^ to remove site effects from each FC entry. Baseline age, age interval between the two timepoints, sex and head motion were included in the ComBat model to preserve biologically meaningful variability. The output consisted of harmonized baseline and Year 2 FC with additive and multiplicative site effects accounted for.

To examine the stability of FC between baseline and Year 2, for each FC entry, we computed the Spearman’s correlation of the FC value between the two time points (across participants; Fig. 3c). More specifically, we regressed baseline age, sex and mean head motion (as measured by framewise displacement FD) from the harmonized baseline FC, as well as Year 2 age, sex and mean head motion (FD) from the harmonized Year 2 FC. Spearman’s correlation was then computed using the resulting residuals.

To examine the longitudinal change in FC (Fig. 3f), we employed a linear mixed-effect model consistent with the longitudinal ComBat model. More specifically, baseline age, age interval between the two timepoints, sex, and mean FD were included as covariates.

2 $${{{{\rm{FC}}}}}_{{it}}={\beta }_{0}+{\beta }_{1}{{{{\rm{Age}}}}}_{i1}+{\beta }_{2}\left({{{{\rm{Age}}}}}_{{it}}-{{{{\rm{Age}}}}}_{i1}\right)+{\beta }_{3}{{{{\rm{Sex}}}}}_{{it}}+{\beta }_{4}{{{{\rm{meanFD}}}}}_{{it}}+{u}_{i}+{\varepsilon }_{{it}},$$

where *i* indexes the *i*th participant, *t* references the time point, *u*_*i*_ is the random intercept and *ε*_*it*_ is the noise term. The *t* statistic for the age interval coefficient (*β*_2_) was subsequently converted to Cohen’s *d* to quantify the longitudinal effect size. FC_*it*_ is the FC value of a particular FC edge of the *i*th participant at timepoint *t* after longitudinal ComBat.

However, we note that Eq. (2) captures only the group-level estimate of longitudinal change. Therefore, to capture the potential individual differences in longitudinal FC change, for each participant and each FC edge, we computed the z value of FC change across the two timepoints:

3 $$z=\frac{{r}_{2}-{r}_{0}}{\sqrt{{s}_{2}+{s}_{0}}},$$

where *r*_2_ and *r*_0_ denote Fisher *r*-to-*z* transformed FC value for the FC edge at Year 2 and baseline, respectively. *s*_2_ and *s*_0_ denote the variance of the FC value for each FC edge at Year 2 and baseline, respectively. The estimated variance needed to account for auto-correlation in the fMRI time series, so we used the MATLAB function xDF.m (https://github.com/asoroosh/xDF) to estimate *r* and *s* for each FC edge at each time point^86^. We then regressed sex, baseline age and age interval (between Year 2 and baseline), mean head motion (FD) at baseline, mean head motion (FD) at Year 2 from the *z*-statistic across participants.

### Using cross-sectional FC to predict cross-sectional cognition

To explore the cross-sectional relationship between FC and cognition, we used (1) baseline FC to predict baseline cognition, and also (2) Year 2 FC to predict Year 2 cognition (Fig. 4a and b). For comparison, we also used baseline FC to predict Year 2 cognition.

For the prediction analysis, we applied the same KRR framework as in our previous studies^11,55^. Briefly, participants from all imaging sites were grouped into 10 “site clusters”, with each cluster comprising all participants from one or more sites and containing at least 280 individuals.

We then implemented a leave-3-site-cluster-out nested cross-validation approach, where 7 random site clusters were used as the training set, and the remaining 3 clusters served as the test set. This process resulted in 120 unique replications, covering every possible split. We emphasize that individuals from the same site were not split across site clusters, so the test set always contains participants not from the same site as the training set.

10-fold cross-validation was performed within the training set to determine the optimal regularization hyperparameter. The best hyperparameter was used to train a final model from the full training set. This final model was then applied to the test set. This procedure was repeated 120 times, covering every possible split.

When using baseline FC to predict baseline cognition, we controlled for sex, baseline age, and baseline head motion. When using Year 2 FC to predict Year 2 cognition, we controlled for sex, Year 2 age, and Year 2 head motion (mean FD). When using baseline FC to predict Year 2 cognition, we controlled for sex, Year 2 age, age interval between the two timepoints, and baseline head motion (mean FD). All regressions were performed on the training set, and the resulting regression coefficients were applied to the test set.

Prediction accuracy was calculated as the Pearson’s correlation between the predicted scores and actual scores within each test set and then averaged across test sets. We note that in this prediction analysis (and all future prediction analyses), longitudinal ComBat was not performed because ComBat requires FC and/or cognition from all sites (including those from the test set) to be included in the mixed effects model, resulting in test set leakage.

Not performing any harmonization would, in theory, hurt our prediction accuracy, as opposed to inflating our prediction accuracy. Furthermore, by performing leave-3-site-cluster-out cross-validation, our prediction procedure is generalizable to hypothetical new sites in which there is only a single individual, so harmonization cannot be performed. Therefore, we believe that our approach is a reasonable (i.e., conservative) course of action.

### Model interpretation

To interpret feature importance in the predictive models (Fig. 4c), we employed the Haufe transformation to yield a 419 × 419 predictive network feature (PNF) matrix for each cognitive measure^11,35,36,55^. A positive PNF indicates that a higher FC value was associated with a higher predicted cognitive score.

### Model transfer across time points

We have previously trained a model to use baseline FC to predict baseline cognition. Here, we examined whether the model can be used to predict cognition at Year 2 (Fig. 4d) without any further tuning.

More specifically, for a given split of the 10 site clusters into a training set (7 site clusters) and a test set (3 site clusters), we trained the baseline model on the baseline training set, after regressing out sex, baseline age and baseline head motion from baseline cognition. The regression coefficients for sex, age and head motion (from the training set) were then applied to regress sex, Year 2 age and Year 2 head motion from Year 2 cognition in the test set. Finally, we used the baseline model to predict Year 2 cognition (after regressing out the covariates) from Year 2 FC.

To control for the influence of head motion on our results, we selected a subset of participants (*n* = 2642) with matched mean FD across the two time points and repeated the entire model transfer analyses (Fig. S9).

### Using longitudinal FC change (delta) to predict Year 2 cognition

We explored the use of FC change (delta) to predict cognition at Year 2 using KRR (Fig. 5a and b). FC change (or delta) is defined as the difference between Year 2 FC and baseline FC. Here, we controlled for sex, baseline age, age interval, baseline head motion (mean FD) and Year 2 head motion (mean FD). All regressions were performed on the training set, and the resulting regression coefficients were applied to the test set.

As a control analysis, we repeated the analyses using the rate of FC change (Fig. S13). Rate of FC change was defined as (FCY2−FCY0)/(AgeY2−AgeY0). The same set of nuisance regressors was used.

### Mathematical relationship between the reliability of FC change and reliability of cross-sectional FC

As shown in the main results, longitudinal FC change was less effective than baseline FC in predicting Year 2 cognition. One contributing factor could be the lower reliability of FC change, compared with cross-sectional baseline FC. To illustrate this, we derived the mathematical relationship between the reliability of FC change and that of cross-sectional FC^63^.

Let *R*_1_ be the reliability of FC at timepoint 1 and *R*_2_ be the reliability of FC at timepoint 2. Let *V*_1_ be the variance of FC at timepoint 1 and *V*_2_ be the variance of FC at timepoint 2. Let *ρ* be the empirical Pearson’s correlation between the FC of the two timepoints. The reliability of the longitudinal FC change, denoted *R*_D_, can then be expressed as (see Supplementary Methods):

4 $${R}_{{{{\rm{D}}}}}=\frac{{R}_{1}{V}_{1}+{R}_{2}{V}_{2}-2\rho\sqrt{{V}_{1}{V}_{2}}}{{V}_{1}+{V}_{2}-2\rho\sqrt{{V}_{1}{V}_{2}}}.$$

A higher *ρ* will lead to lower FC change reliability (*R*_D_). Indeed, the high FC stability between baseline and Year 2 (Fig. 3c and d) implies a large *ρ*, and consequently, a lower FC change reliability (*R*_D_).

### Empirically estimating the reliability of cross-sectional FC and longitudinal FC change

To investigate whether the lower FC change reliability could explain the weaker prediction, we first empirically estimate the reliability of cross-sectional (baseline) FC and longitudinal FC change. However, there is no longitudinal test–retest data in the ABCD Study, so we developed a method to estimate cross-sectional FC reliability and longitudinal FC change reliability.

More specifically, to estimate cross-sectional (baseline) FC reliability, we chose a subset of participants with 4 runs after quality control (*n* = 897) and divided the 4 runs into 2 groups comprising the first two runs and the last two runs. We can compute FC twice, once using the first two runs (10 min) and once using the last two runs (10 min). By treating the two FC estimates as test-retest data, we can compute ICC using the one-way random effects ANOVA model^87^:

5 $${{{\rm{ICC}}}}=\frac{{{\mbox{MSB}}}-{{\mbox{MSW}}}}{{{\mbox{MSB}}}+(K-1){{\mbox{MSW}}}},$$

where MSB denotes the mean square between-participants variability, MSW denotes mean square within-participant variability, and *K* is the number of repeat measures (which is two in this case). However, this ICC estimate (based on 10 min of fMRI) is probably smaller than the ICC of FC based on the full 20 min of fMRI.

To estimate the ICC of the baseline FC based on the full 20 min of fMRI, we adopt the following approximation^38^:

6 $${R}_{T}=\frac{{s}^{2}}{{s}^{2}+\frac{{t}^{2}}{T}},$$

where *R*_*T*_ is the reliability based on *T* min of fMRI. ${t}^{2}/T$ represents the variance of the fMRI noise, which decreases with scan time *T*, but the variance is modulated by ${t}^{2}$ (due to autocorrelation in the fMRI signal). Finally, ${s}^{2}$ is the true between-participant variability. As scan time *T* becomes large, the noise component diminishes, and reliability asymptotically approaches 1.

We would like to use Eq. (6) to infer the reliability of cross-sectional (baseline) FC based on 20 min of fMRI, but ${s}^{2}$ and ${t}^{2}$ are unknown. Therefore, we computed ICC of cross-sectional FC using 2, 4, 6, 8 and 10 min of fMRI. We then applied Eq. (6) to estimate ${s}^{2}$ and ${t}^{2}$, and substituted *T* = 20 min into Eq. (6) to obtain the cross-sectional (baseline) FC reliability based on 20 min of fMRI. Note that ${s}^{2}$ and ${t}^{2}$ are generally different for different FC edges, so we repeated the whole procedure independently for all FC edges. The fit of Eq. (6) is extremely good in practice, with a median coefficient of determination (COD) of 0.95.

We repeated the same procedure to obtain FC reliability at Year 2, obtaining a strong fit (median COD = 0.94). Finally, we applied Eq. (4) to obtain the reliability of longitudinal FC change.

### Controlling for reliability when comparing baseline FC and longitudinal FC change for predicting Year 2 cognition

As shown in the main results, longitudinal FC change was less predictive of Year 2 cognition than baseline FC. To test whether this prediction gap could be attributed to the lower reliability of longitudinal FC change, we reduced the scan duration used to compute baseline FC so that its reliability would match that of longitudinal FC change. Specifically, we identified the value of *T* in Eq. (6) that equated baseline FC reliability to longitudinal FC reliability. This value of *T* varied across FC edges, with a median of 4.66 min and a mean of 5.05 min. To adopt a conservative approach, we fixed *T* at 4 min. We then used baseline FC estimated from 4 min of fMRI data to predict Year 2 cognition (Fig. 5a and b).

### Using longitudinal FC change (delta) to predict longitudinal cognitive change

To investigate the divergence and convergence of longitudinal and cross-sectional estimates of brain–cognition relationship, we also used longitudinal FC change to predict the longitudinal cognitive change. We controlled for sex, baseline age, age interval, baseline head motion (mean FD) and Year 2 head motion (mean FD). All regressions were performed on the training set, and the resulting regression coefficients were applied to the test set.

For comparison, we also used baseline FC to predict the cognitive change. Here, we controlled for sex, baseline age, age interval, and baseline head motion (mean FD). Once again, all regressions were performed on the training set, and the resulting regression coefficients were applied to the test set.

As a control analysis, we repeated the analyses using the rate of FC change instead of FC change and rate of cognitive change instead of cognitive change (Fig. S16). We controlled for sex, baseline age, age interval, baseline head motion (mean FD) and Year 2 head motion (mean FD). All regressions were performed on the training set, and the resulting regression coefficients were applied to the test set.

### Sex-stratified and sex-difference analyses

To assess sex-specific effects, all analyses were additionally repeated separately in females and males. Statistical tests were performed to compare the sexes. Detailed descriptions of sex-stratified and sex-comparison procedures are provided in the Supplementary Methods.

### Replication in a more representative subsample

To ensure our results are not due to a non-representative sample, we generated a subset of participants (*N* = 2020) from the main analytic cohort (*N* = 2949) such that its joint distribution of key demographic variables (age, sex, household income, and race/ethnicity) and baseline cognition approximated that of the full ABCD baseline cohort. All matching variables were discretized into predefined bins, with two bins for age and sex, five bins for race/ethnicity, four bins for household income, and three bins for baseline cognition (PC1). Participants in the analytic cohort were assigned to multi-dimensional bins defined by the cross-classification of these variables, and target counts for each bin were set to approximate their proportions in the full ABCD baseline cohort, subject to availability constraints in the analytic sample. A fixed-size subsample was then selected to match these target bin counts.

### Statistical tests of prediction accuracy

To test whether prediction accuracy was better than chance, permutation tests were performed by shuffling cognitive measures across participants 1000 times within sites and then repeating the leave-3-site-cluster-out cross-validation procedure.

To compare prediction accuracies between models, we employed the corrected resampled t-test^88^, which corrected for dependencies across cross-validation folds.

All *p*-values were computed based on two tails. Multiple comparisons were controlled using the false discovery rate (FDR)^89^ with *q* < 0.05.

### Statistical tests with spatial permutation

To assess the significance of correlations between ICCs from 4-min baseline FC and 20-min FC change, as well as between PNFs derived from baseline and longitudinal models, we used a spatial permutation (“spin”) test^90,91^. A total of 400 cortical parcels^31^ were randomly rotated on the spherical surface 1000 times to generate a null distribution while preserving spatial autocorrelation. For each permutation, the rows and columns of the matrix were reordered according to the rotated parcellation labels, and a null correlation was computed between the permuted matrix and the original comparison matrix. The observed correlation was then compared against this null distribution to determine significance.

### Reporting summary

Further information on research design is available in the Nature Portfolio Reporting Summary linked to this article.

## Supplementary information

Supplementary Information

Reporting Summary

Transparent Peer Review file

## Source data

Source Data

## Data Availability

The ABCD data are publicly available through the NIH Brain Development Cohorts (NBDC) Data Hub. Researchers with access to the ABCD data will be able to download the data from https://nbdc-datashare.lassoinformatics.com. Source data supporting the findings of this study are provided with this paper. Source data are provided with this paper.

## Code availability

Image preprocessing was conducted using previously published pipelines available at https://github.com/ThomasYeoLab/Standalone_CBIG_fMRI_Preproc2016, archived at 10.5281/zenodo.19723245^92^. Study-specific preprocessing scripts are available at https://github.com/ThomasYeoLab/ABCD_scripts, archived at 10.5281/zenodo.19723255^93^. Analysis code is publicly available at https://github.com/ThomasYeoLab/Standalone_Xie2025_LBC and archived at 10.5281/zenodo.19219794^94^.
