# Supplementary material for: Convergent and divergent brain–cognition development in early adolescence
Source: Nat Commun. 2026 May 26;17:6868. doi: 10.1038/s41467-026-73668-y (PMC13388698; doi:10.1038/s41467-026-73668-y)
Supplement: Supplementary file 1 — Supplementary Information [file 41467_2026_73668_MOESM1_ESM.pdf]

Convergent and divergent brain–cognition development in early adolescence

Supplementary information

Contents

Supplementary Methods

S1. Sex-stratified and sex difference analyses

S2. Relationships between head motion and age

S3. Relationships between head motion and cognition

S4. Mathematical relationship between the reliability of FC change and reliability of cross-sectional FC

Supplementary Figures

Figure S1 Illustration of how cross-sectional and longitudinal analyses can lead to divergent estimates

Figure S2 Cognitive stability for **females** and **males**

Figure S3 FC stability and longitudinal change in **females** and **males**

Figure S4 Enhanced FC-cognition relationship during development

Figure S5 Within-network versus between-network predictive network feature (PNF) contributions for each model

Figure S6 Enhanced FC-cognition relationship during development in **females**

Figure S7 Same as Figure S6, but for **males**

Figure S8. Models trained on baseline FC to predict baseline cognition improve in accuracy when applied to Year 2 FC and Year 2 cognition

Figure S9. Models trained on baseline FC to predict baseline cognition improve in accuracy when applied to Year 2 FC and Year 2 cognition, even after **controlling for head motion**

Figure S10. Models trained on baseline FC to predict baseline cognition improve in accuracy when applied to Year 2 FC and Year 2 cognition in **females**

Figure S11. Same as S10, but for **males**

Figure S12. Baseline FC is more predictive of cognition at Year 2 than longitudinal FC change, even accounting for reliability differences.

Figure S13. Baseline FC is more predictive of cognition at Year 2 than **rate of FC change**

Figure S14. Baseline FC is more predictive of cognition at Year 2 than longitudinal FC change in **females**, even accounting for reliability differences

Figure S15. Same as Figure 14, but for **males**

Figure S16. Limited prediction of rate of cognitive change from baseline FC and rate of FC change

Figure S17. Limited prediction of cognitive change from baseline FC and longitudinal FC change in **females**

Figure S18. Same as Figure 17, but in **males**

Figure S19. Convergent and divergent predictive network features (PNFs) between cross-sectional and longitudinal estimates of FC–cognition relationship within each sex

Figure S20. Convergent and divergent predictive network features (PNFs) between cross-sectional and longitudinal estimates of FC-cognition relationships for the eight cognitive measures

Figure S21. Individual differences in longitudinal cognitive change during the transition from childhood to adolescence in a representative subsample (N = 2020)

Figure S22. Individual differences in longitudinal functional connectivity (FC) change in a representative subsample (N = 2020)

Figure S23. Enhanced FC–cognition relationships during development in a representative subsample (N = 2020)

Figure S24. Models trained on baseline FC to predict baseline cognition improve in accuracy when applied to Year 2 FC and Year 2 cognition in a representative subsample (N = 2020)

Figure S25. Baseline FC is more predictive of cognition at Year 2 than longitudinal FC change, even accounting for reliability differences in a representative subsample (N = 2020)

Figure S26. Limited prediction of cognitive change from baseline FC and longitudinal FC change in a representative subsample (N = 2020)

Figure S27. Convergent and divergent predictive network features (PNFs) between cross-sectional and longitudinal estimates of FC–cognition relationship in a representative subsample (N = 2020)

Supplementary Tables

Table S1. Linear mixed-effects model results for females, males and sex differences in cognitive change.

Table S2. Linear mixed-effects model results for relationship between motion and cognition

Table S3. Prediction accuracy for each cognitive measure and each cross-sectional model

Table S4. Compare distribution of included and excluded participants after matching

Supplementary References

Supplementary Methods

S1. Sex-stratified and sex difference analyses

Longitudinal cognitive stability. Based on the harmonized cognitive scores obtained after longitudinal ComBat (as described in the main Methods), analyses were conducted separately in females and males. For each sex, baseline age was regressed from the harmonized baseline cognitive scores, and Year 2 age was regressed from the harmonized Year 2 cognitive scores. Sex was not included as a covariate in these models, as analyses were performed within sex. Longitudinal cognitive stability was then assessed by computing Spearman's correlations between the resulting residuals.

To compare sex difference in cognitive stability, we compared the correlation coefficients between baseline and Year 2 cognitive scores across females and males for each cognitive measure. Specifically, correlation coefficients were then transformed to Fisher's z values using the Fisher *r-to-z* transformation. The difference between the two Fisher-transformed correlations was evaluated using a z test, with the standard error calculated as $\sqrt{\frac{1}{n_{female}-3}+\frac{1}{n_{male}-3}}$, where n_{female} and n_{male} denote the sample sizes for females and males, respectively. Two tailed p values were derived from the standard normal distribution. Multiple comparisons were controlled using the false discovery rate (FDR) [1] with $q < 0.05$.

Longitudinal cognitive change. To examine longitudinal change in cognitive performance and potential sex differences, we fitted linear mixed-effects models including an interaction between sex and age interval. Analyses were conducted on cognitive scores harmonized using longitudinal ComBat. Specifically, baseline age (Age_{i1}), age interval between baseline and Year 2 ($\text{Age}_{it} - \text{Age}_{i1}$), sex, and the sex × age-interval interaction were included as fixed effects. Sex was coded as a binary variable (male = 0, female =1). In addition, subject-specific random intercepts and random slopes for age interval were modeled to capture individual differences in baseline cognition and rates of longitudinal change:

$$\begin{aligned}\text{Cognition}_{it} \;=\; &\beta_0 + \beta_1\text{Age}_{i1} + \beta_2(\text{Age}_{it} - \text{Age}_{i1}) + \beta_3\text{Sex}_i + \beta_4(\text{Age}_{it} - \text{Age}_{i1}) \times \text{Sex}_i \\ &+ b_i + c_i\,(\text{Age}_{it} - \text{Age}_{i1}) + \varepsilon_{it}\,, \qquad (1)\end{aligned}$$

Where i indexes the i-th participant, t references the time point, Cognition_{it} is the cognition of the i-th participant at timepoint t after longitudinal ComBat. The term b_i and c_i represent participant-specific random intercepts and random slope, respectively.

However, we note that Equation 1 can only capture a group-level estimate of longitudinal change. Therefore, to capture the potential individual differences in longitudinal cognition change, for each participant and each cognitive score, we computed the longitudinal cognition change as: Cognition_{i2} - Cognition_{i1}, where Cognition_{it} is the cognition of the i-th participant at timepoint t (from longitudinal ComBat). We then regressed out baseline age and age interval (i.e., between Year 2 and baseline) from the individual-level longitudinal cognition change estimate.

We also examined sex differences in the variability of longitudinal cognitive change. Specifically, residualized longitudinal cognitive change values from females and males were pooled and grouped by sex, and the Brown–Forsythe test was applied to assess differences in variance between the two groups. In addition, the standard deviation (SD) of longitudinal cognitive change was computed separately for females and males for each cognitive measure, and the ratio of SDs (female/male) was calculated to summarize the magnitude and direction of sex differences in variability, with males used as the reference group. Two-tailed p values from the Brown–Forsythe tests were retained for each cognitive measure.

Longitudinal FC stability. Based on functional connectivity (FC) measures harmonized using longitudinal ComBat (as described in the main Methods), analyses were performed separately in females and males. Within each sex, baseline age and mean framewise displacement (FD) were regressed from the harmonized baseline FC, and Year-2 age and mean FD were regressed from the harmonized Year-2 FC. Sex was not included as a covariate in these models, as all analyses were conducted within sex. Longitudinal FC stability was then quantified by computing Spearman's correlations between the resulting residuals.

Sex differences in FC stability were assessed following the same procedure used for cognitive stability, by comparing Fisher z–transformed Spearman correlation coefficients between females and males.

Longitudinal FC change. To examine longitudinal change in FC and potential sex differences, we fitted linear mixed-effects models including an interaction between sex and age interval. Analyses were conducted on FC harmonized using longitudinal ComBat. Specifically, baseline age (Age_{i1}), age interval between baseline and Year 2 ($\text{Age}_{it} - \text{Age}_{i1}$), sex, sex × age-interval interaction and mean FD were included as fixed effects. In addition, subject-specific random intercepts and random slopes for age interval were modeled to capture individual differences in baseline cognition and rates of longitudinal change:

$$\text{FC}_{it} = \beta_0 + \beta_1\text{Age}_{i1} + \beta_2(\text{Age}_{it} - \text{Age}_{i1}) + \beta_3\text{Sex}_i + \beta_4(\text{Age}_{it} - \text{Age}_{i1}) \times \text{Sex}_i + \beta_5\text{meanFD}_{it} + b_i + c_i(\text{Age}_{it} - \text{Age}_{i1}) + \varepsilon_{it}\,, \quad (2)$$

where *i* indexes the *i*-th participant, *t* references the time point. FC_{it} is the FC value of a particular FC edge of the *i*-th participant at timepoint *t* after longitudinal ComBat. The term b_i and c_i represent participant-specific random intercepts and random slope, respectively.

However, we note that Equation 2 captures only the group-level estimate of longitudinal change. Therefore, to capture the potential individual differences in longitudinal FC change, for each participant and each FC edge, we computed the z value of FC change across the two timepoints:

$$z = \frac{r_2 - r_0}{\sqrt{s_2 + s_0}}\,, \quad (3)$$

where r_2 and r_0 denote Fisher r-to-z transformed FC value for the FC edge at Year 2 and baseline respectively. s_2 and s_0 denote the variance of the FC value for each FC edge at Year 2 and baseline respectively. The estimated variance needed to account for auto-correlation in the fMRI time series, so we used the MATLAB function xDF.m (https://github.com/asoroosh/xDF) to estimate r and s for each FC edge at each time point [2]. We then regressed, baseline age and age

interval (between Year 2 and baseline), mean head motion (FD) at baseline, mean head motion (FD) at Year 2 from the z-statistic across participants.

Sex differences in the variability of longitudinal FC change were assessed following the same procedure used for longitudinal cognitive change.

Prediction analysis. All prediction analyses were repeated separately in females and males following the same procedure as the pooled analysis, with sex omitted as a covariate. We also compared sex differences in prediction accuracy for each model using corrected resampled t tests. Specifically, for each model, prediction accuracies were obtained across 120 cross-validation folds separately for females and males, and sex differences in accuracy were evaluated by comparing fold-wise accuracies across the two groups while accounting for dependencies across folds.

S2. Relationships between head motion and age

To evaluate motion changes from baseline to Year 2, we applied the following linear mixed-effects model to estimate the effect of within-participant age change, accounting for baseline age, sex, site, and participant-specific random intercepts:

$$\text{Motion}_{it} = \beta_0 + \beta_1 \text{Age}_{i1} + \beta_2(\text{Age}_{it} - \text{Age}_{i1}) + \beta_3 \text{Sex}_{it} + \beta_4 \text{SiteID}_{it} + u_i + \varepsilon_{it}, \quad (4)$$

where i indexes the i-th participant, t references the time point. u_i is the random intercept and ε_{it} is the noise term. Mean framewise displacement (FD) and std FD are the dependent variables.

S3. Relationships between head motion and cognition

To assess whether motion is systematically related to the cognitive measures of interest, we fit an additional linear mixed effect model:

$$\text{Motion}_{it} = \beta_0 + \beta_1 \text{Age}_{i1} + \beta_2(\text{Age}_{it} - \text{Age}_{i1}) + \beta_3 \text{Sex}_{it} + \beta_4 \text{Cognition}_{it} + \beta_5 \text{SiteID}_{it} + u_i + \varepsilon_{it}, \quad (5)$$

where i indexes the i-th participant, t references the time point. u_i is the random intercept and ε_{it} is the noise term. Mean framewise displacement (FD) and std FD are the dependent variables. We applied this model to all the eight cognitive measures.

S4. Mathematical relationship between the reliability of FC change and the reliability of cross-sectional FC

Here we provide a detailed derivation linking the reliability of FC change to the reliability of FC at two timepoints[3].

Denote an FC edge at time point 1 as FC_1 and that same FC edge at time point 2 as FC_2. We can decompose FC_1 and FC_2 into 2 components:

$$\text{FC}_1 = T_1 + \epsilon_1$$
$$\text{FC}_2 = T_2 + \epsilon_2$$

where T_1 and T_2 are the true noise-free components of the FCs and ϵ are noises.

Define the reliability of FC_1 as $R_1 = \frac{V(T_1)}{V_1}$. Similarly, $R_2 = \frac{V(T_2)}{V_2}$, where $V(T_i)$ is the variance of T_i and V_i is the variance of FC_i.

In general, for a given random variable X, we have $V_X = V(T_X) + V(\epsilon_X)$. Therefore,

$$\begin{aligned} V(\epsilon_X) &= V_X - V(T_X) \\ &= V_X - R_X V_X \\ &= (1 - R_X)V_X \end{aligned}$$

More specifically, $V(\epsilon_1) = (1 - R_1)V_1$ and $V(\epsilon_2) = (1 - R_2)V_2$.

Our question is to relate the reliability of $\text{FC}_D = \text{FC}_1 - \text{FC}_2$ (denoted as R_D) with the reliability of cross-sectional measures FC_1 and FC_2.

First,

$$\text{FC}_D = \text{FC}_1 - \text{FC}_2 = T_1 - T_2 + \epsilon_1 - \epsilon_2$$

Then,

$$R_D = \frac{V(T_D)}{V_D}$$

$$= \frac{V(T_1) + V(T_2) - 2\text{Cov}(T_1, T_2)}{V(T_1) + V(T_2) - 2\text{Cov}(T_1, T_2) + V(\epsilon_1) + V(\epsilon_2) - \cancel{2\text{Cov}(\epsilon_1, \epsilon_2)}}$$

$$= \frac{V(T_1) + V(T_2) - 2\rho_T\sqrt{V(T_1)V(T_2)}}{V(T_1) + V(T_2) - 2\rho_T\sqrt{V(T_1)V(T_2)} + V(\epsilon_1) + V(\epsilon_2)}$$

$$= \frac{R_1V_1 + R_2V_2 - 2\rho_T\sqrt{R_1V_1R_2V_2}}{R_1V_1 + R_2V_2 - 2\rho_T\sqrt{R_1V_1R_2V_2} + (1 - R_1)V_1 + (1 - R_2)V_2}$$

$$= \frac{R_1V_1 + R_2V_2 - 2\rho_T\sqrt{R_1V_1R_2V_2}}{V_1 + V_2 - 2\rho_T\sqrt{R_1V_1R_2V_2}} \qquad (6)$$

where we have assumed $\text{Cov}(\epsilon_1, \epsilon_2) = 0$ (i.e., measurement noise at the two timepoints are independent) and ρ_T is the true correlation between T_1 and T_2. Define ρ as the observed correlation between FC_1 and FC_2. The observed correlation will be attenuated by the reliability of FC_1 and FC_2, so $\rho = \rho_T\sqrt{R_1R_2}$ (which is the standard attenuation formula). Equation (1) then becomes

$$R_D = \frac{R_1V_1 + R_2V_2 - 2\rho\sqrt{V_1V_2}}{V_1 + V_2 - 2\rho\sqrt{V_1V_2}} \qquad (7)$$

Supplementary Figures

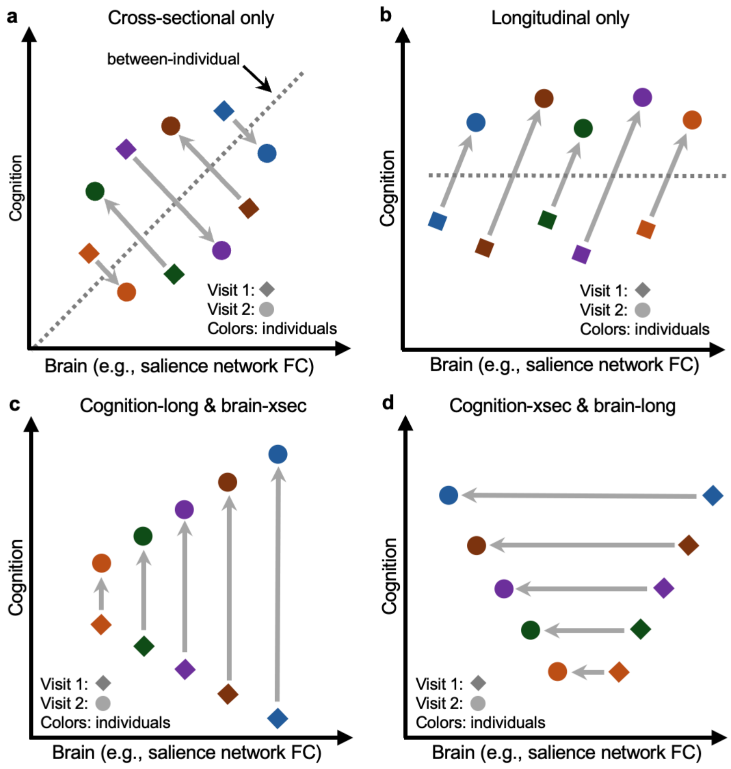

Figure S1. Illustration of how cross-sectional and longitudinal analyses can lead to divergent estimates. Colours represent individual participants; diamond and circular symbols indicate baseline and Year 2, respectively. (a) A cross-sectional brain–cognition relationship exists, but not longitudinal one. Individual-averaged brain measures are associated with individual-averaged cognitive measures, but within-individual values are not associated. (b) A longitudinal brain–cognition relationship exists, but not cross-sectional one. Within-individual change in brain measures and cognitive measures are associated, but individual-averaged values are not associated. (c) Cross-sectional brain measures are associated with longitudinal cognitive change. Individual-averaged brain measures are associated with within-individual changes in cognition, but within-individual brain measures are not informative. (d) Longitudinal brain measures are associated with individual-averaged cognitive measures. Within-individual changes in brain measures are associated with individual-averaged cognition measures, but individual-averaged brain measures are not informative.

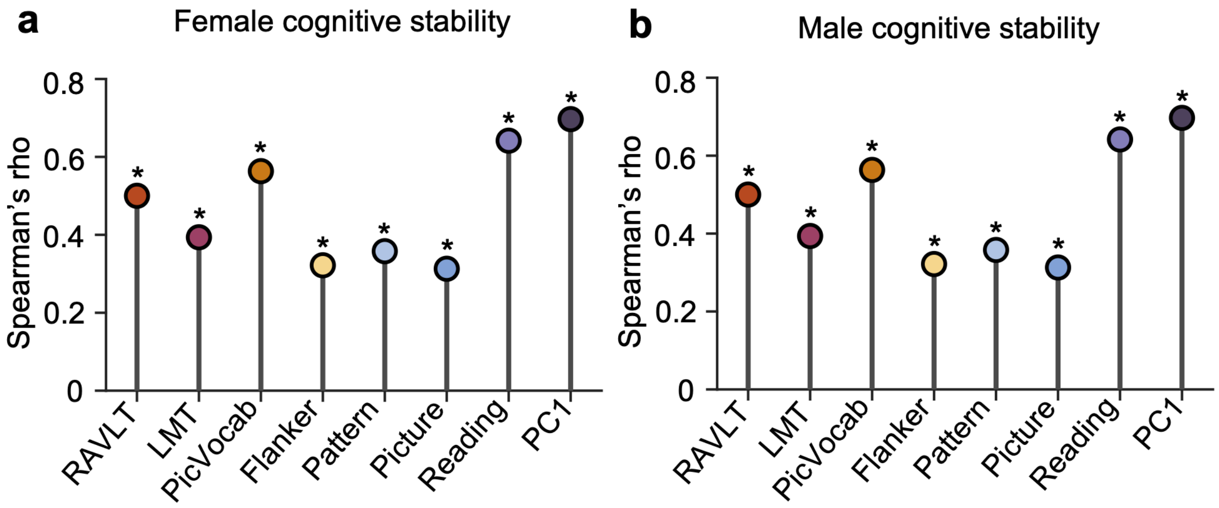

Figure S2. Cognitive stability for **females** and **males**. (a) Spearman's correlation (stability) between baseline and Year 2 cognitive measures in females. Each marker represents a cognitive measure. Positive correlations indicate that children with higher baseline cognition generally maintained their cognitive advantage over their peers at Year 2. (b) Spearman's correlation (stability) between baseline and Year 2 cognitive measures in males. Visualization elements are as in (a). RAVLT: Rey Auditory Verbal Learning Test (verbal memory); LMT: Little Man Task (spatial reasoning); PicVocab: Picture Vocabulary Task (vocabulary); Flanker: Flanker Task (executive function); Pattern: Pattern Comparison Processing Speed Test (processing speed); Picture: Picture Sequence Memory Test (episodic memory); Reading: Oral Reading Recognition Task (reading ability). PC1: the first principal component of the above seven cognitive measures. Asterisks (*) indicate statistical significance after false discovery rate (FDR) correction [1] at $q < 0.05$.

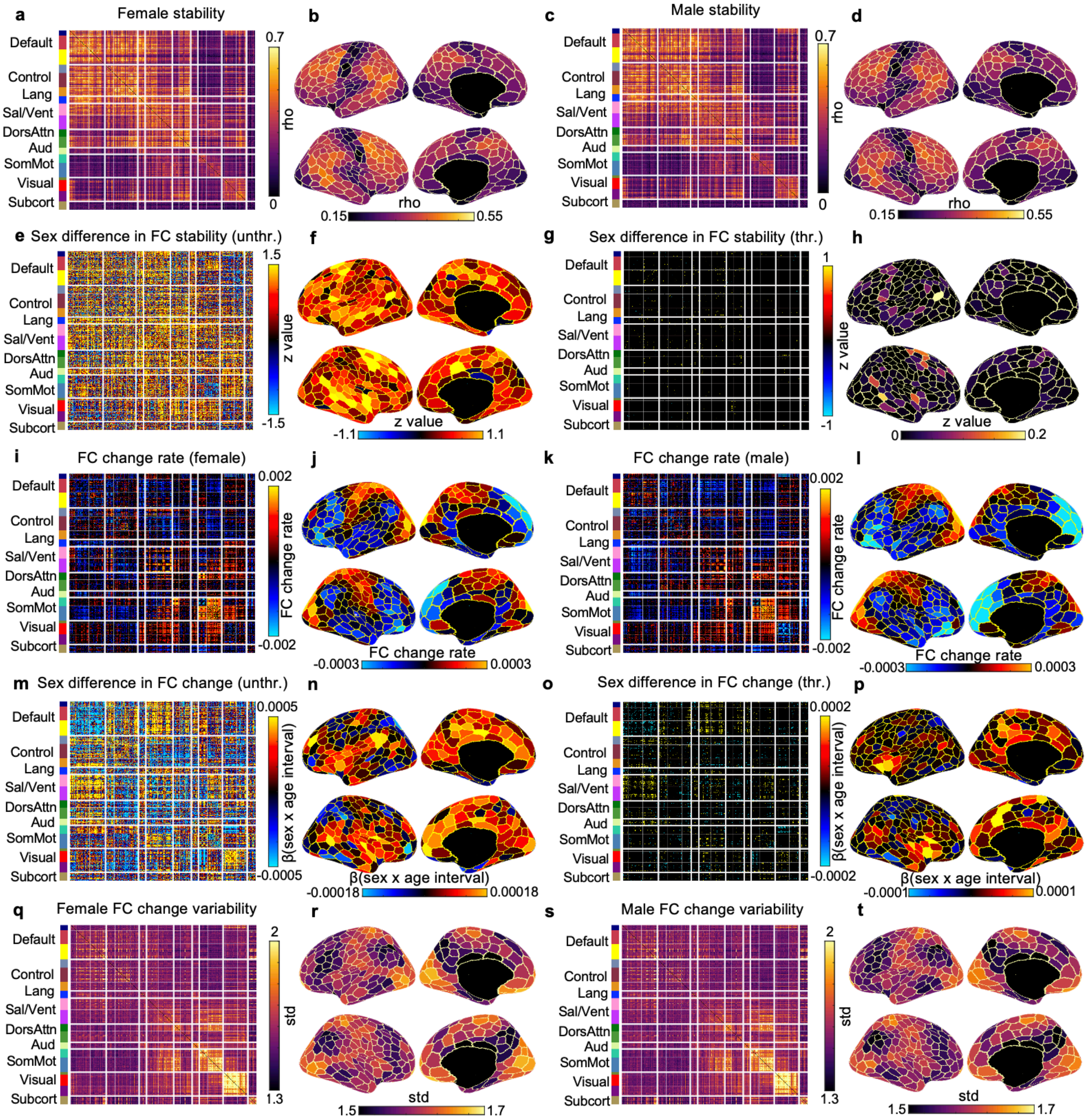

Figure S3. FC stability and longitudinal change in **females** and **males**. (a) Spearman's correlation (stability) between baseline (FCY0) and Year 2 FC (FCY2) for each FC edge in females. Positive correlations (rho; warmer colours) indicate that children exhibiting stronger brain connectivity at baseline continued to exhibit stronger brain connectivity than their peers at Year 2. (b) Regional visualization of female FC stability, by averaging the rows of panel a. (c) Spearman's correlation (stability) between baseline (FCY0) and Year 2 FC (FCY2) for each FC edge in males. (d) Regional visualization of male FC stability, by averaging the rows of panel c. (e) Unthresholded sex differences in FC stability (female – male). Positive (z value; warmer colours) implies greater stability in females. (f) Regional visualization of unthresholded sex differences in FC stability, by averaging the rows of panel e. (g) Thresholded sex differences in FC stability. At the edge level, sex differences were sparse, with only 0.36% of connections reaching statistical significance, after correcting for multiple comparisons with FDR q < 0.05. (h) Regional visualization of thresholded

sex differences in FC stability, by averaging the rows of panel g. (i) Longitudinal FC change rate in females at the group level based on a linear mixed effects model. Values represent the estimated slope (β) of FC change over time. (j) Regional visualization of female longitudinal change rate, by averaging the rows of panel i. (k) Longitudinal FC change rate in males at the group level based on the linear mixed effects model. Values represent the estimated slope (β) of FC change over time. (l) Regional visualization of male longitudinal change rate, by averaging the rows of panel k. (m) Unthresholded sex differences in FC change (female – male). Positive indicates greater change in females. (n) Regional visualization of unthresholded sex differences in FC change, by averaging the rows of panel m. (o) Thresholded sex differences in FC change, quantified by the coefficient (β) of the sex × age interval term (female − male slope). At the edge level, only 4% connections reaching statistical significance in the linear mixed-effects model, after correcting for multiple comparisons with FDR $q < 0.05$. (p) Regional visualization of thresholded sex differences in FC change, by averaging the rows of panel (o). (q) Female variability in longitudinal FC change. FC change (z value) was computed for each FC edge[2]. Baseline age, age interval (between baseline and Year 2), and head motion at two timepoints were regressed out. Standard deviation was then computed across individuals. (r) Regional visualization of female variability in longitudinal FC change, by averaging the rows of panel q. (s) Male variability in longitudinal FC change. (t) Regional visualization of male variability in longitudinal FC change, by averaging the rows of panel s. FC change rates correspond to the fixed effects of the age-interval term ($\mathbf{Age}_{it} - \mathbf{Age}_{i1}$) in the mixed-effects model (β_2 for males; $\beta_2 + \beta_4$ for females); see Equation 2 in Supplementary Methods S1.

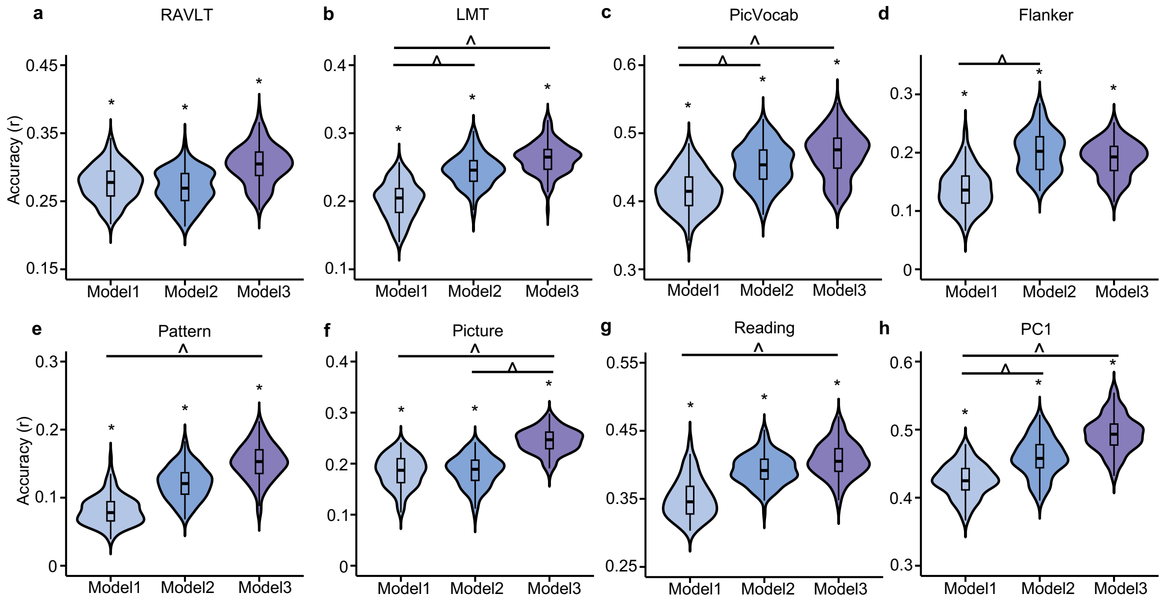

Figure S4. Enhanced FC-cognition relationship during development. (a-h) Comparison of prediction accuracies across the three models for the eight cognitive measures. Model 1 predicts baseline cognition using baseline FC (FCY0 → CogY0, light blue). Model 2 predicts Year 2 cognition using baseline FC (FCY0 → CogY2, blue). Model 3 predicts Year 2 cognition using Year 2 FC (FCY2 → CogY2, purple). Each value in the violin plot represents accuracy (r) for a single cross-validation fold. Inset box plots indicate the median (center line), interquartile range (25th–75th percentile; box), and whiskers extending to 1.5 × the interquartile range, with outliers not shown. Asterisks (*) denote above chance prediction after multiple comparisons correction (FDR $q < 0.05$). Carets (^) denote statistically significant differences between models based on the corrected resampled t-test (FDR $q < 0.05$). Note: y-axes differ across panels to enhance visibility and emphasize the performance of models within each task. Comparisons across tasks should be made with caution due to varying scales. RAVLT: Rey Auditory Verbal Learning Test (verbal memory); LMT: Little Man Task (spatial reasoning); PicVocab: Picture Vocabulary Task (vocabulary); Flanker: Flanker Task (executive function); Pattern: Pattern Comparison Processing Speed Test (processing speed); Picture: Picture Sequence Memory Test (episodic memory); Reading: Oral Reading Recognition Task (reading ability). PC1: the first principal component of the above seven cognitive measures. Separate results for males and females can be found in Figures S6 and S7.

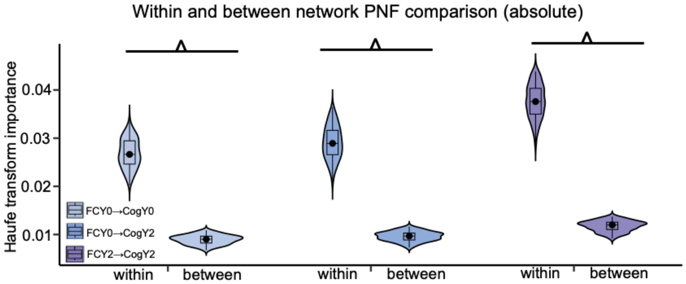

Figure S5. Within-network versus between-network predictive network feature (PNF) contributions for each model. Model 1 predicts baseline cognition using baseline FC (FCY0 → CogY0, light blue). Model 2 predicts Year 2 cognition using baseline FC (FCY0 → CogY2, blue). Model 3 predicts Year 2 cognition using Year 2 FC (FCY2 → CogY2, purple). For each cross-validation fold, mean within-network and between-network PNF magnitudes (absolute values) were computed separately for each model. Each violin plot contains 120 data points, corresponding to the 120 repetitions of cross-validation. Inset box plots indicate the median (center line), interquartile range (25th–75th percentile; box), and whiskers extending to 1.5 × the interquartile range, with outliers not shown. Carets (^) indicate statistically significant within-network versus between-network differences based on the corrected resampled *t*-test (FDR $q < 0.05$).

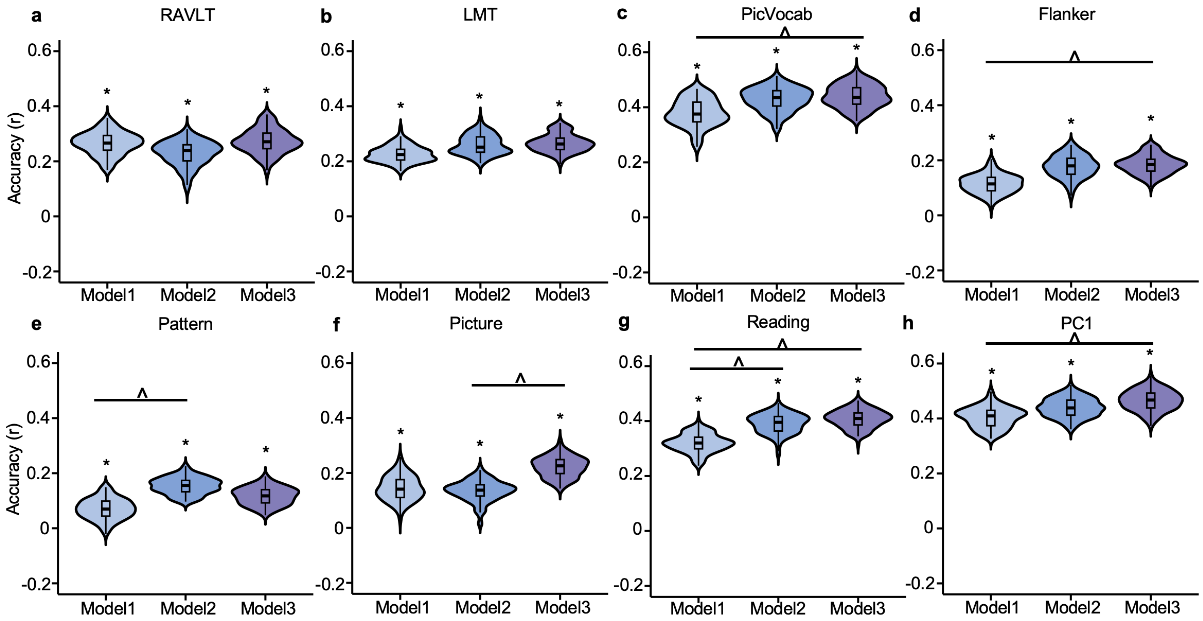

Figure S6. Enhanced FC-cognition relationship during development in **females**. (a-h) Comparison of prediction accuracies across the three models for the eight cognitive measures in females. Model 1 predicts baseline cognition using baseline FC (FCY0 → CogY0, light blue). Model 2 predicts Year 2 cognition using baseline FC (FCY0 → CogY2, blue). Model 3 predicts Year 2 cognition using Year 2 FC (FCY2 → CogY2, purple). Each value in the violin plot represents the accuracy (r) for a single cross-validation fold. Inset box plots indicate the median (center line), interquartile range (25th–75th percentile; box), and whiskers extending to 1.5 × the interquartile range, with outliers not shown. Asterisks (*) denote above chance prediction after multiple comparisons correction (FDR $q < 0.05$). Carets (^) denote statistically significant differences between models based on the corrected resampled t-test (FDR $q < 0.05$). RAVLT: Rey Auditory Verbal Learning Test (verbal memory); LMT: Little Man Task (spatial reasoning); PicVocab: Picture Vocabulary Task (vocabulary); Flanker: Flanker Task (executive function); Pattern: Pattern Comparison Processing Speed Test (processing speed); Picture: Picture Sequence Memory Test (episodic memory); Reading: Oral Reading Recognition Task (reading ability). PC1: the first principal component of the above seven cognitive measures.

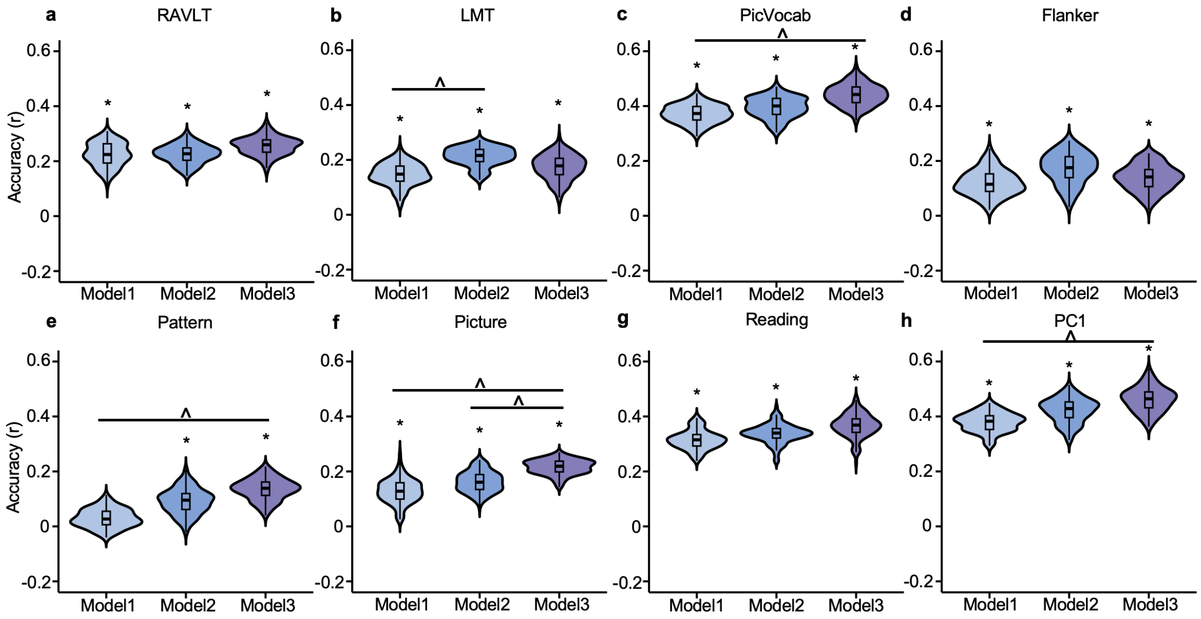

Figure S7. Enhanced FC-cognition relationship during development in **males**. (a-h) Comparison of prediction accuracies across the three models for the eight cognitive measures in males. Model 1 predicts baseline cognition using baseline FC (FCY0 → CogY0, light blue). Model 2 predicts Year 2 cognition using baseline FC (FCY0 → CogY2, blue). Model 3 predicts Year 2 cognition using Year 2 FC (FCY2 → CogY2, purple). Each value in the violin plot represents the accuracy (r) for a single cross-validation fold. Inset box plots indicate the median (center line), interquartile range (25th–75th percentile; box), and whiskers extending to 1.5 × the interquartile range, with outliers not shown. Asterisks (*) denote above chance prediction after multiple comparisons correction (FDR $q < 0.05$). Carets (^) denote statistically significant differences between models based on the corrected resampled t-test (FDR $q < 0.05$). RAVLT: Rey Auditory Verbal Learning Test (verbal memory); LMT: Little Man Task (spatial reasoning); PicVocab: Picture Vocabulary Task (vocabulary); Flanker: Flanker Task (executive function); Pattern: Pattern Comparison Processing Speed Test (processing speed); Picture: Picture Sequence Memory Test (episodic memory); Reading: Oral Reading Recognition Task (reading ability). PC1: the first principal component of the above seven cognitive measures.

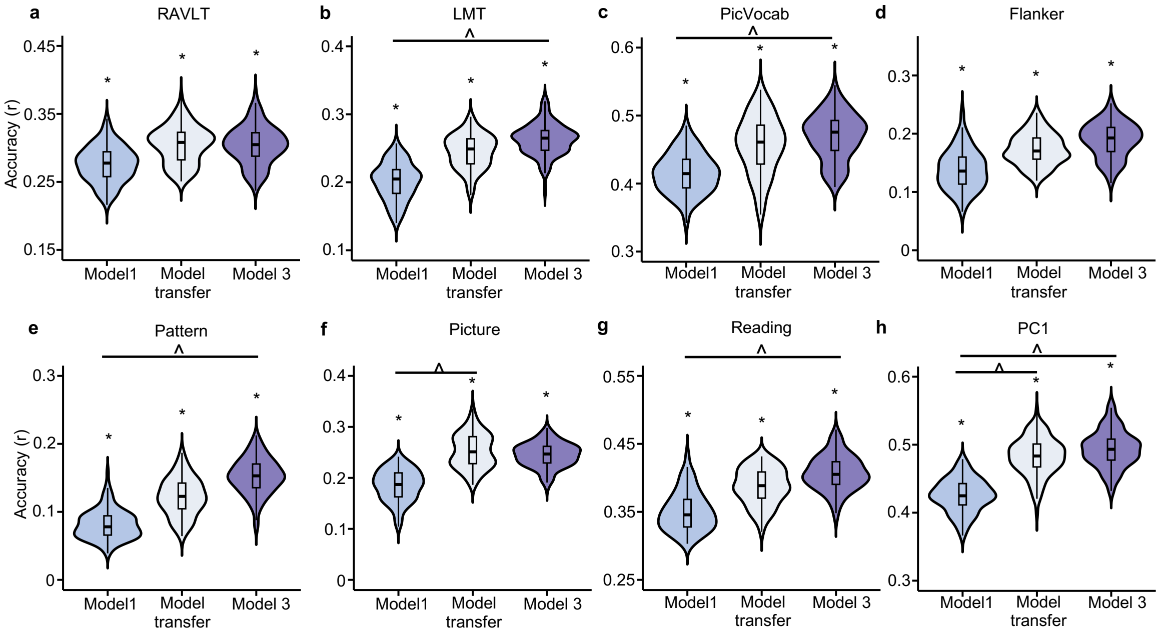

Figure S8. Models trained on baseline FC to predict baseline cognition improve in accuracy when applied to Year 2 FC and Year 2 cognition. (a–h) Each plot corresponds to a different cognitive measure. "Model 1" are the models obtained by training baseline FC to predict baseline cognition (FCY0 → CogY0, light blue). These models are then used to predict Y2 cognition from Year 2 FC, which we refer to as "Model transfer" (grey violin). Finally, for reference, "Model 3" are the models obtained by training Year 2 FC to predict Year 2 cognition (FCY2 → CogY2, purple). Each value in the violin plot represents the accuracy (r) for a single cross-validation fold. Inset box plots indicate the median (center line), interquartile range (25th–75th percentile; box), and whiskers extending to 1.5 × the interquartile range, with outliers not shown. Asterisks (*) denote above chance prediction after multiple comparisons correction (FDR $q < 0.05$). Carets (^) denote statistically significant differences between models based on the corrected resampled t-test (FDR $q < 0.05$). Note: y-axes differ across panels to enhance visibility and emphasize the performance of models within each task. Comparisons across tasks should be made with caution due to varying scales. RAVLT: Rey Auditory Verbal Learning Test (verbal memory); LMT: Little Man Task (spatial reasoning); PicVocab: Picture Vocabulary Task (vocabulary); Flanker: Flanker Task (executive function); Pattern: Pattern Comparison Processing Speed Test (processing speed); Picture: Picture Sequence Memory Test (episodic memory); Reading: Oral Reading Recognition Task (reading ability). PC1: the first principal component of the above seven cognitive measures.

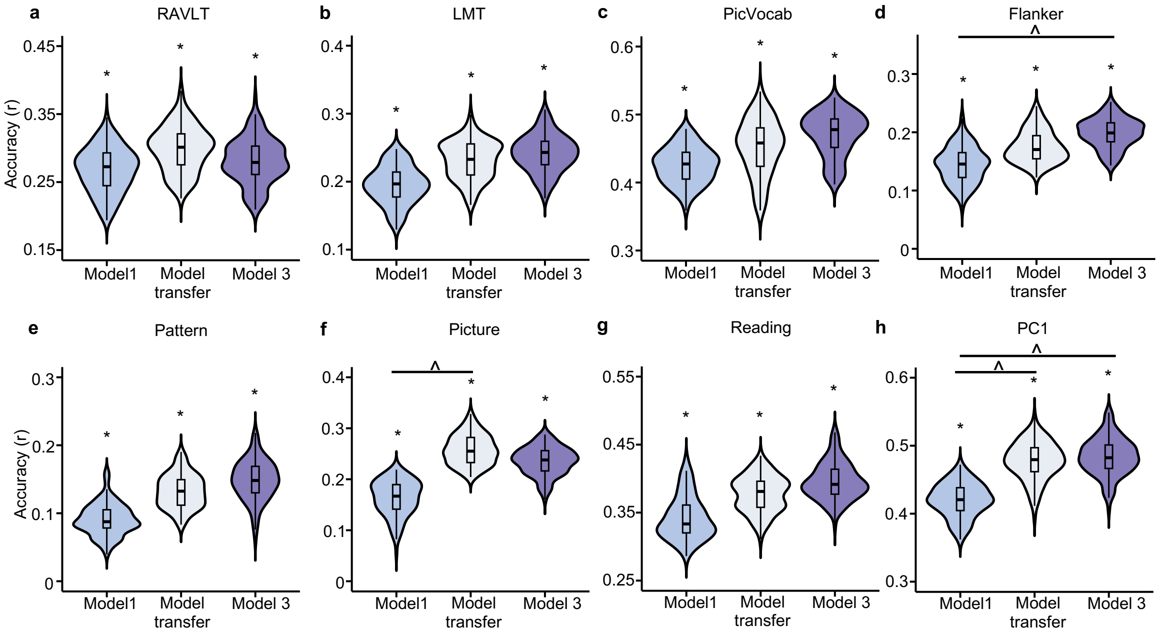

Figure S9. Models trained on baseline FC to predict baseline cognition improve in accuracy when applied to Year 2 FC and Year 2 cognition, even after **controlling for head motion**. Head motion was controlled by selecting a subset of participants (n = 2642) with no significant difference in mean frame-wise displacement across the two timepoints (p = 0.18). (a–h) Each plot corresponds to a different cognitive measure. "Model 1" are the models obtained by training baseline FC to predict baseline cognition (FCY0 → CogY0, light blue). These models are then used to predict Y2 cognition from Year 2 FC, which we refer to as "Model transfer" (grey violin). Finally, for reference, "Model 3" are the models obtained by training Year 2 FC to predict Year 2 cognition (FCY2 → CogY2, purple). Each value in the violin plot represents the accuracy (r) for a single cross-validation fold. Inset box plots indicate the median (center line), interquartile range (25th–75th percentile; box), and whiskers extending to 1.5 × the interquartile range, with outliers not shown. Asterisks (*) denote above chance prediction after multiple comparisons correction (FDR q < 0.05). Carets (^) denote statistically significant differences between models based on the corrected resampled t-test (FDR q < 0.05). Note: y-axes differ across panels to enhance visibility and emphasize the performance of models within each task. Comparisons across tasks should be made with caution due to varying scales. RAVLT: Rey Auditory Verbal Learning Test (verbal memory); LMT: Little Man Task (spatial reasoning); PicVocab: Picture Vocabulary Task (vocabulary); Flanker: Flanker Task (executive function); Pattern: Pattern Comparison Processing Speed Test (processing speed); Picture: Picture Sequence Memory Test (episodic memory); Reading: Oral Reading Recognition Task (reading ability). PC1: the first principal component of the above seven cognitive measures.

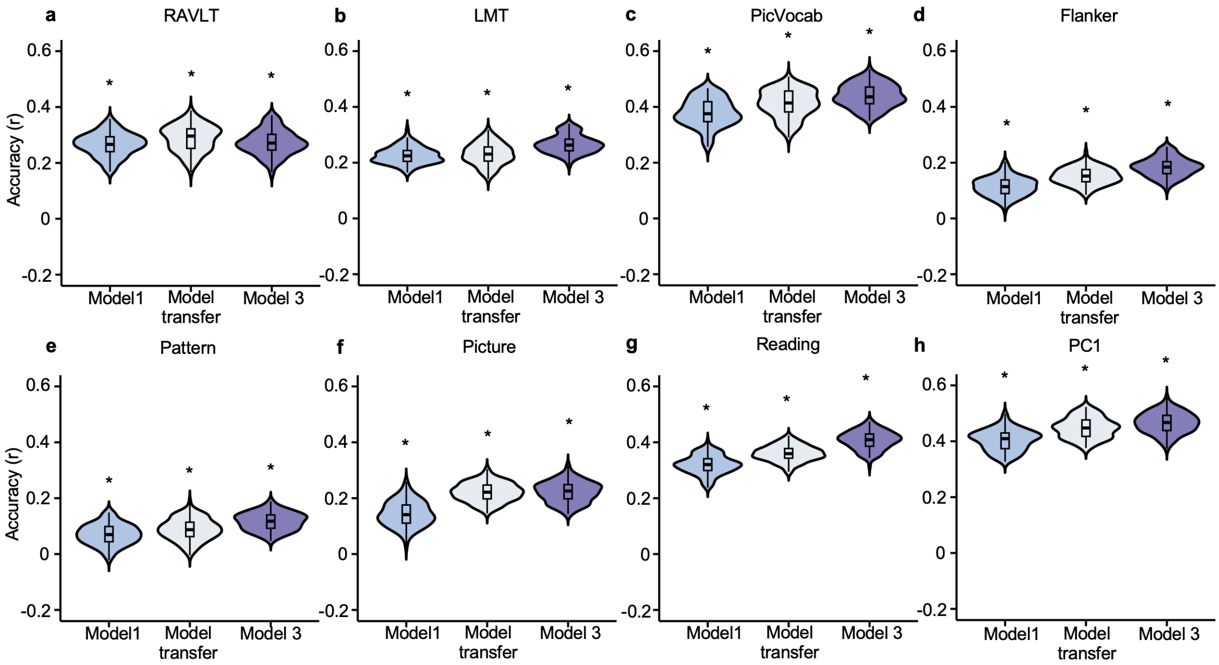

Figure S10. Models trained on baseline FC to predict baseline cognition improve in accuracy when applied to Year 2 FC and Year 2 cognition in **females**. (a–h) Each plot corresponds to a different cognitive measure. "Model 1" are the models obtained by training baseline FC to predict baseline cognition (FCY0 → CogY0, light blue). These models are then used to predict Y2 cognition from Year 2 FC, which we refer to as "Model transfer" (grey violin). Finally, for reference, "Model 3" are the models obtained by training Year 2 FC to predict Year 2 cognition (FCY2 → CogY2, purple). Each value in the violin plot represents the accuracy (r) for a single cross-validation fold. Inset box plots indicate the median (center line), interquartile range (25th–75th percentile; box), and whiskers extending to 1.5 × the interquartile range, with outliers not shown. Asterisks (*) denote above chance prediction after multiple comparisons correction (FDR $q < 0.05$). Carets (^) denote statistically significant differences between models based on the corrected resampled t-test (FDR $q < 0.05$). RAVLT: Rey Auditory Verbal Learning Test (verbal memory); LMT: Little Man Task (spatial reasoning); PicVocab: Picture Vocabulary Task (vocabulary); Flanker: Flanker Task (executive function); Pattern: Pattern Comparison Processing Speed Test (processing speed); Picture: Picture Sequence Memory Test (episodic memory); Reading: Oral Reading Recognition Task (reading ability). PC1: the first principal component of the above seven cognitive measures.

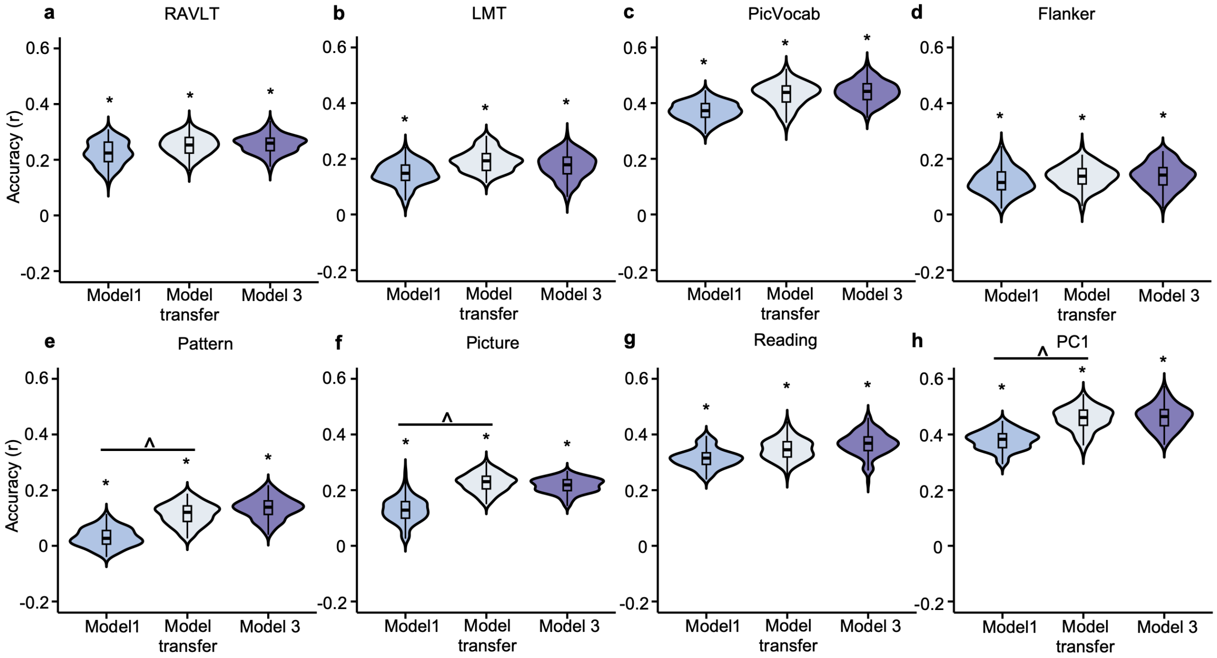

Figure S11. Models trained on baseline FC to predict baseline cognition improve in accuracy when applied to Year 2 FC and Year 2 cognition in **males**. (a–h) Each plot corresponds to a different cognitive measure. “Model 1” are the models obtained by training baseline FC to predict baseline cognition (FCY0 → CogY0, light blue). These models are then used to predict Y2 cognition from Year 2 FC, which we refer to as “Model transfer” (grey violin). Finally, for reference, “Model 3” are the models obtained by training Year 2 FC to predict Year 2 cognition (FCY2 → CogY2, purple). Each value in the violin plot represents the accuracy (r) for a single cross-validation fold. Inset box plots indicate the median (center line), interquartile range (25th–75th percentile; box), and whiskers extending to 1.5 × the interquartile range, with outliers not shown. Asterisks (*) denote above chance prediction after multiple comparisons correction (FDR $q < 0.05$). Carets (^) denote statistically significant differences between models based on the corrected resampled t-test (FDR $q < 0.05$). RAVLT: Rey Auditory Verbal Learning Test (verbal memory); LMT: Little Man Task (spatial reasoning); PicVocab: Picture Vocabulary Task (vocabulary); Flanker: Flanker Task (executive function); Pattern: Pattern Comparison Processing Speed Test (processing speed); Picture: Picture Sequence Memory Test (episodic memory); Reading: Oral Reading Recognition Task (reading ability). PC1: the first principal component of the above seven cognitive measures.

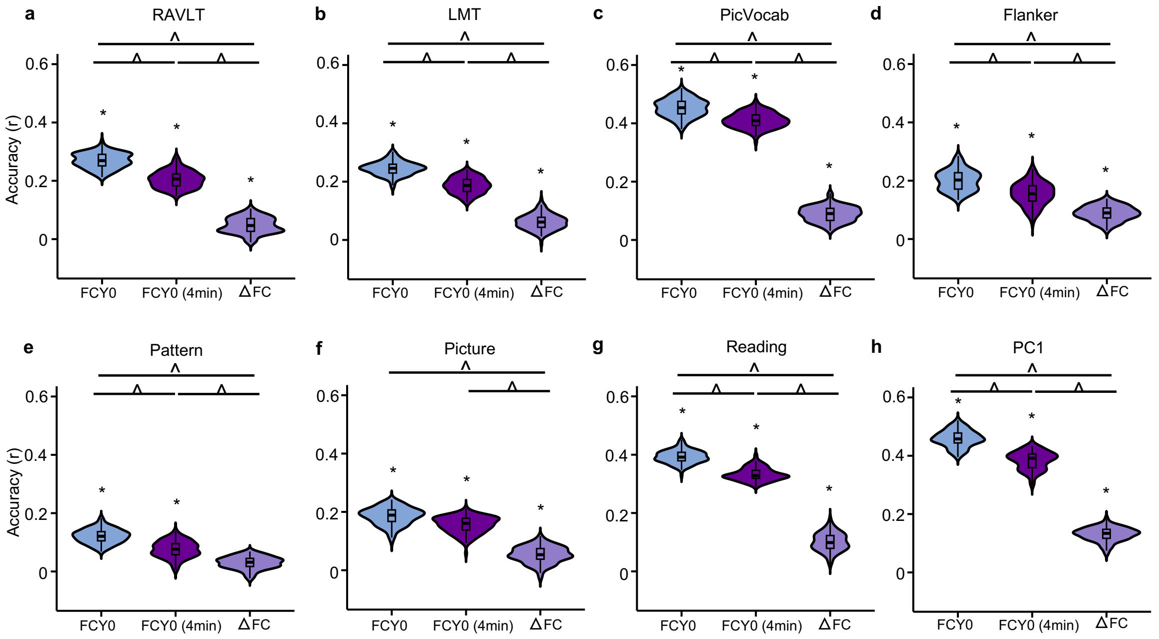

Figure S12. Baseline FC is more predictive of cognition at Year 2 than longitudinal FC change, even accounting for reliability differences. (a–h) Each panel corresponds to the prediction accuracy of a different cognitive measure at Year 2. "FCY0" are the models obtained by training baseline FC to predict Year 2 cognition (FCY0 → CogY2, blue). "FC (4min)" uses baseline FC computed from the first 4 minutes of fMRI data to predict Year 2 cognition (FCY0 (4min) → CogY2, purple). "ΔFC" are the models obtained by using FC change (between Year 2 and baseline) to predict cognition at Year 2 (ΔFC → CogY2, light purple). Each value in the violin plot represents accuracy (r) for a single cross-validation fold. Inset box plots indicate the median (center line), interquartile range (25th–75th percentile; box), and whiskers extending to 1.5 × the interquartile range, with outliers not shown. Asterisks (*) denote above chance prediction after multiple comparisons correction (FDR $q < 0.05$). Carets (^) denote statistically significant differences between models based on the corrected resampled t-test (FDR $q < 0.05$). RAVLT: Rey Auditory Verbal Learning Test (verbal memory); LMT: Little Man Task (spatial reasoning); PicVocab: Picture Vocabulary Task (vocabulary); Flanker: Flanker Task (executive function); Pattern: Pattern Comparison Processing Speed Test (processing speed); Picture: Picture Sequence Memory Test (episodic memory); Reading: Oral Reading Recognition Task (reading ability). PC1: the first principal component of the above seven cognitive measures.

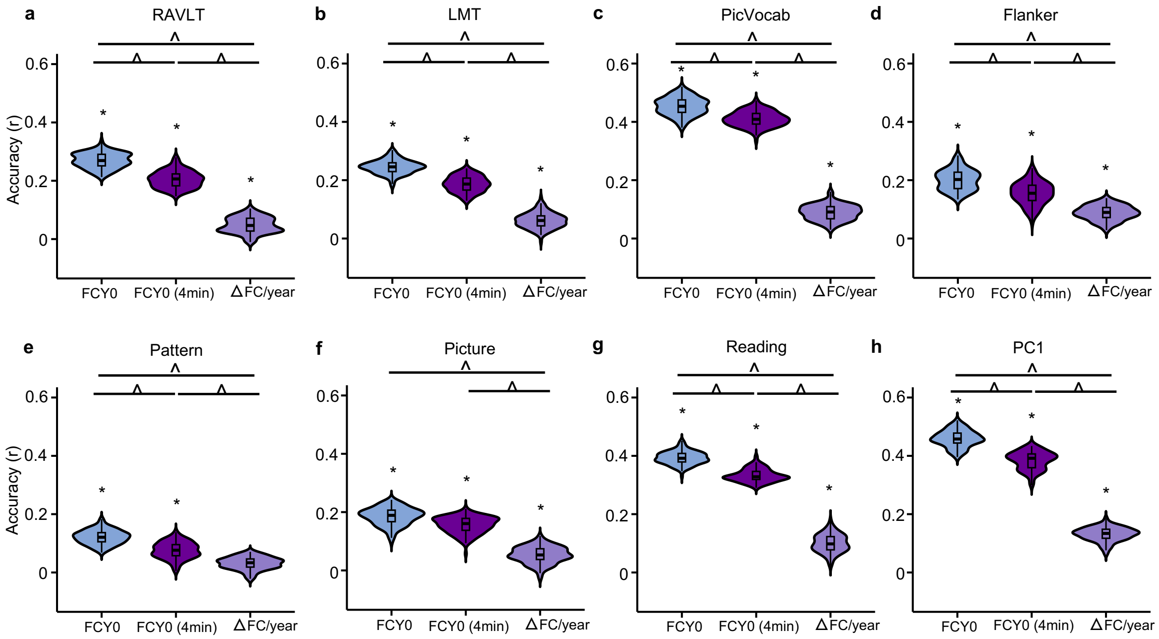

Figure S13. Baseline FC is more predictive of cognition at Year 2 than **rate of FC change**. Rate of FC change (ΔFC/year) was used instead of the FC change (delta) in this validation. (a–h) Each panel corresponds to the prediction accuracy of a different cognitive measure at Year 2. "FCY0" are the models obtained by training baseline FC to predict Year 2 cognition (FCY0 → CogY2, blue). "FC (4min)" uses baseline FC computed from the first 4 minutes of fMRI data to predict Year 2 cognition (FCY0 (4min) → CogY2, purple). "ΔFC/year" are the models obtained by using rate of FC change (between Year 2 and baseline) to predict cognition at Year 2 (ΔFC/year → CogY2, light purple). Each value in the violin plot represents accuracy (r) for a single cross-validation fold. Inset box plots indicate the median (center line), interquartile range (25th–75th percentile; box), and whiskers extending to 1.5 × the interquartile range, with outliers not shown.. Asterisks (*) denote above chance prediction after multiple comparisons correction (FDR $q < 0.05$). Carets (^) denote statistically significant differences between models based on the corrected resampled t-test (FDR $q < 0.05$). RAVLT: Rey Auditory Verbal Learning Test (verbal memory); LMT: Little Man Task (spatial reasoning); PicVocab: Picture Vocabulary Task (vocabulary); Flanker: Flanker Task (executive function); Pattern: Pattern Comparison Processing Speed Test (processing speed); Picture: Picture Sequence Memory Test (episodic memory); Reading: Oral Reading Recognition Task (reading ability). PC1: the first principal component of the above seven cognitive measures.

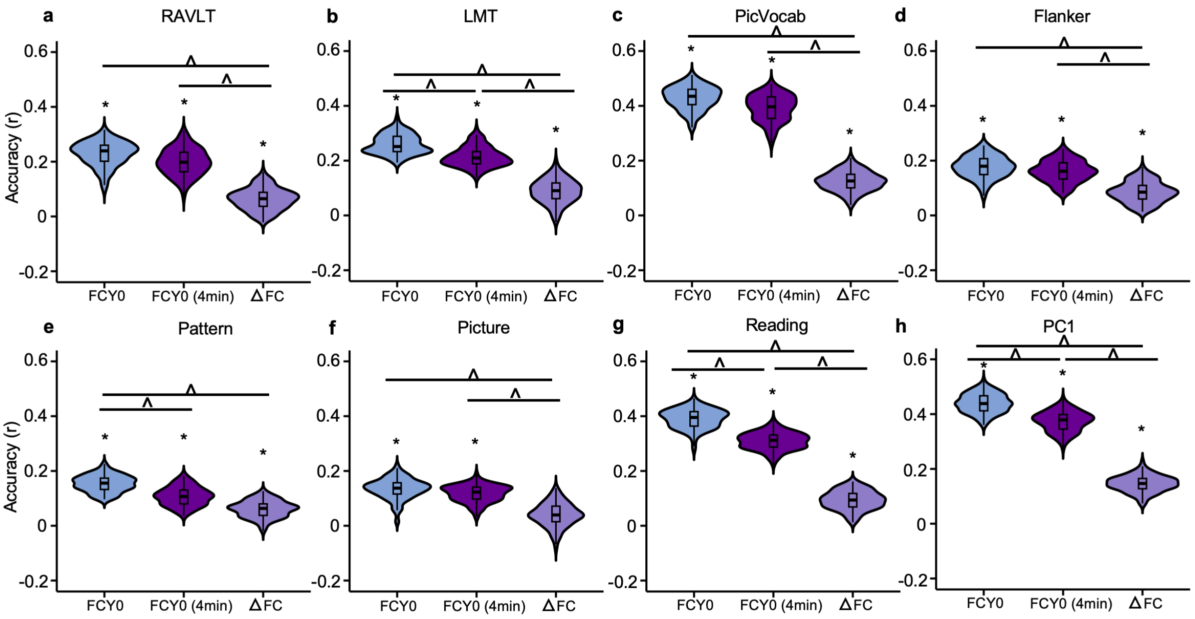

Figure S14. Baseline FC is more predictive of cognition at Year 2 than longitudinal FC change in **females**, even accounting for reliability differences. (a–h) Each panel corresponds to the prediction accuracy of a different cognitive measure at Year 2. "FCY0" are the models obtained by training baseline FC to predict Year 2 cognition (FCY0 → CogY2, blue). "FC (4min)" uses baseline FC computed from the first 4 minutes of fMRI data to predict Year 2 cognition (FCY0 (4min) → CogY2, purple). "ΔFC" are the models obtained by using FC change (between Year 2 and baseline) to predict cognition at Year 2 (ΔFC → CogY2, light purple). Each value in the violin plot represents accuracy (r) for a single cross-validation fold. Inset box plots indicate the median (center line), interquartile range (25th–75th percentile; box), and whiskers extending to 1.5 × the interquartile range, with outliers not shown. Asterisks (*) denote above chance prediction after multiple comparisons correction (FDR $q < 0.05$). Carets (^) denote statistically significant differences between models based on the corrected resampled t-test (FDR $q < 0.05$). RAVLT: Rey Auditory Verbal Learning Test (verbal memory); LMT: Little Man Task (spatial reasoning); PicVocab: Picture Vocabulary Task (vocabulary); Flanker: Flanker Task (executive function); Pattern: Pattern Comparison Processing Speed Test (processing speed); Picture: Picture Sequence Memory Test (episodic memory); Reading: Oral Reading Recognition Task (reading ability). PC1: the first principal component of the above seven cognitive measures.

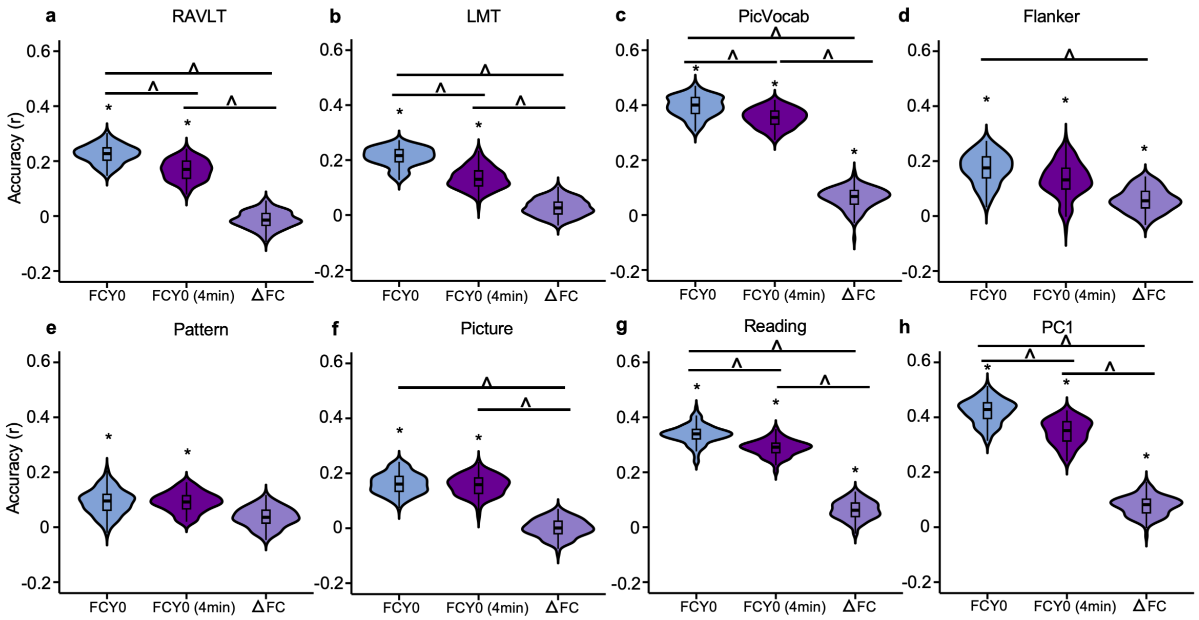

Figure S15. Baseline FC is more predictive of cognition at Year 2 than longitudinal FC change in **males**, even accounting for reliability differences. (a–h) Each panel corresponds to the prediction accuracy of a different cognitive measure at Year 2. "FCY0" are the models obtained by training baseline FC to predict Year 2 cognition (FCY0 → CogY2, blue). "FC (4min)" uses baseline FC computed from the first 4 minutes of fMRI data to predict Year 2 cognition (FCY0 (4min) → CogY2, purple). "ΔFC" are the models obtained by using FC change (between Year 2 and baseline) to predict cognition at Year 2 (ΔFC → CogY2, light purple). Each value in the violin plot represents accuracy (r) for a single cross-validation fold. Inset box plots indicate the median (center line), interquartile range (25th–75th percentile; box), and whiskers extending to 1.5 × the interquartile range, with outliers not shown. Asterisks (*) denote above chance prediction after multiple comparisons correction (FDR $q < 0.05$). Carets (^) denote statistically significant differences between models based on the corrected resampled t-test (FDR $q < 0.05$). RAVLT: Rey Auditory Verbal Learning Test (verbal memory); LMT: Little Man Task (spatial reasoning); PicVocab: Picture Vocabulary Task (vocabulary); Flanker: Flanker Task (executive function); Pattern: Pattern Comparison Processing Speed Test (processing speed); Picture: Picture Sequence Memory Test (episodic memory); Reading: Oral Reading Recognition Task (reading ability). PC1: the first principal component of the above seven cognitive measures.

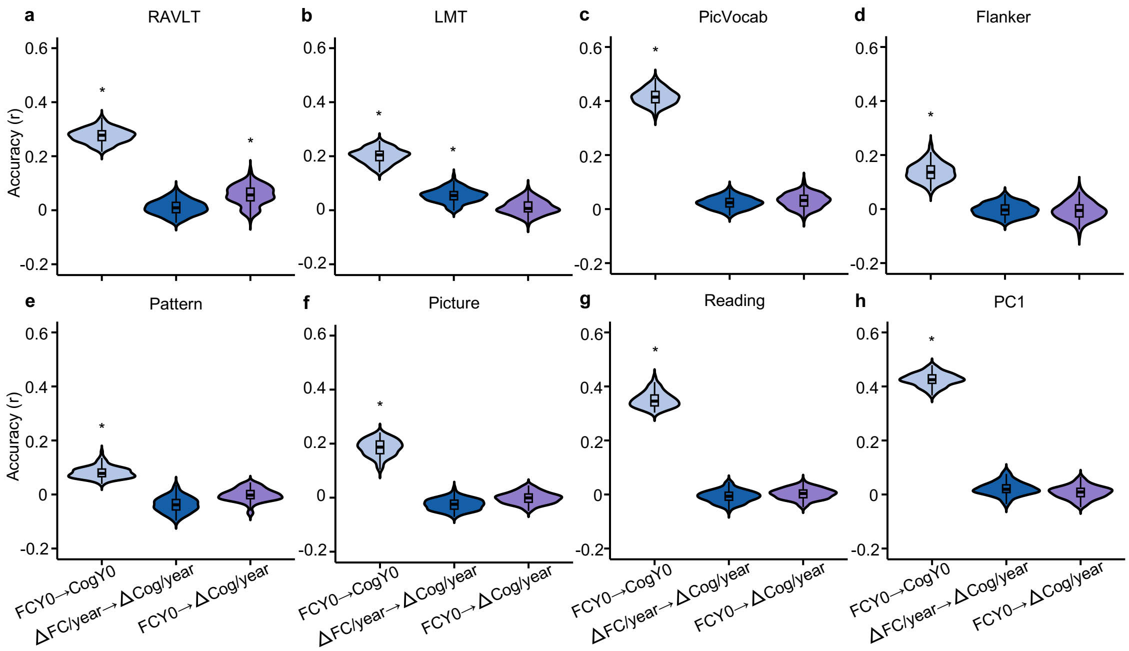

Figure S16. Limited prediction of rate of cognitive change from baseline FC and rate of FC change. (a–h) Each panel corresponds to the prediction accuracy of a different cognitive measure. Baseline FC and longitudinal rate of FC change (ΔFC/year) are used to predict rate of cognitive change (ΔCog/year). Predictions of baseline cognition from baseline FC are also shown for reference. Colours indicate different prediction settings: baseline FC predicting baseline cognition (FCY0 → CogY0, light blue), rate of FC change predicting rate of cognitive change (ΔFC/year → ΔCog/year, blue), and baseline FC predicting rate of cognitive change (FCY0 → ΔCog/year, purple). Each value in the violin plot represents prediction accuracy (r) for a single cross-validation fold. Inset box plots indicate the median (center line), interquartile range (25th–75th percentile; box), and whiskers extending to 1.5 × the interquartile range, with outliers not shown. Asterisks (*) denote above chance prediction after multiple comparisons correction (FDR $q < 0.05$). RAVLT: Rey Auditory Verbal Learning Test (verbal memory); LMT: Little Man Task (spatial reasoning); PicVocab: Picture Vocabulary Task (vocabulary); Flanker: Flanker Task (executive function); Pattern: Pattern Comparison Processing Speed Test (processing speed); Picture: Picture Sequence Memory Test (episodic memory); Reading: Oral Reading Recognition Task (reading ability). PC1: the first principal component of the above seven cognitive measures.

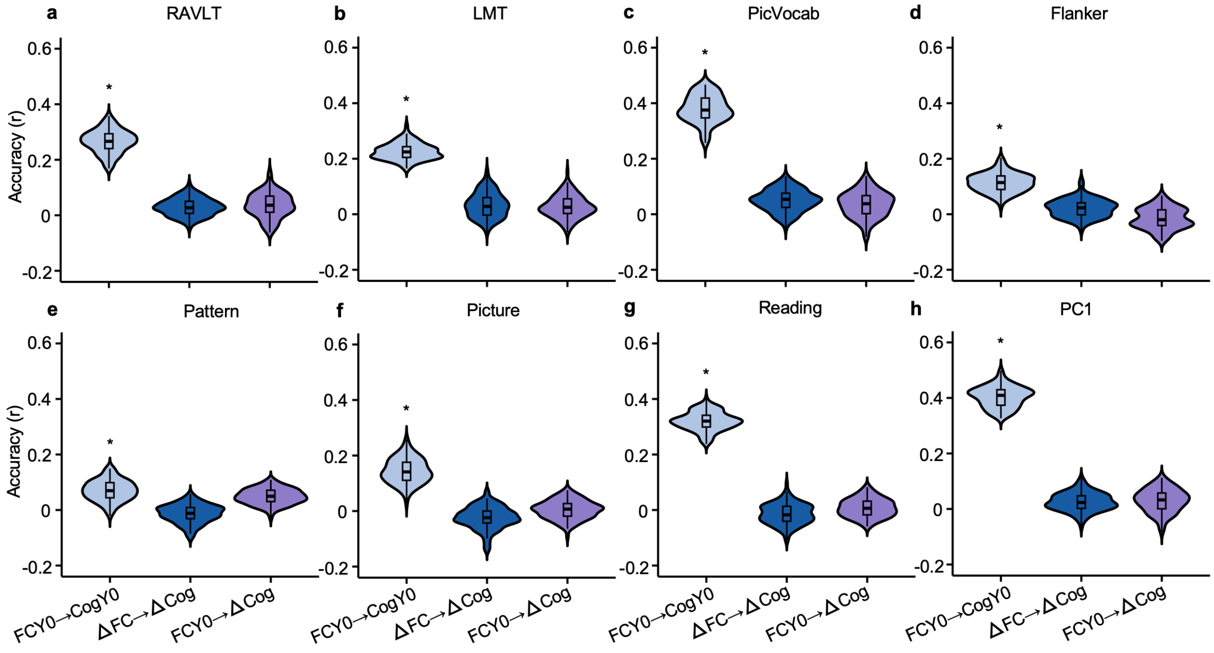

Figure S17. Limited prediction of cognitive change from baseline FC and longitudinal FC change in **females**. (a–h) Each panel corresponds to the prediction accuracy of a different cognitive measure. Baseline FC and longitudinal FC change (ΔFC) are used to predict cognitive change (ΔCog). Predictions of baseline cognition from baseline FC are also shown for reference. Colours indicate different prediction settings: baseline FC predicting baseline cognition (FCY0 → CogY0, light blue), FC change predicting cognitive change (ΔFC → ΔCog, blue), and baseline FC predicting cognitive change (FCY0 → ΔCog, purple). Each value in the violin plot represents prediction accuracy (r) for a single cross-validation fold. Inset box plots indicate the median (center line), interquartile range (25th–75th percentile; box), and whiskers extending to 1.5 × the interquartile range, with outliers not shown. Asterisks (*) denote above chance prediction after multiple comparisons correction (FDR $q < 0.05$). RAVLT: Rey Auditory Verbal Learning Test (verbal memory); LMT: Little Man Task (spatial reasoning); PicVocab: Picture Vocabulary Task (vocabulary); Flanker: Flanker Task (executive function); Pattern: Pattern Comparison Processing Speed Test (processing speed); Picture: Picture Sequence Memory Test (episodic memory); Reading: Oral Reading Recognition Task (reading ability). PC1: the first principal component of the above seven cognitive measures.

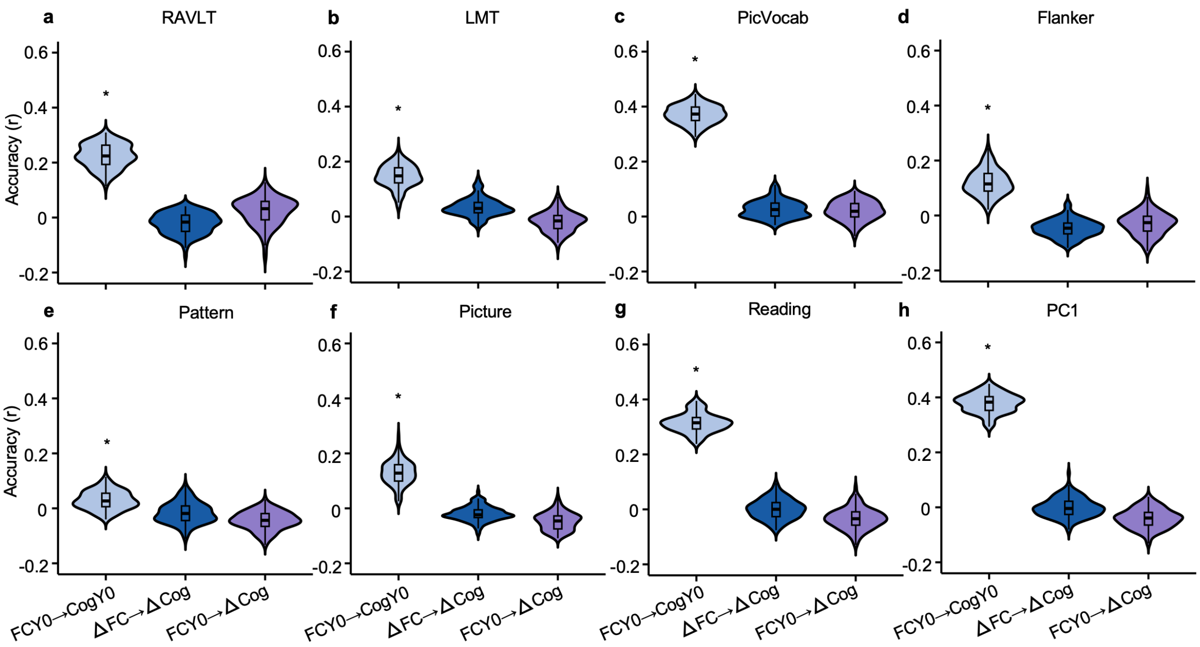

Figure S18. Limited prediction of cognitive change from baseline FC and longitudinal FC change in **males**. (a–h) Each panel corresponds to the prediction accuracy of a different cognitive measure. Baseline FC and longitudinal FC change (ΔFC) are used to predict cognitive change (ΔCog). Predictions of baseline cognition from baseline FC are also shown for reference. Colours indicate different prediction settings: baseline FC predicting baseline cognition (FCY0 → CogY0, light blue), FC change predicting cognitive change (ΔFC → ΔCog, blue), and baseline FC predicting cognitive change (FCY0 → ΔCog, purple). Each value in the violin plot represents prediction accuracy (r) for a single cross-validation fold. Inset box plots indicate the median (center line), interquartile range (25th–75th percentile; box), and whiskers extending to 1.5 × the interquartile range, with outliers not shown. Asterisks (*) denote above chance prediction after multiple comparisons correction (FDR $q < 0.05$). RAVLT: Rey Auditory Verbal Learning Test (verbal memory); LMT: Little Man Task (spatial reasoning); PicVocab: Picture Vocabulary Task (vocabulary); Flanker: Flanker Task (executive function); Pattern: Pattern Comparison Processing Speed Test (processing speed); Picture: Picture Sequence Memory Test (episodic memory); Reading: Oral Reading Recognition Task (reading ability). PC1: the first principal component of the above seven cognitive measures.

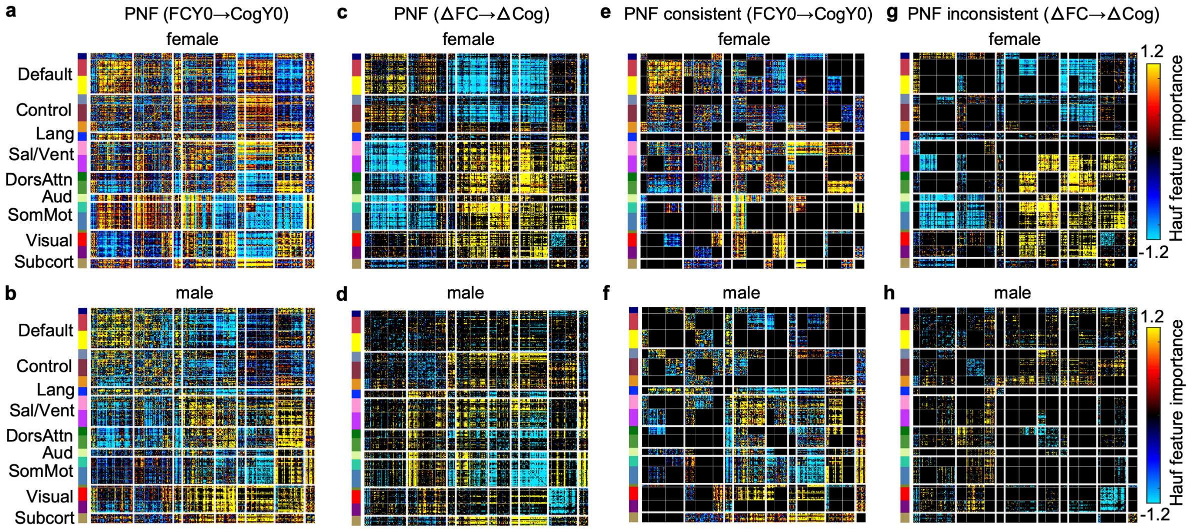

Figure S19. Convergent and divergent predictive network features (PNFs) between cross-sectional and longitudinal estimates of FC–cognition relationship within each sex. This figure is the same as Figure 8, except that only PNF values that survived FDR $q < 0.05$ are shown. (a-b) PNFs from cross-sectional models using baseline FC (FCY0) to predict baseline Little Man Task (LMT) score (CogY0) in (a) females and (b) males separately. Values were thresholded with FDR $q < 0.05$. (c-d) PNFs from longitudinal models using changes in FC (ΔFC) to predict changes in LMT performance (ΔCog) across the two timepoints in (c) females and (d) males separately. Values were thresholded with FDR $q < 0.05$. (e-f) Network blocks with consistent PNFs between cross-sectional and longitudinal models in (e) females and (f) males. (g-h) Network blocks with inconsistent PNFs between cross-sectional and longitudinal models in (g) females and (h) males. PNFs were considered consistent if the average feature value for a network block had the same sign in both cross-sectional and longitudinal models. For visualization purposes, each predictive network feature matrix was normalized by dividing all values by the standard deviation of the entire matrix.

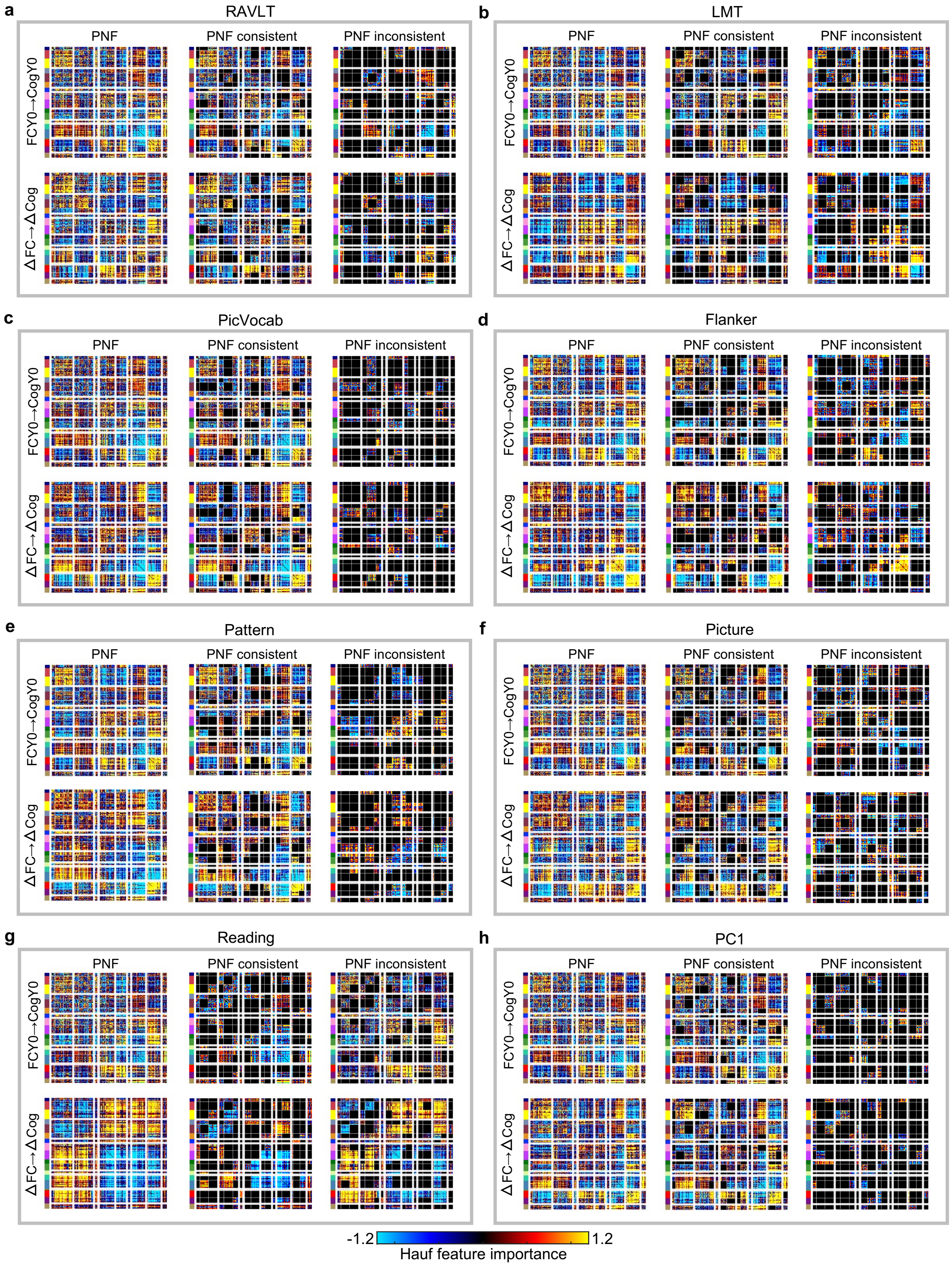

Figure S20. Convergent and divergent predictive network features (PNFs) between cross-sectional and longitudinal estimates of FC-cognition relationships for the eight cognitive measures. (a-h) Each grey box represents one cognitive measure. Within each box, the first row corresponds to the

cross-sectional model (FCY0 → CogY0), and the second row corresponds to the longitudinal model (ΔFC → ΔCog). The PNF column displays the predictive network features for each model. The PNF consistent column highlights network blocks where the average PNFs have the same sign in both models, indicating convergence. The PNF inconsistent column highlights blocks where the average PNFs have opposite signs, indicating divergence. RAVLT: Rey Auditory Verbal Learning Test (verbal memory); LMT: Little Man Task (spatial reasoning); PicVocab: Picture Vocabulary Task (vocabulary); Flanker: Flanker Task (executive function); Pattern: Pattern Comparison Processing Speed Test (processing speed); Picture: Picture Sequence Memory Test (episodic memory); Reading: Oral Reading Recognition Task (reading ability). PC1: the first principal component of the above seven cognitive measures. For visualization purposes, each predictive network feature matrix was normalized by dividing all values by the standard deviation of the entire matrix.

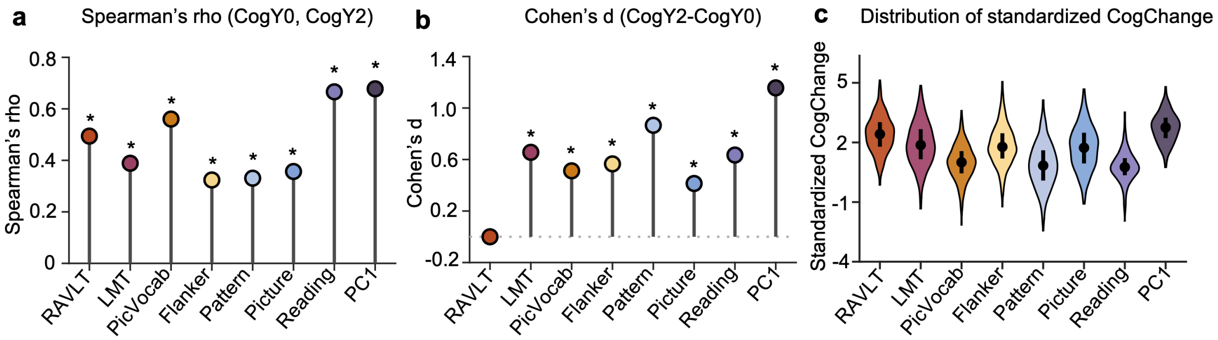

Figure S21. Individual differences in longitudinal cognitive change during the transition from childhood to adolescence in a representative subsample (N = 2,020). (a) Spearman's correlation between baseline (CogY0) and Year 2 (CogY2) cognitive measures. Each marker represents a cognitive measure. Positive correlations indicate that children with higher baseline cognition generally maintained their cognitive advantage over their peers at Year 2. (b) Longitudinal cognitive change at the group level estimated from a linear mixed effects model. Values represent Cohen's d. (c) Individual variability in standardized longitudinal cognitive change. For each individual and each cognitive measure, cognition change was defined as the difference in scores between the two timepoints. Sex, baseline age and age interval (between baseline and Year 2) were regressed out. The residualized change scores were then standardized by the baseline standard deviation of each measure. Violin plots show the distribution of standardized cognitive change, with dots indicating the median and bars indicating the interquartile range (25th–75th percentile). Colours distinguish different cognitive measures. Distributions are shown for descriptive purposes only.

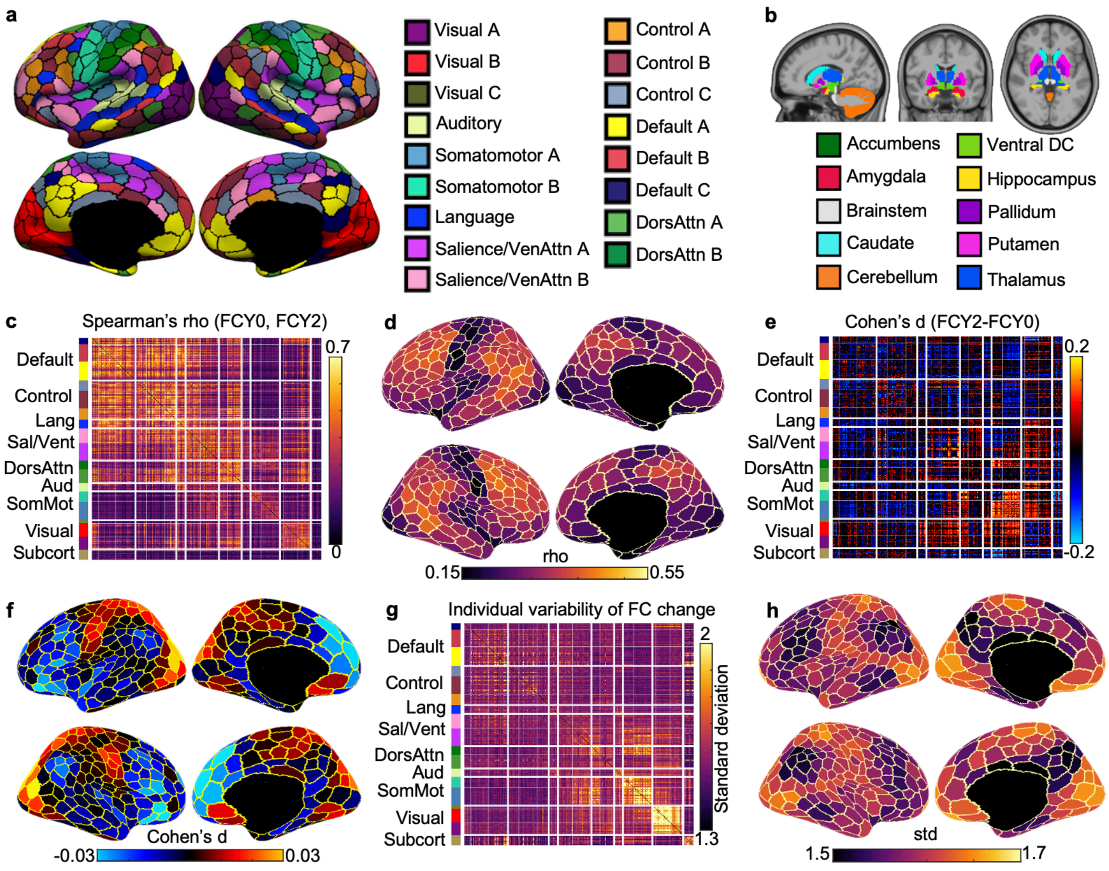

Figure S22. Individual differences in longitudinal functional connectivity (FC) change in a representative subsample (N = 2,020). (a) Cortical parcellation of 400 regions[4], which is the homotopic variant of the Schaefer parcellation[5]. Parcel colours are assigned corresponding to 17 large-scale networks[6]. (b) 19 subcortical regions[7]. Colours denote different subcortical structures. 419 × 419 FC matrices were computed based on the 419 cortical and subcortical regions. (c) Spearman's correlation (stability) between baseline (FCY0) and Year 2 FC (FCY2) for each FC edge. Positive correlations (rho; warmer colours) indicate that children exhibiting stronger brain connectivity at baseline continued to exhibit stronger brain connectivity than their peers at Year 2. 99.995% of entries were significant after FDR correction with $q < 0.05$. (d) Visualization of FC stability at the regional level, by averaging the rows of panel (c). (e) Longitudinal FC change at the group level based on a linear mixed effects model. 54.96% of entries were significant after FDR correction with $q < 0.05$. Values are shown as Cohen's d. (f) Visualization of longitudinal FC change at the regional level, by averaging the rows of panel (e). (g) Individual variability in longitudinal FC change. FC change (z value) was computed for each FC edge[2]. Sex, baseline age, age interval (between baseline and Year 2), and head motion at two timepoints were regressed out. Standard deviation was then computed across individuals. (h) Visualization of individual variability in longitudinal FC change at the regional level, by averaging the rows of panel (g).

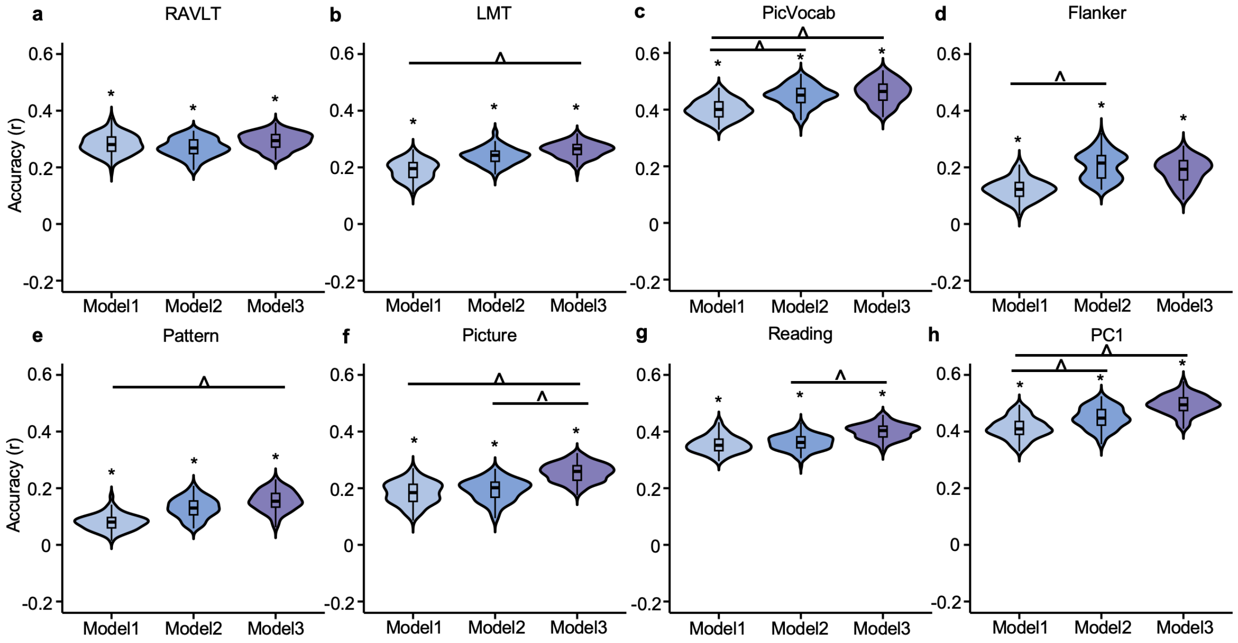

Figure S23. Enhanced FC–cognition relationships during development in a representative subsample (N = 2,020). (a-h) Comparison of prediction accuracies across the three models for the eight cognitive measures. Model 1 predicts baseline cognition using baseline FC (FCY0 → CogY0, light blue). Model 2 predicts Year 2 cognition using baseline FC (FCY0 → CogY2, blue). Model 3 predicts Year 2 cognition using Year 2 FC (FCY2 → CogY2, purple). Each value in the violin plot represents accuracy (r) for a single cross-validation fold. Inset box plots indicate the median (center line), interquartile range (25th–75th percentile; box), and whiskers extending to 1.5 × the interquartile range, with outliers not shown. Asterisks (*) denote above chance prediction after multiple comparisons correction (FDR $q < 0.05$). Carets (^) denote statistically significant differences between models based on the corrected resampled t-test (FDR $q < 0.05$). RAVLT: Rey Auditory Verbal Learning Test (verbal memory); LMT: Little Man Task (spatial reasoning); PicVocab: Picture Vocabulary Task (vocabulary); Flanker: Flanker Task (executive function); Pattern: Pattern Comparison Processing Speed Test (processing speed); Picture: Picture Sequence Memory Test (episodic memory); Reading: Oral Reading Recognition Task (reading ability). PC1: the first principal component of the above seven cognitive measures.

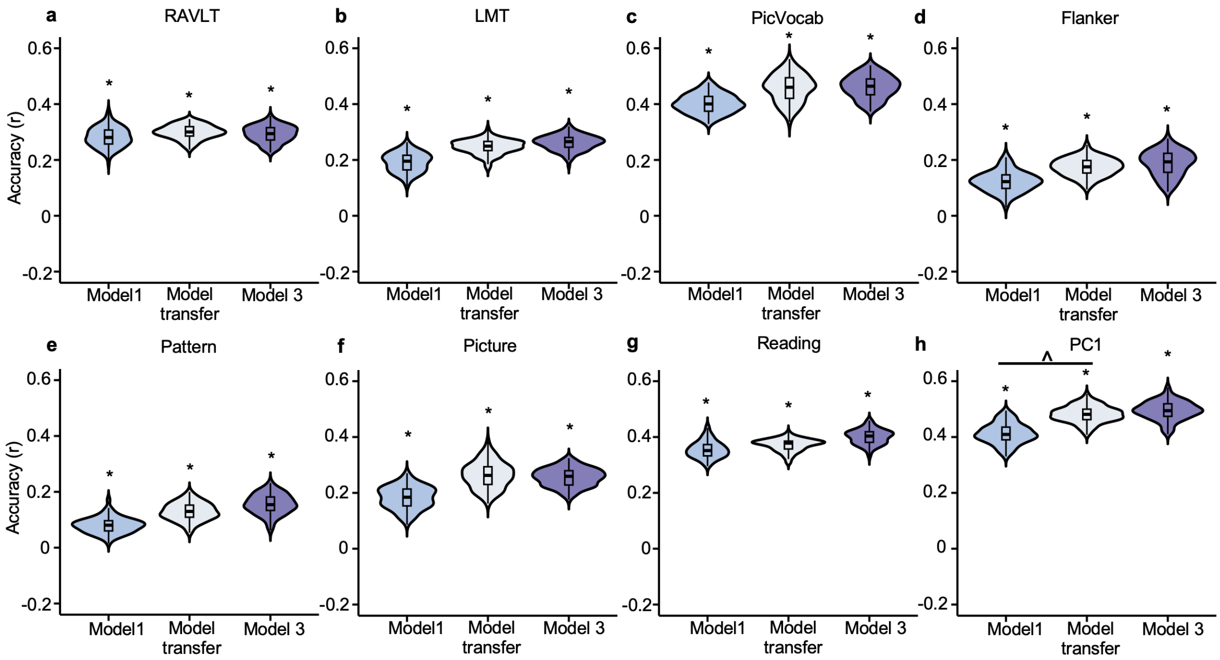

Figure S24. Models trained on baseline FC to predict baseline cognition improve in accuracy when applied to Year 2 FC and Year 2 cognition in a representative subsample (N = 2,020). (a–h) Each plot corresponds to a different cognitive measure. "Model 1" are the models obtained by training baseline FC to predict baseline cognition (FCY0 → CogY0, light blue). These models are then used to predict Y2 cognition from Year 2 FC, which we refer to as "Model transfer" (grey violin). Finally, for reference, "Model 3" are the models obtained by training Year 2 FC to predict Year 2 cognition (FCY2 → CogY2, purple). Each value in the violin plot represents the accuracy (r) for a single cross-validation fold. Inset box plots indicate the median (center line), interquartile range (25th–75th percentile; box), and whiskers extending to 1.5 × the interquartile range, with outliers not shown. Asterisks (*) denote above chance prediction after multiple comparisons correction (FDR $q < 0.05$). Carets (^) denote statistically significant differences between models based on the corrected resampled t-test (FDR $q < 0.05$). RAVLT: Rey Auditory Verbal Learning Test (verbal memory); LMT: Little Man Task (spatial reasoning); PicVocab: Picture Vocabulary Task (vocabulary); Flanker: Flanker Task (executive function); Pattern: Pattern Comparison Processing Speed Test (processing speed); Picture: Picture Sequence Memory Test (episodic memory); Reading: Oral Reading Recognition Task (reading ability). PC1: the first principal component of the above seven cognitive measures.

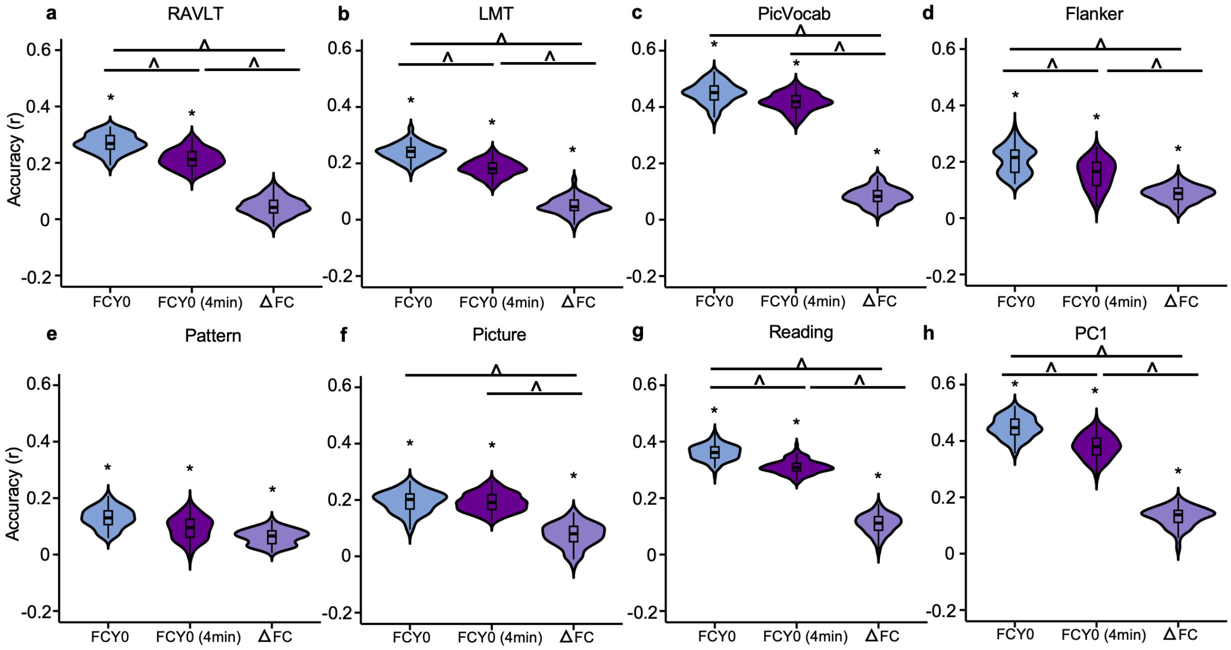

Figure S25. Baseline FC is more predictive of cognition at Year 2 than longitudinal FC change, even accounting for reliability differences in a representative subsample (N = 2,020). (a–h) Each panel corresponds to the prediction accuracy of a different cognitive measure at Year 2. "FCY0" are the models obtained by training baseline FC to predict Year 2 cognition (FCY0 → CogY2, blue). "FC (4min)" uses baseline FC computed from the first 4 minutes of fMRI data to predict Year 2 cognition (FCY0 (4min) → CogY2, purple). "ΔFC" are the models obtained by using FC change (between Year 2 and baseline) to predict cognition at Year 2 (ΔFC → CogY2, light purple). Each value in the violin plot represents accuracy (r) for a single cross-validation fold. Inset box plots indicate the median (center line), interquartile range (25th–75th percentile; box), and whiskers extending to 1.5 × the interquartile range, with outliers not shown. Asterisks (*) denote above chance prediction after multiple comparisons correction (FDR q < 0.05). Carets (^) denote statistically significant differences between models based on the corrected resampled t-test (FDR q < 0.05). RAVLT: Rey Auditory Verbal Learning Test (verbal memory); LMT: Little Man Task (spatial reasoning); PicVocab: Picture Vocabulary Task (vocabulary); Flanker: Flanker Task (executive function); Pattern: Pattern Comparison Processing Speed Test (processing speed); Picture: Picture Sequence Memory Test (episodic memory); Reading: Oral Reading Recognition Task (reading ability). PC1: the first principal component of the above seven cognitive measures.

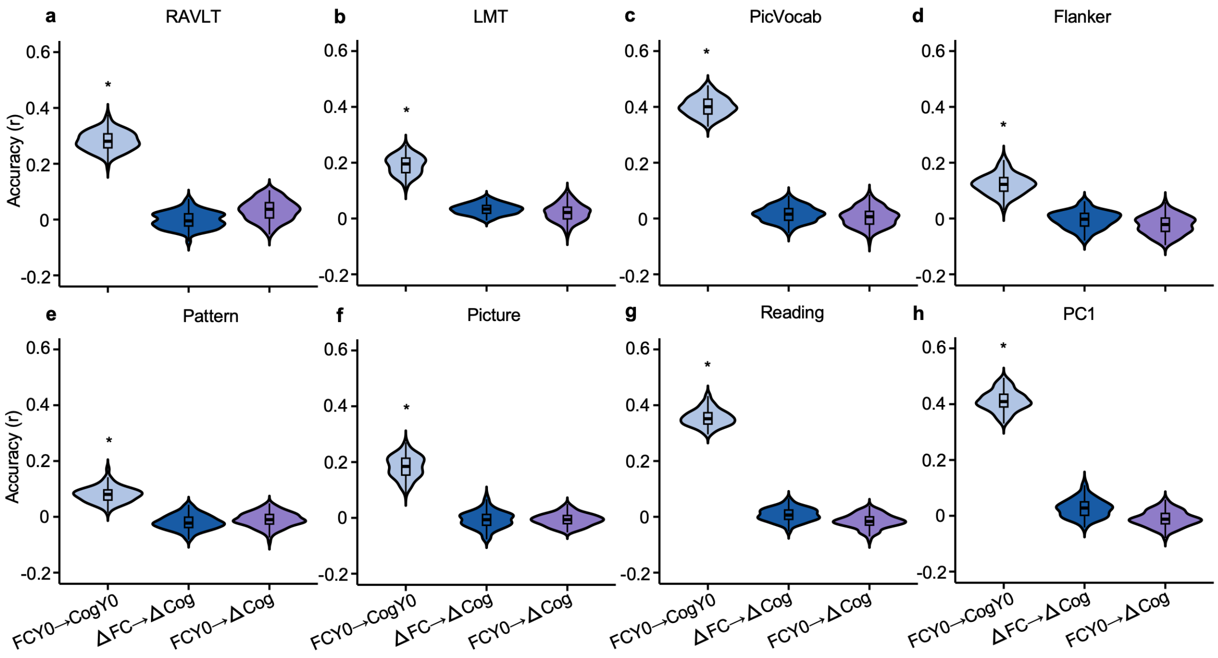

Figure S26. Limited prediction of cognitive change from baseline FC and longitudinal FC change in a representative subsample (N = 2,020). (a–h) Each panel corresponds to the prediction accuracy of a different cognitive measure. Baseline FC and longitudinal FC change (ΔFC) are used to predict cognitive change (ΔCog). Predictions of baseline cognition from baseline FC are also shown for reference. Colours indicate different prediction settings: baseline FC predicting baseline cognition (FCY0 → CogY0, light blue), FC change predicting cognitive change (ΔFC → ΔCog, blue), and baseline FC predicting cognitive change (FCY0 → ΔCog, purple). Each value in the violin plot represents prediction accuracy (r) for a single cross-validation fold. Inset box plots indicate the median (center line), interquartile range (25th–75th percentile; box), and whiskers extending to 1.5 × the interquartile range, with outliers not shown. Asterisks (*) denote above chance prediction after multiple comparisons correction (FDR $q < 0.05$). RAVLT: Rey Auditory Verbal Learning Test (verbal memory); LMT: Little Man Task (spatial reasoning); PicVocab: Picture Vocabulary Task (vocabulary); Flanker: Flanker Task (executive function); Pattern: Pattern Comparison Processing Speed Test (processing speed); Picture: Picture Sequence Memory Test (episodic memory); Reading: Oral Reading Recognition Task (reading ability). PC1: the first principal component of the above seven cognitive measures.

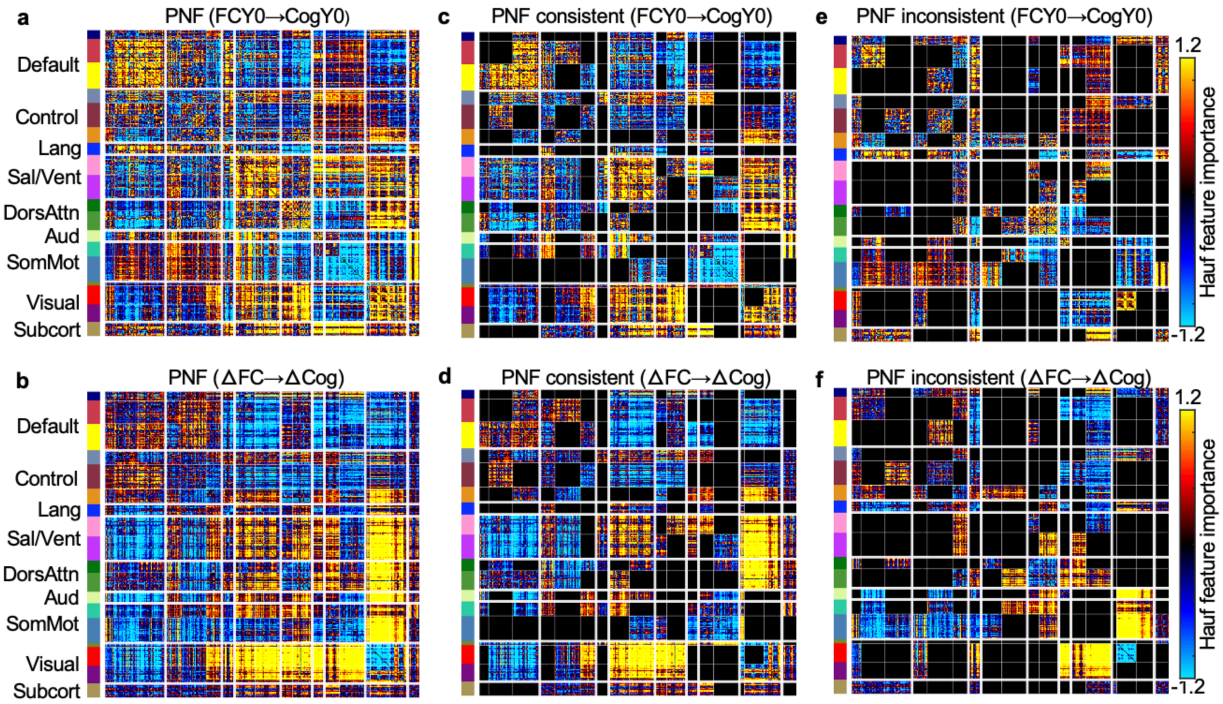

Figure S27. Convergent and divergent predictive network features (PNFs) between cross-sectional and longitudinal estimates of FC–cognition relationship in a representative subsample (N = 2,020). (a) PNFs from cross-sectional model using baseline FC (FCY0) to predict baseline Little Man Task (LMT) score (CogY0). (b) PNFs from longitudinal model using changes in FC (ΔFC) to predict changes in LMT performance (ΔCog) across the two timepoints. (c) Network blocks with consistent PNFs in the cross-sectional model. (d) Network blocks with consistent PNFs in the longitudinal model. PNFs were considered consistent if the average feature value for a network block had the same sign in both cross-sectional and longitudinal models. (e) Network blocks with inconsistent PNFs in the cross-sectional model. (f) Network blocks with inconsistent PNFs in the longitudinal model. PNFs were considered inconsistent if the average feature value had opposite signs across models.

Supplementary Tables

Table S1. Linear mixed-effects model results for females, males and sex differences in cognitive change.

	Female				Male				Sex differences			
	β	SE	t	p	β	SE	t	p	β	SE	t	p
RAVLT	-0.07119	0.01766	-4.03	< **0.001**	0.03784	0.01734	2.18	**0.03**	-0.10903	0.02475	-4.41	< **0.001**
LMT	0.00580	0.00023	24.96	< **0.001**	0.00587	0.00023	25.72	< **0.001**	-0.00007	0.00033	-0.22	0.82
PicVocab	0.16511	0.00854	19.34	< **0.001**	0.18394	0.00839	21.92	< **0.001**	-0.01882	0.01197	-1.57	0.12
Flanker	0.23165	0.01093	21.20	< **0.001**	0.22971	0.01073	21.41	< **0.001**	0.00194	0.01531	0.13	0.90
Pattern	0.63692	0.01927	33.04	< **0.001**	0.61494	0.01894	32.47	< **0.001**	0.02198	0.02702	0.81	0.42
Picture	0.23247	0.01661	14.00	< **0.001**	0.26548	0.01631	16.28	< **0.001**	-0.03301	0.02328	-1.42	0.16
Reading	0.15380	0.00663	23.19	< **0.001**	0.15538	0.00652	23.85	< **0.001**	-0.00158	0.00930	-0.17	0.86
PC1	0.05677	0.00135	41.99	< **0.001**	0.06132	0.00133	46.18	< **0.001**	-0.00455	0.00189	-2.40	**0.02**

Note that bold p-values denote effects that remained significant (two-sided tests) after false discovery rate (FDR) correction at $q < 0.05$. LMT: Little Man Task (spatial reasoning); PicVocab: Picture Vocabulary Task (vocabulary); Flanker: Flanker Task (executive function); Pattern: Pattern Comparison Processing Speed Test (processing speed); Picture: Picture Sequence Memory Test (episodic memory); Reading: Oral Reading Recognition Task (reading ability). PC1: the first principal component of the above seven cognitive measures.

Table S2. Linear mixed-effects model results for relationship between motion and cognition

	mean FD				std FD			
	β	SE	t	p	β	SE	t	p
RAVLT	-0.00008	0.00003	-2.48	**0.01306**	-0.00019	0.00008	-2.34	**0.01931**
LMT	-0.00450	0.00275	-1.64	0.10130	-0.01614	0.00659	-2.45	**0.01436**
PicVocab	-0.00012	0.00006	-1.97	0.04917	-0.00028	0.00015	-1.88	0.05994
Flanker	-0.00013	0.00006	-2.07	**0.03848**	-0.00033	0.00015	-2.29	**0.02209**
Pattern	-0.00010	0.00003	-3.11	**0.00187**	-0.00031	0.00008	-3.80	**< 0.001**
Picture	-0.00011	0.00004	-2.89	**0.00384**	-0.00028	0.00009	-2.95	**0.00322**
Reading	-0.00017	0.00008	-2.28	**0.02282**	-0.00037	0.00018	-2.05	**0.04047**
PC1	-0.00141	0.00034	-4.11	**< 0.001**	-0.00354	0.00081	-4.36	**< 0.001**

Note that bold p-values denote effects that remained significant (two-sided tests) after false discovery rate (FDR) correction at $q < 0.05$. LMT: Little Man Task (spatial reasoning); PicVocab: Picture Vocabulary Task (vocabulary); Flanker: Flanker Task (executive function); Pattern: Pattern Comparison Processing Speed Test (processing speed); Picture: Picture Sequence Memory Test (episodic memory); Reading: Oral Reading Recognition Task (reading ability). PC1: the first principal component of the above seven cognitive measures.

Table S3. Prediction accuracy for each cognitive measure and each cross-sectional model

	FCY0-CogY0	FCY0-CogY2	FCY2-CogY2	ModelTransfer	FCY2-CogY0
RAVLT	0.28	0.27	0.30	0.31	0.28
LMT	0.20	0.25	0.26	0.25	0.22
PicVocab	0.42	0.45	0.47	0.46	0.42
Flanker	0.14	0.20	0.19	0.17	0.12
Pattern	0.08	0.12	0.15	0.12	0.10
Picture	0.19	0.19	0.24	0.25	0.18
Reading	0.35	0.39	0.41	0.39	0.36
PC1	0.43	0.46	0.49	0.48	0.44

Values in each cell indicate the prediction accuracy under each condition. FCY0-CogY0: predicting baseline cognition from baseline FC; FCY0-CogY2: predicting Year 2 cognition from baseline FC; FCY2-CogY2: predicting Year 2 cognition from Year 2 FC; ModelTransfer: Model 1 applied to Year 2 FC to predict Year 2 cognition; FCY2-CogY0: predicting baseline cognition from Year 2 FC, included as a control to test whether higher-quality fMRI data at Year 2 compared with baseline improve prediction accuracy for baseline cognition. LMT: Little Man Task (spatial reasoning); PicVocab: Picture Vocabulary Task (vocabulary); Flanker: Flanker Task (executive function); Pattern: Pattern Comparison Processing Speed Test (processing speed); Picture: Picture Sequence Memory Test (episodic memory); Reading: Oral Reading Recognition Task (reading ability). PC1: the first principal component of the above seven cognitive measures.

Table S4. Compare distribution of included and excluded participants after matching

Variable	Included (Matched)	Excluded	p
Sample size (N)	2020	9848	N.A.
Age (month)	118.89 (7.40)	119 (7.52)	0.53
Female (%)	48.37	47.74	0.64
race (%)	A/B/H/W/O	A/B/H/W/O	0.03
Asian	54.21	51.57	—
Black	13.07	15.44	—
Hispanic	20.74	20.22	—
White	1.68	2.21	—
Other	10.30	10.55	—
Income (%)	L1/L2/L3/U	L1/L2/L3/U	0.51
L1: < 50K	27.13	27.15	—
L2: >= 50K & < 100K	26.14	25.79	—
L3: >=100K	39.01	38.31	—
Unknown	7.72	8.74	—
RAVLT	68.66 (15.36)	67.71(15.53)	0.03
LMT	0.60 (0.17)	0.59 (0.17)	0.01
PicVocab	84.70 (7.97)	84.40 (8.09)	0.20
Flanker	94.40 (8.62)	93.92 (9.18)	0.08
Pattern	88.13 (13.91)	88.04 (14.60)	0.73
Picture	103.20 (12.06)	102.73 (11.98)	0.17
Reading	90.98 (6.86)	90.83 (6.87)	0.26
PC1	0.09 (1.61)	-0.02 (1.66)	0.04

Values are means ± SD for continuous variables and percentages for categorical variables. For categorical variables with multiple levels (sex, race and household income), p-values reflect χ^2 tests comparing overall distributions between included and excluded participants, and sub-category rows are descriptive only. For continuous variables, p-values were obtained using two-sided Wilcoxon rank-sum tests. Baseline cognitive matching was performed using PC1; individual cognitive measures are shown for descriptive assessment of balance. There is no significant difference after multiple comparisons correction with FDR q <0.05. LMT: Little Man Task (spatial reasoning); PicVocab: Picture Vocabulary Task (vocabulary); Flanker: Flanker Task (executive function); Pattern: Pattern Comparison Processing Speed Test (processing speed); Picture: Picture Sequence Memory Test (episodic memory); Reading: Oral Reading Recognition Task (reading ability). PC1: the first principal component of the above seven cognitive measures.

Supplementary References

1. Benjamini Y, Hochberg Y. Controlling the False Discovery Rate: A Practical and Powerful Approach to Multiple Testing. *J R Stat Soc Series B Stat Methodol* **57**, 289-300 (1995).

2. Afyouni S, Smith SM, Nichols TE. Effective degrees of freedom of the Pearson's correlation coefficient under autocorrelation. *Neuroimage* **199**, 609-625 (2019).

3. Rogosa DR, Willett JB. Demonstrating the Reliability of the Difference Score in the Measurement of Change. *Journal of Educational Measurement* **20**, 335-343 (1983).

4. Yan X*, et al.* Homotopic local-global parcellation of the human cerebral cortex from resting-state functional connectivity. *NeuroImage* **273**, 120010 (2023).

5. Schaefer A*, et al.* Local-Global Parcellation of the Human Cerebral Cortex from Intrinsic Functional Connectivity MRI. *Cereb Cortex* **28**, 3095-3114 (2018).

6. Kong R*, et al.* Individual-Specific Areal-Level Parcellations Improve Functional Connectivity Prediction of Behavior. *Cereb Cortex* **31**, 4477-4500 (2021).

7. Fischl B*, et al.* Whole brain segmentation: automated labeling of neuroanatomical structures in the human brain. *Neuron* **33**, 341-355 (2002).
