# Supplementary material for: Convergent and divergent brain–cognition development in early adolescence
Source: Nat Commun. 2026 May 26;17:6868. doi: 10.1038/s41467-026-73668-y (PMC13388698; doi:10.1038/s41467-026-73668-y)
Supplement: Supplementary file 2 — Reporting Summary [file 41467_2026_73668_MOESM2_ESM.pdf]

nature portfolio

Corresponding author(s): B.T. Thomas Yeo

Last updated by author(s): Apr 24, 2026

Reporting Summary

Nature Portfolio wishes to improve the reproducibility of the work that we publish. This form provides structure for consistency and transparency in reporting. For further information on Nature Portfolio policies, see our Editorial Policies and the Editorial Policy Checklist.

Statistics

For all statistical analyses, confirm that the following items are present in the figure legend, table legend, main text, or Methods section.

n/a	Confirmed	
☐	☒	The exact sample size (*n*) for each experimental group/condition, given as a discrete number and unit of measurement
☐	☒	A statement on whether measurements were taken from distinct samples or whether the same sample was measured repeatedly
☐	☒	The statistical test(s) used AND whether they are one- or two-sided<br>*Only common tests should be described solely by name; describe more complex techniques in the Methods section.*
☐	☒	A description of all covariates tested
☐	☒	A description of any assumptions or corrections, such as tests of normality and adjustment for multiple comparisons
☐	☒	A full description of the statistical parameters including central tendency (e.g. means) or other basic estimates (e.g. regression coefficient) AND variation (e.g. standard deviation) or associated estimates of uncertainty (e.g. confidence intervals)
☐	☒	For null hypothesis testing, the test statistic (e.g. *F*, *t*, *r*) with confidence intervals, effect sizes, degrees of freedom and *P* value noted<br>*Give P values as exact values whenever suitable.*
☒	☐	For Bayesian analysis, information on the choice of priors and Markov chain Monte Carlo settings
☒	☐	For hierarchical and complex designs, identification of the appropriate level for tests and full reporting of outcomes
☐	☒	Estimates of effect sizes (e.g. Cohen's *d*, Pearson's *r*), indicating how they were calculated

Our web collection on statistics for biologists contains articles on many of the points above.

Software and code

Policy information about availability of computer code

Data collection	Data were obtained from the publicly available ABCD Study; no software was used by the authors for data collection.
Data analysis	Data analysis was performed using MATLAB (2018b), FreeSurfer (7.4.1), FSL (5.0.10), and RStudio (2022.12.0).Image preprocessing was conducted using previously published pipelines available at https://github.com/ThomasYeoLab/Standalone_CBIG_fMRI_Preproc2016, archived at https://doi.org/10.5281/zenodo.19723245. Study-specific preprocessing scripts are available at https://github.com/ThomasYeoLab/ABCD_scripts, archived at https://doi.org/10.5281/zenodo.19723255. Analysis code is publicly available at https://github.com/ThomasYeoLab/Standalone_Xie2025_LBC and archived at https://doi.org/10.5281/zenodo.19219794.

For manuscripts utilizing custom algorithms or software that are central to the research but not yet described in published literature, software must be made available to editors and reviewers. We strongly encourage code deposition in a community repository (e.g. GitHub). See the Nature Portfolio guidelines for submitting code & software for further information.

Data

Policy information about availability of data

All manuscripts must include a data availability statement. This statement should provide the following information, where applicable:

- Accession codes, unique identifiers, or web links for publicly available datasets
- A description of any restrictions on data availability
- For clinical datasets or third party data, please ensure that the statement adheres to our policy

The ABCD data are publicly available through NIH Brain Development Cohorts (NBDC) Data Hub. Researchers with access to the ABCD data will be able to download the data from https://nbdc-datashare.lassoinformatics.com. Source data supporting the findings of this study are provided with this paper.

Research involving human participants, their data, or biological material

Policy information about studies with human participants or human data. See also policy information about sex, gender (identity/presentation), and sexual orientation and race, ethnicity and racism.

Reporting on sex and gender	The ABCD Study includes both biological sex (sex assigned at birth) and self-reported gender identity variables, though the latter were not analyzed in the present work. In this study, biological sex was operationalized using the "sex at birth" variable as reported by caregivers during baseline assessments. Analyses were conducted on the combined sample with sex treated as a covariate in all primary statistical models, and additional sex-stratified analyses were performed for all main outcome to examine sex-specific effects and sex differences.
Reporting on race, ethnicity, or other socially relevant groupings	Race and ethnicity data were available in the ABCD Study and collected via caregiver report using standard ABCD coding for race/ethnicity categories. In the present analyses, these variables were not included as covariates in the main models in order to limit model complexity and avoid potential over-control of socially constructed categories. No primary analyses were stratified by race, ethnicity, or other socially constructed groupings. Secondary subgroup analyses were conducted to demonstrate the generalizability of the main results, with subgroup samples matched on key demographic variables, including race and household income.
Population characteristics	The ABCD Study is a longitudinal, multi-site, population-based cohort of U.S. children recruited at ages 9–10 years and followed prospectively. For this study, analyses included participants with available resting-state fMRI and cognitive data at both baseline and the 2-year follow-up based on Release 4 and 5.
Recruitment	Participants were recruited across 21 sites in the United States using probability sampling strategies designed to approximate U.S. demographic distributions. Recruitment efforts included outreach through schools and community engagement. Potential selection bias is minimized by the large and demographically representative sample; however, limitations may still exist due to differential participation or attrition over time.<br><br>Written informed consent was obtained from parents and guardians, and assent was obtained from all participants. Participants were compensated for their participation as part of the ABCD study protocol.
Ethics oversight	Ethical approval was granted by the Institutional Review Board (IRB) at the University of California, San Diego, as well as by the IRBs of each participating study site.

Note that full information on the approval of the study protocol must also be provided in the manuscript.

Field-specific reporting

Please select the one below that is the best fit for your research. If you are not sure, read the appropriate sections before making your selection.

☐ Life sciences ☒ Behavioural & social sciences ☐ Ecological, evolutionary & environmental sciences

For a reference copy of the document with all sections, see nature.com/documents/nr-reporting-summary-flat.pdf

Behavioural & social sciences study design

All studies must disclose on these points even when the disclosure is negative.

Study description	This is a quantitative, longitudinal study examining the co-evolution of cognitive development and functional connectivity across childhood to adolescence. Analyses used resting-state fMRI and cognitive assessment data from two timepoints in the ABCD Study.
Research sample	The research sample was drawn from the ABCD Study, a large, demographically diverse cohort of U.S. children aged 9–10 years at baseline, designed to be broadly representative of the U.S. population. For this study, we included 2949 (female/male: 1447/1502) unrelated participants with usable resting-state fMRI and cognitive data at both baseline and 2-year follow-up after quality control (Table 1).

Sampling strategy	Participants were recruited into the ABCD Study using a probability-based sampling framework across 21 U.S. sites, designed to approximate the sociodemographic distribution of U.S. children aged 9–10 years. No formal a priori sample size calculation was performed for the present study. Instead, the analytic sample was determined by the number of participants in ABCD Releases 4 and 5 who met the inclusion criteria of having high-quality resting-state fMRI and complete cognitive data at both baseline and the 2-year follow-up. Specifically, after rs-fMRI quality control of both time points, 4615 participants remained. Excluding participants lacking cognition measures at both time points reduced the sample size to 3455, and further restricting to unrelated participants resulted in 3147 participants. Finally, we excluded participants who were scanned at different sites across the two time points and those from sites with fewer than 10 participants. Our final sample comprised 2949 individuals with data available at both timepoints. The sample size was considered sufficient to estimate longitudinal co-development of functional connectivity and cognition using mixed-effects and predictive modeling approaches.<br><br>In addition, a secondary subgroup analysis was conducted in a demographically matched subsample (N = 2,020), in which participants were matched on key demographic variables (e.g., age, sex, race/ethnicity and household income) as well as cognition to assess robustness and generalizability of the main findings. Further details are provided in the Methods.
Data collection	Data were collected through standardized assessments including paper-and-pencil tests, tablet-based cognitive tasks, interviews, and MRI scans. All procedures followed harmonized protocols across 21 sites in the United States. Detailed information on the data collection methodology is available at https://abcdstudy.org.
Timing	Data collection for the ABCD Study began in 2016 and is ongoing. For this study, baseline data were collected between September 2016 and October 2018. The 2-year follow-up data were collected beginning in 2018 and were included based on availability in ABCD data releases 4.0 and 5.0.
Data exclusions	Participants were excluded if they lacked usable resting-state fMRI (after quality control) or cognitive data at either timepoint.
Non-participation	This study used existing data from the ABCD Study. Information about non-participation and dropout is documented by the ABCD consortium.
Randomization	Participants were not allocated into experimental groups. All analyses were observational. Covariates such as age, and head motion were included in statistical models to adjust for potential confounding factors.

Reporting for specific materials, systems and methods

We require information from authors about some types of materials, experimental systems and methods used in many studies. Here, indicate whether each material, system or method listed is relevant to your study. If you are not sure if a list item applies to your research, read the appropriate section before selecting a response.

Materials & experimental systems

n/a	Involved in the study
☒	☐ Antibodies
☒	☐ Eukaryotic cell lines
☒	☐ Palaeontology and archaeology
☒	☐ Animals and other organisms
☒	☐ Clinical data
☒	☐ Dual use research of concern
☒	☐ Plants

Methods

n/a	Involved in the study
☒	☐ ChIP-seq
☒	☐ Flow cytometry
☐	☒ MRI-based neuroimaging

Plants

Seed stocks	*Report on the source of all seed stocks or other plant material used. If applicable, state the seed stock centre and catalogue number. If plant specimens were collected from the field, describe the collection location, date and sampling procedures.*
Novel plant genotypes	*Describe the methods by which all novel plant genotypes were produced. This includes those generated by transgenic approaches, gene editing, chemical/radiation-based mutagenesis and hybridization. For transgenic lines, describe the transformation method, the number of independent lines analyzed and the generation upon which experiments were performed. For gene-edited lines, describe the editor used, the endogenous sequence targeted for editing, the targeting guide RNA sequence (if applicable) and how the editor was applied.*
Authentication	*Describe any authentication procedures for each seed stock used or novel genotype generated. Describe any experiments used to assess the effect of a mutation and, where applicable, how potential secondary effects (e.g. second site T-DNA insertions, mosiacism, off-target gene editing) were examined.*

Magnetic resonance imaging

Experimental design

Design type	resting-state fMRI
Design specifications	Each participant underwent four runs of resting-state fMRI, each lasting 5 minutes, for a total of 20 minutes of functional data per session. During acquisition, participants kept their eyes open and passively viewed a fixation cross.
Behavioral performance measures	We only used behavioral measures that were collected outside of the scanner.

Acquisition

Imaging type(s)	functional
Field strength	3T
Sequence & imaging parameters	Data were acquired across multiple scanner platforms, leading to some variability in sequence parameters. However, all resting-state fMRI scans were collected with 2.4 mm isotropic resolution and a repetition time (TR) of 800 ms. Details can be found here: https://abcdstudy.org/images/Protocol_Imaging_Sequences.pdf
Area of acquisition	Whole brain scan

Diffusion MRI ☐ Used ☒ Not used

Preprocessing

Preprocessing software	FreeSurfer (7.4.1); FSL (5.0.10); The full preprocessing pipeline is publicly available at https://github.com/ThomasYeoLab/Standalone_CBIG_fMRI_Preproc2016, archived at https://doi.org/10.5281/zenodo.19723245. Study-specific preprocessing scripts are available at https://github.com/ThomasYeoLab/ABCD_scripts, archived at https://doi.org/10.5281/zenodo.19723255.
Normalization	Functional data were aligned to each participant's T1-weighted anatomical image using boundary-based registration (Greve and Fischl, 2009, NeuroImage) implemented in FreeSurfer's bbregister. Cortical surfaces were reconstructed from the T1 images and registered to the FreeSurfer fsaverage6 surface space using FreeSurfer.
Normalization template	FreeSurfer fsaverage6 surface space.
Noise and artifact removal	Nuisance regression was performed using 18 regressors: the global signal, six motion correction parameters, averaged ventricular signal, averaged white matter signal, and their temporal derivatives.
Volume censoring	Volumes with framewise displacement > 0.3 mm (Jenkinson et al., 2002, NeuroImage) or voxel-wise differentiated signal variance (DVARS) > 50 (Power et al., 2012, NeuroImage), along with one preceding and two subsequent volumes, were marked as outliers and censored.

Statistical modeling & inference

Model type and settings	We used two types of models: (1)Mass univariate linear mixed-effects models were used to examine longitudinal changes in resting-state functional connectivity (FC) at the edge level. Site effects were harmonized using longitudinal ComBat prior to model fitting in this analysis. (2) multivariate predictive analyses were used to explore the relationship between FC and cognition (i.e., FCY0->CogY0; FCY2->CogY2; FCY0->CogY2; FCchange->CogY2; FCY0→CogChange; FCchange->CogChange).
Effect(s) tested	(1) Using mass univariate modeling, we tested whether resting-state functional connectivity changed longitudinally at the group level. (2) In multivariate predictive analyses, we evaluated whether the predictive models described above achieved statistically significant performance. (3) We additionally applied corrected resampled t-tests to compare predictive performance across models.

Specify type of analysis: ☒ Whole brain ☐ ROI-based ☐ Both

Statistic type for inference (See Eklund et al. 2016)	We used whole-brain resting-state functional connectivity, and no voxel-wise or cluster-wise inference was performed. Instead, we evaluated model performance using summary-level statistics: Pearson correlation between predicted and observed cognitive scores.
Correction	False Discovery Rate

Models & analysis

n/a	Involved in the study
☐	☒ Functional and/or effective connectivity
☒	☐ Graph analysis
☐	☒ Multivariate modeling or predictive analysis

Functional and/or effective connectivity

Pearson correlation

Multivariate modeling and predictive analysis

We used kernel ridge regression to perform the prediction analysis (i.e., FCY0->CogY0; FCY2->CogY2; FCY0->CogY2; FCchange->CogY2; FCY0→CogChange; FCchange->CogChange). No additional dimensionality reduction was applied. Models were trained and evaluated using nested cross-validation, and performance was assessed using Pearson correlation between predicted and observed scores.
