# Supplementary material for: Convergent and divergent brain–cognition development in early adolescence
Source: Nat Commun. 2026 May 26;17:6868. doi: 10.1038/s41467-026-73668-y (PMC13388698; doi:10.1038/s41467-026-73668-y)
Supplement: Supplementary file 3 — Transparent Peer Review file [file 41467_2026_73668_MOESM3_ESM.pdf]

nature portfolio

Peer Review File

Convergent and divergent brain–cognition development in early adolescence

Corresponding Author: Dr B.T. Thomas Yeo

This file contains all reviewer reports in order by version, followed by all author rebuttals in order by version.

Version 0:

Reviewer comments:

Reviewer #1

(Remarks to the Author)
The reviewed manuscript addresses question how the relation between human brain properties and cognitive abilities change during development. They analyzed 2949 adolescence from the ABCD project and focused their perspective in functional brain connectivity. The results suggest that intrinsic (resting-state) connectivity more strongly predicts future cognitive ability than cognitive ability assessed during the same time. Further, the brain-cognition relationship seem to strengthen over time, but neither baseline connectivity nor connectivity changes could predict longitudinal change in cognitive ability.

Overall, the manuscript is written in good scientific English and the Figures are great. The Introduction presents important empirical background, the Methods are advanced, analyses were conducted thoroughly and the Results are very interesting. Also the research question in general is relevant to a broader audience, although the very restricted age range and missing independent replication are limitations.

Therefore I have only Minor comments - more suggestions:

1. The Introduction is mainly focused on the divergent vs. congruent question, while this is neither the main aim of the study nor the main result. I would acknowledge a more balanced Introduction. E.g., articles from working groups of Aron Barbey (e.g., Barbey, 2018) or Kirsten Hilger (e.g., Thiele et al. (2025)) might inform the current study.

2. Table 1 would benefit from adding z-scores.

3. Figure 1c seem to be not referenced in the text.

4. Line 130: The "but" is misleading.

5. Line 132: Is misleading as can be understood differently. Maybe choose a different wording. Same refers to line 136/137.

6. Nearly exact wording is present at the end of the Introduction, begin of Discussion and in the Abstract - nearly complete same phrasing.

7. Potentially interesting post-hoc analysis: Can FC from time point 2 predict cognitive ability from time point 1? (as control)

8. Line 327: A r seem to be missing before the correlation value?

9. Would Figure 4 profit from an aggregated comparison between within-network connections vs. between-network connections?

Overall, the study is sound and interesting and could be considered for publication in Nature Communications. I wish the authors good luck with the revision and looking forward to see this study published irrespective the question in which journal.

(Remarks on code availability)

Reviewer #2

(Remarks to the Author)

This work presents a novel approach to connectivity analysis in understanding changes associated with development. The work presents evidence for both convergent and divergent changes in connectivity associated with both cognition level and change in cognition (chiefly, the first principal component of 7 different cognitive test scores) across two time points in the ABCD data.

The paper is well written, and a substantial amount of data is included in developing the models. Overall, the methodology is considered rigorous.

I find the treatment of motion and sex appears to be under developed.

1. First for motion it would be good if the raw mean and standard deviation for frame displacement per subject was shown as a function of PC1, and the 7 test scores. It would also be good to know to what extent there are time1 to time2 motion changes – this is not explicitly outlined but all this information could be included in Table 1. While it is acknowledged that motion is regressed out this does not completely relieve the effects of motion on the data, and it is important to know to what extent motion is correlated with the primary variables of interest.

2. Similarly, sex is a variable that is regressed out of the data but it is well known that this is not simple a linear factor that can be removed, and furthermore there is evidence that the different sexes may indeed require different models. Finally in this age range considered the development rates for females vs males can be quite different and thus the mostly null result for individual differences in connectivity or cognitive variables may look quite different when the sexes are treated separately. I think the work should be repeated on males and females separately in addition to this group data.

Minor clarifications
Line 247 – what fold for nested cross-validation? It would be good to outline this here – I realize it’s 3 sites left out etc but this could be somewhat clarified here.

Line 253 - Just predicting PC1. Is there not more to learn with respect to the predictive powers for the 7 scores? This seems to be under treated and a lot more could be done with the data. The predictive powers shown in the supplementary section are mostly quite high.

Line 262 – Here it is stated that at the later age there may be enhanced relationships between brain and behavior but this is why we need the motion data – it could also just be better MRI data because there is less motion as the kids get older and gain experience. What’s the motion difference by release?

(Remarks on code availability)

Reviewer #3

(Remarks to the Author)
In the current work, Xie et al investigated concurrent and predictive relationships between 10-12-year-olds brain functional connectivity and cognitive outcomes. 2949 subjects from the ABCD project were used in this investigation. The results revealed that baseline FC correlates with baseline cognition better than baseline cognition does. Baseline FC predicted Yr2 cognition but not as good as Yr2 FC predicted Yr2 cognition. When applying the baseline FC-cognition prediction model to Yr2 FC, it predicted Yr2 cognition better than baseline cognition. Finally, Baseline FC is better at predicting Yr2 cognition than longitudinal changes do, even after accounting for reliability differences. Strengths of this work include large sample size from the well-curated ABCD data, solid methodological selection and implementation, and careful comparisons of different prediction models. I do have some concerns on the generalizability of the very specific findings and claims.

1. It is well known that missing data in ABCD is not necessarily random and the odds of a participant's missing data is related to demographic, behavioral, and/or clinical variables. In this project, only 2949 subjects were selected from >10000 datasets with strict inclusion criteria of having good quality MRIs at two consecutive time points as well as complete cognitive measures, the potential bias associated with this exclusion practice can not be overlooked and whether the conclusion is generalizable is questionable.
2. The relationships were also only examined across a single 2-year span between 10 and 12 years of age. Whether the conclusion generalizes to either shorter or longer age spans, or to any other age periods is hard to know. This can hardly justify the very general title of "Convergent and Divergent Brain-Cognition Development". Actually, the hypothesis of "convergent and divergent" relationships was only tested in Figure 7 and related results were only represented in a small fraction of the discussion, so the title seems to be a bit off in covering the main findings of the study.
3. Finally, boys and girls are known to develop differently so how about examining these relationships in each sex group?

(Remarks on code availability)

Version 1:

Reviewer comments:

Reviewer #1

(Remarks to the Author)
The authors have addressed all my concerns in sufficient detail and I wish the authors good luck with this paper!

(Remarks on code availability)

Reviewer #2

(Remarks to the Author)
The authors have sufficiently responded to my comments and I recommend this for publication now. Nice work.

(Remarks on code availability)

Reviewer #3

(Remarks to the Author)
The authors have successfully addressed my concerns.

(Remarks on code availability)

Open Access This Peer Review File is licensed under a Creative Commons Attribution 4.0 International License, which permits use, sharing, adaptation, distribution and reproduction in any medium or format, as long as you give appropriate credit to the original author(s) and the source, provide a link to the Creative Commons license, and indicate if changes were made.
In cases where reviewers are anonymous, credit should be given to 'Anonymous Referee' and the source.
The images or other third party material in this Peer Review File are included in the article's Creative Commons license, unless indicated otherwise in a credit line to the material. If material is not included in the article's Creative Commons license and your intended use is not permitted by statutory regulation or exceeds the permitted use, you will need to obtain permission directly from the copyright holder.
To view a copy of this license, visit https://creativecommons.org/licenses/by/4.0/

Reply to reviewers NCOMMS-25-48986A (Convergent and Divergent Brain–Cognition Development)

We were pleased by the reviewers' interest in our work and thank them for their careful reading of the manuscript and their thoughtful, constructive comments. The reviewers provided several important suggestions, all of which we have carefully considered and addressed in our revision. Below, we provide point-by-point responses to each comment (in blue), with the reviewers' comments reproduced in italics. For clarity, relevant changes to the manuscript are quoted verbatim in normal font where appropriate. We believe that these revisions have substantially strengthened the manuscript and hope that it is now suitable for publication.

Reviewer #1:

R1Q1. The reviewed manuscript addresses question how the relation between human brain properties and cognitive abilities change during development. They analyzed 2949 adolescence from the ABCD project and focused their perspective in functional brain connectivity. The results suggest that intrinsic (resting-state) connectivity more strongly predicts future cognitive ability than cognitive ability assessed during the same time. Further, the brain-cognition relationship seem to strengthen over time, but neither baseline connectivity nor connectivity changes could predict longitudinal change in cognitive ability.

Overall, the manuscript is written in good scientific English and the Figures are great. The Introduction presents important empirical background, the Methods are advanced, analyses were conducted thoroughly and the Results are very interesting. Also the research question in general is relevant to a broader audience, although the very restricted age range and missing independent replication are limitations. Therefore I have only Minor comments - more suggestions:

Response: We thank the reviewer for the positive comments. We have addressed your concerns below.

R1Q2. The Introduction is mainly focused on the divergent vs. congruent question, while this is neither the main aim of the study nor the main result. I would acknowledge a more balanced Introduction. E.g., articles from working groups of Aron Barbey (e.g., Barbey, 2018) or Kirsten Hilger (e.g., Thiele et al. (2025)) might inform the current study.

Response: We thank the reviewer for raising this point. We agree with the reviewer that the previous introduction placed disproportionate emphasis on "feature-level" convergence and divergence of cross-sectional and longitudinal predictive models, which was only explored in the final section of the study.

We have now re-worked the introduction to also discuss how "predictive power" might also be convergent or divergent between cross-sectional and longitudinal predictive models. More specifically, baseline connectivity might be predictive of baseline cognition. However, longitudinal change in connectivity may or may not be predictive of future cognitive ability or longitudinal change in cognitive ability.

As suggested, we have also incorporated perspectives from Barbey and recent work from Thiele in our second paragraph of introduction to emphasize the network basis of cognitive ability.

We have updated the introduction as follows:

page 3-4 (Introduction)

> Resting-state fMRI (rs-fMRI) is a powerful tool for examining functional brain network organization[5-8]. Contemporary network neuroscience theories propose that individual differences in cognitive ability arise from corresponding differences in brain network architecture[9]. Consistent with this framework, previous studies have developed cross-sectional brain–cognition models that can predict individual-level cognitive performance in children, adolescents, and young adults based on resting-state functional connectivity (FC)[10-18]. While valuable, these cross-sectional models reflect a single snapshot of brain–cognition relationships, neglecting dynamic changes that can only be revealed in longitudinal designs[19-22].
>
> Conceptually, cross-sectional analyses reveal between-individual traits, which might differ from dynamic within-individual changes captured by longitudinal designs[23,24]. Figure 1a illustrates the classical example of how cross-sectional (between-individual) and longitudinal (within-individual) estimates can diverge for a single phenotype, a phenomenon known as the Simpson's paradox[25-27]. When extended to predictive models of brain and cognition, the situation becomes more complex. For example, salience network FC may robustly predict cognitive ability cross-sectionally across individuals (Figure 1b). However, longitudinal change in salience network FC might not be predictive of future cognitive ability or longitudinal change in cognitive ability (Figure 1b).
>
> Beyond predictive power, network features supporting prediction might also differ between cross-sectional and longitudinal models. For example, stronger salience network FC predicted better cognitive ability at the individual level[11]. If this relationship reflects a causal mechanism, one would expect that children with greater increases in salience network FC over time would also show greater cognitive improvement (Figure 1c). However, cross-sectional and longitudinal models may not always align: it is also possible that children with larger reductions in salience network FC exhibit the most cognitive gains (Figure 1d). To put it in another way, suppose Luca is a cognitively advanced child whose brain connectivity patterns differ from his peers. As his peers grow older and become more cognitively capable, do their brains become more like Luca's? If so, this would reflect feature-level convergence between cross-sectional and longitudinal estimates (Figure 1c). If not, this would indicate feature-level divergence (Figure 1d).

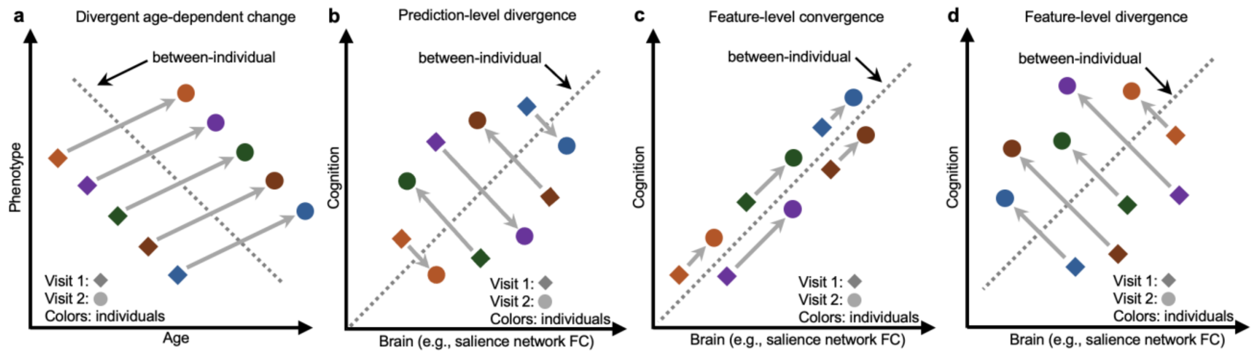

Figure 1. Illustration of how cross-sectional and longitudinal estimates can converge or diverge. (a) Classical (Simpson's paradox) illustration, where within-individual changes (arrows) diverge from between-individual differences (dashed line). (b) Predictive power divergence between cross-sectional and longitudinal predictive models. In this example, salience network FC yields strong cross-sectional prediction of cognitive ability (dotted line). However, longitudinal within-individual change in salience network connectivity is not predictive of cognitive ability at visit 2, as well as longitudinal cognitive change (gray arrows). (c) Feature-level convergence between cross-sectional and longitudinal models. For example, greater salience network connectivity predicts better cognitive ability among children cross-sectionally (dashed line). Assuming a causal relationship, individuals with larger increases in salience network FC should enjoy greater cognitive gains longitudinally (arrows). (d) Feature-level divergence between cross-sectional and longitudinal models. Similar to panel (c), greater salience network connectivity predicts better cognitive ability among children cross-sectionally (dashed line). However, individuals with larger reductions in salience network FC enjoy greater cognitive gains longitudinally (arrows). Figure S1 illustrates other possible divergences between cross-sectional and longitudinal estimates of brain–cognition relationship. We hypothesize that convergence and divergence vary across brain networks.

R1Q3. Table 1 would benefit from adding z-scores.

Response: Following the reviewer's recommendation, we now standardize the longitudinal change for each cognitive measure using its baseline standard deviation. This places all change scores on a common, interpretable standard-deviation scale. In the revised manuscript, we have added a new column reporting the standardized longitudinal change in **Table 1**.

Table 1. Age, motion, and cognitive measures at baseline, Year 2, and their longitudinal change.

	Baseline	Year 2	Longitudinal change (raw/standardized)	
Participants (F/M)	2949 (1447/1502)	Same as baseline	N.A.	
Age (years)	9.93 (0.62)	11.94 (0.65)	2.01 (0.16)	N.A.
Mean FD (mm)	0.07 (0.04)	0.06 (0.03)	-0.01 (0.04)	-0.25 (1.03)
Std FD (mm)	0.12 (0.09)	0.09 (0.08)	-0.03 (0.10)	-0.28 (1.08)
RAVLT	69.93 (14.96)	69.59 (13.95)	-0.35 (13)	-0.02 (0.87)

LMT	0.61 (0.17)	0.75 (0.18)	0.14 (0.18)	0.82 (1.02)
PicVocab	85.54 (7.97)	89.73 (8.39)	4.19 (6.14)	0.53 (0.77)
Flanker	94.97 (8.33)	100.58 (7.13)	5.61 (8.45)	0.67 (1.01)
Pattern	89.06 (14.06)	104.15 (14.45)	15.09 (14.73)	1.07 (1.05)
Picture	104.15 (12.05)	110.16 (11.93)	6.01 (12.61)	0.5 (1.05)
Reading	91.72 (6.81)	95.42 (6.65)	3.70 (4.74)	0.54 (0.7)
PC1	0.43 (1.55)	1.86 (1.55)	1.43 (0.95)	0.92 (0.61)

Data are shown for 2949 participants (1447 female/1502 male). Values are presented as mean (standard deviation). Raw longitudinal change was computed as Year 2 minus baseline. Standardized longitudinal change was obtained by dividing the raw change by the baseline standard deviation of each measure, enabling comparison of change magnitudes across cognitive measures. Mean FD: mean framewise displacement across volumes. Std FD: standard deviation of framewise displacement across volumes. RAVLT: Rey Auditory Verbal Learning Test (verbal memory); LMT: Little Man Task (spatial reasoning); PicVocab: Picture Vocabulary Task (vocabulary); Flanker: Flanker Task (executive function); Pattern: Pattern Comparison Processing Speed Test (processing speed); Picture: Picture Sequence Memory Test (episodic memory); Reading: Oral Reading Recognition Task (reading ability). PC1: the first principal component of the above seven cognitive measures. Consistent with previous work[31], we used uncorrected standard scores for each NIH Toolbox task, total correct scores for the RAVLT, and percent correct scores for the LMT. For all cognitive measures, greater value indicates better performance. See more detailed explanation of cognitive measures in Methods.

For consistency, we applied the same standardization when summarizing inter-individual variability in longitudinal change in Figures 2e and 2f. We also replaced the original bar plots with violin plots to better illustrate the distribution of standardized longitudinal change. This approach ensures that variability is expressed in the same standardized units across all cognitive measures.

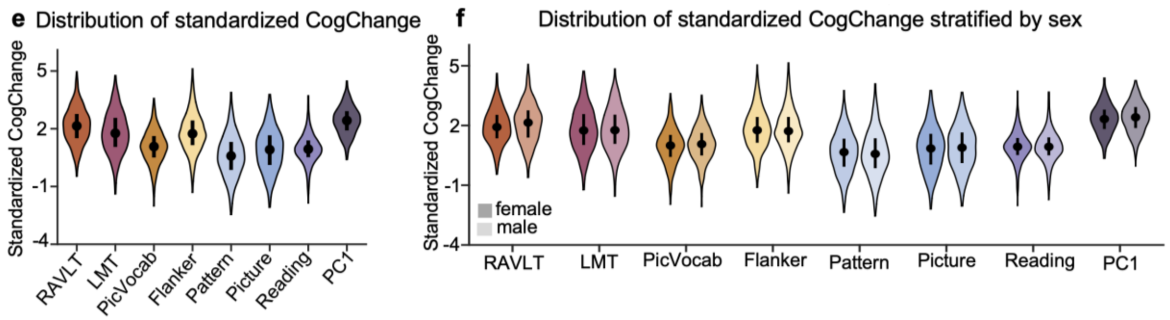

Figure 2. (e) Individual variability in standardized longitudinal cognitive change. For each individual and each cognitive measure, cognition change was defined as the difference in scores between the two timepoints. Sex, baseline age and age interval (between baseline and Year 2) were regressed out. The residual change scores were then standardized by the baseline standard deviation of each measure. Violin plots show the distribution of standardized cognitive change, with dots indicating the median and bars indicating the interquartile range (25th–75th percentile). (f) Individual variability in standardized longitudinal cognitive change stratified by sex.

R1Q4. Figure 1c seem to be not referenced in the text.

Response: We thank the reviewer for pointing this out. Note that Figure 1c is now Figure 1d in the revised manuscript (see our response to R1Q2). All panels in Figure 1 are now explicitly referenced in the main text (page 3, lines 86, 91, 99, and 101).

R1Q5. Line 130: The "but" is misleading.

Response: We agree with the reviewer. In the original sentence, the use of "but" incorrectly implied a contradiction between "changing more over time" and "being less stable across individuals." In this case, "and" would have been the more appropriate conjunction. However, we note that this entire sentence has been removed as part of our revision of the final paragraph of the introduction in response to R1Q7. The revised text no longer contains the phrasing flagged by the reviewer.

R1Q6. Line 132: Is misleading as can be understood differently. Maybe choose a different wording. Same refers to line 136/137.

Response: We agree with the reviewer. However. since we have completely changed the last paragraph of the introduction (see R1Q7), this ambiguity is no longer there.

R1Q7. Nearly exact wording is present at the end of the Introduction, begin of Discussion and in the Abstract - nearly complete same phrasing.

Response: Thank you for pointing this out. We have revised the introduction and discussion to avoid the repetitive phrasing. For context, we have also included the abstract for your convenience.

page 2 (Abstract)

How functional brain networks and cognition co-evolve during adolescent development remains poorly understood. Using resting-state functional MRI (rs-fMRI) and cognitive data at baseline and Year 2 collected from 2,949 individuals in the Adolescent Brain Cognitive Development (ABCD) Study, we examine how individual-level brain–cognition predictive models develop longitudinally in early adolescence. We find that baseline resting-state functional connectivity (FC) more strongly predicts future cognitive ability than baseline cognitive ability. Models trained on baseline FC to predict baseline cognition generalize better to Year 2 FC and cognition, suggesting that brain–cognition relationships strengthen over time. Intriguingly, baseline FC outperforms longitudinal FC change in predicting future cognitive ability. One potential reason is the lower reliability of FC change compared to baseline FC: ICC = 0.24 vs. 0.56. However, reducing baseline FC's reliability by shortening scan duration only partially narrows the predictive gap, suggesting reliability alone cannot be the full explanation. Furthermore, neither baseline FC nor FC change meaningfully predicts longitudinal change in cognitive ability. We also identify converging and diverging predictive network features when comparing cross-sectional and longitudinal models of brain–cognition relationships, uncovering a

multivariate twist on Simpson's paradox. Notably, these convergences and divergences exhibit clear sex-specific patterns. Together, these findings suggest that during early adolescence, stable individual differences in brain functional network organization play a more critical role than dynamic changes in shaping future cognitive outcomes.

page 4 (Introduction)

In this study, we examined how FC and cognition co-evolve during early adolescence using longitudinal rs-fMRI and cognitive data collected at baseline and Year 2 from 2949 participants of the ABCD Study[28-30]. We began by quantifying stability and change in cognition and FC across the two timepoints to characterize the inter-individual variability landscape that frames subsequent analyses of brain–cognition relationships. We then assessed the strength of cross-sectional FC–cognition relationships at each timepoint and evaluated how these associations evolve over time. Next, we evaluated longitudinal FC–cognition relationships by testing whether FC change predicted cognition at Year 2 and whether it predicted cognitive change across the two timepoints. We also repeated all analysis separately in males and females to explore potential sex differences. Together, these analyses provide an integrated framework for understanding how stable functional network architecture and short-term network changes relate to cognitive outcomes.

page 20 (Discussion)

Longitudinal analyses of ABCD rs-fMRI and cognitive data show that, while cognitive performance improves on average over time, individual differences in cognition remain highly stable. FC exhibits both developmental change and individual stability that are organized along the sensory–association (S-A) axis. Baseline FC predicts future cognitive ability better than it predicts baseline cognitive ability. Prediction accuracy also improves when models trained on baseline data are applied to Year 2 FC and cognition. These results suggest that brain–cognition relationships become more pronounced during early adolescence. In addition, baseline FC surpasses longitudinal FC change in predicting future cognitive ability. Both baseline FC and FC change cannot predict cognitive change well. Beyond differences in predictive power, we also found both overlapping and distinct network predictors across cross-sectional and longitudinal predictive models. Overall, these findings indicate that early adolescent brain–cognition development is driven more by stable individual differences in network organization than by short-term connectivity changes.

R1Q8. Potentially interesting post-hoc analysis: Can FC from time point 2 predict cognitive ability from time point 1? (as control)

Response: We thank the reviewer for this suggestion. In the revised manuscript, we have performed the proposed control analysis using Year 2 FC to predict cognition at baseline (FCY2→CogY0). As expected, the prediction accuracies were significant for all 8 cognitive measures (FDR $q < 0.05$). Importantly, the prediction accuracies (FCY2→CogY0) were similar to FCY0→CogY0, and worse than FCY2→CogY2, suggesting that the improvement in predicting Year 2 cognition is not due to better fMRI quality in Year 2.

page 12 (Results)

In addition, we also used Year 2 FC to predict baseline cognitive ability (FCY2→CogY0). If the higher prediction accuracy at Year 2 were primarily driven by lower motion or improved fMRI quality, then FCY2 should outperform FCY0 in predicting CogY0. However, prediction accuracies of FCY2→CogY0 were similar to the baseline model (FCY0→CogY0), but lower than prediction of Year 2 cognition (FCY2→CogY2) across all cognitive measures (Table S3).

Table S3. Prediction accuracy for each cognitive measure and each cross-sectional model

	FCY0-CogY0	FCY0-CogY2	FCY2-CogY2	ModelTransfer	FCY2-CogY0
RAVLT	0.28	0.27	0.30	0.31	0.28
LMT	0.20	0.25	0.26	0.25	0.22
PicVocab	0.42	0.45	0.47	0.46	0.42
Flanker	0.14	0.20	0.19	0.17	0.12
Pattern	0.08	0.12	0.15	0.12	0.10
Picture	0.19	0.19	0.24	0.25	0.18
Reading	0.35	0.39	0.41	0.39	0.36
PC1	0.43	0.46	0.49	0.48	0.44

R1Q9. Line 327: A r seem to be missing before the correlation value?

Response: We have added the missing "r":

page 12 (Results)

However, the best prediction accuracy was only r = 0.13 (for PC1).

R1Q10. Would Figure 4 profit from an aggregated comparison between within-network connections vs. between-network connections?

Response: We thank the reviewer for this suggestion. To provide an aggregated comparison of within-network and between-network connections for Figure 4, we averaged the absolute Haufe-transformed weights within each network and between networks. This analysis revealed that within-network connections consistently showed larger contributions than between-network connections for all three models (Figure S5). We have updated the manuscript as follows:

page 10 (Results)

For all three models, within-network FC contributed more towards prediction performance than between-network FC (Figure S5).

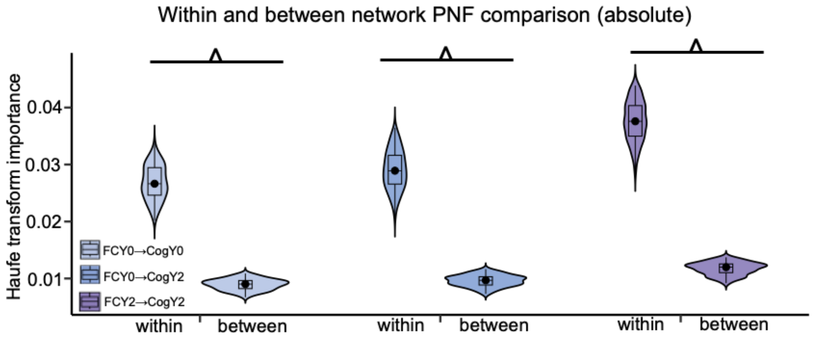

Figure S5. Within-network versus between-network predictive network feature (PNF) contributions for each model. For each cross-validation fold, mean within-network and between-network PNF magnitudes (absolute values) were computed separately for each model. Each violin plot contains 120 data points, corresponding to the 120 repetitions of cross-validation. Carets (^) indicate statistically significant within-network versus between-network differences based on the corrected resampled t-test (FDR $q < 0.05$).

Overall, the study is sound and interesting and could be considered for publication in Nature Communications. I wish the authors good luck with the revision and looking forward to see this study published irrespective the question in which journal.

Response: We thank the reviewer again for their positive and encouraging assessment of our work.

Reviewer #2:

R2Q1. This work presents a novel approach to connectivity analysis in understanding changes associated with development. The work presents evidence for both convergent and divergent changes in connectivity associated with both cognition level and change in cognition (chiefly, the first principal component of 7 different cognitive test scores) across two time points in the ABCD data.

The paper is well written, and a substantial amount of data is included in developing the models. Overall, the methodology is considered rigorous.

I find the treatment of motion and sex appears to be under developed.

Response: We would like to thank the reviewer for the positive comments and important suggestions.

R2Q2. First for motion it would be good if the raw mean and standard deviation for frame displacement per subject was shown as a function of PC1, and the 7 test scores. It would also be good to know to what extent there are time1 to time2 motion changes – this is not explicitly outlined but all this information could be included in Table 1. While it is acknowledged that motion is regressed out this does not completely relieve the effects of motion on the data, and it is important to know to what extent motion is correlated with the primary variables of interest.

Response: We have added the raw mean and standard deviation of framewise displacement (mean FD and std FD) to Table 1 (see response to R1Q3). As expected, both measures showed small mean longitudinal decreases (raw change: −0.01 for mean FD and −0.03 for std FD).

To formally evaluate motion changes from baseline to Year 2, we fitted linear mixed-effects models to estimate the effect of within-participant age change, accounting for baseline age, sex, site, and participant-specific random intercepts (see SI Methods below). Both mean FD ($t = -12.9$, $p = 1.4\times10^{-37}$) and std FD ($t = -13.9$, $p = 1.6\times10^{-43}$) exhibit significant longitudinal decrease, suggesting reduced average head motion (mean FD) and more stable, less variable head motion (std FD) at Year 2.

To assess whether motion is systematically related to the cognitive measures of interest, we fitted separate linear mixed-effects models for each cognitive measure. Consistent with previous studies, all cognitive measures (except LMT and Picture Vocabulary Task) were significantly associated with mean FD (Table S2, FDR $q < 0.05$). All cognitive measures (except Picture Vocabulary Task) were significantly associated with std FD (Table S2, FDR $q < 0.05$). Therefore, children with higher cognitive scores have lower head motion (mean FD) and less variable head motion (std FD).

We have updated the manuscript as follows. We note that control analyses involving head motion can be found in our response to R2Q6.

page 12 (Results)

We note that head motion and head motion variability decreased with age (Table 1), as reflected by reductions in both mean framewise displacement (FD) and the standard deviation of FD (mean FD: $t = -12.9$, $p = 1.4 \times 10^{-37}$; std FD: $t = -13.9$, $p = 1.6 \times 10^{-43}$). Higher cognitive ability was also associated with lower average head motion and reduced motion variability across participants (Table S2).

Table S2. Linear mixed-effects model results for relationship between motion and cognition

	mean FD				std FD			
	β	SE	t	p	β	SE	t	p
RAVLT	-0.00008	0.00003	-2.48	**0.01306**	-0.00019	0.00008	-2.34	**0.01931**
LMT	-0.00450	0.00275	-1.64	0.10130	-0.01614	0.00659	-2.45	**0.01436**
PicVocab	-0.00012	0.00006	-1.97	0.04917	-0.00028	0.00015	-1.88	0.05994
Flanker	-0.00013	0.00006	-2.07	**0.03848**	-0.00033	0.00015	-2.29	**0.02209**
Pattern	-0.00010	0.00003	-3.11	**0.00187**	-0.00031	0.00008	-3.80	**0.00015**
Picture	-0.00011	0.00004	-2.89	**0.00384**	-0.00028	0.00009	-2.95	**0.00322**
Reading	-0.00017	0.00008	-2.28	**0.02282**	-0.00037	0.00018	-2.05	**0.04047**
PC1	-0.00141	0.00034	-4.11	**0.00004**	-0.00354	0.00081	-4.36	**0.00001**

Note that bold p-values denote effects that remained significant after false discovery rate (FDR) correction at $q < 0.05$.

page 5 (SI Methods)

S2. Relationships between head motion and age

To evaluate motion changes from baseline to Year 2, we applied the following linear mixed-effects model to estimate the effect of within-participant age change, accounting for baseline age, sex, site, and participant-specific random intercepts:

$$\text{Motion}_{it} = \beta_0 + \beta_1 \text{Age}_{i1} + \beta_2 (\text{Age}_{it} - \text{Age}_{i1}) + \beta_3 \text{Sex}_{it} + \beta_4 \text{SiteID}_{it} + u_i + \varepsilon_{it} \quad (4),$$

Where *i* indexes the *i*-th participant, *t* references the time point. u_i is the random intercept and ε_{it} is the noise term. Mean framewise displacement (FD) and std FD are the dependent variables.

S3. Relationships between head motion and cognition

To assess whether motion is systematically related to the cognitive measures of interest, we fit an additional linear mixed effect model:

$$\text{Motion}_{it} = \beta_0 + \beta_1 \text{Age}_{i1} + \beta_2 (\text{Age}_{it} - \text{Age}_{i1}) + \beta_3 \text{Sex}_{it} + \beta_4 \text{Cognition}_{it} + \beta_5 \text{SiteID}_{it} + u_i + \varepsilon_{it} \quad (5),$$

Where *i* indexes the *i*-th participant, t references the time point. u_i is the random intercept and ε_{it} is the noise term. Mean FD and std FD are the dependent variables. We applied this model to all the eight cognitive measures.

R2Q3. Similarly, sex is a variable that is regressed out of the data but it is well known that this is not simple a linear factor that can be removed, and furthermore there is evidence that the different sexes may indeed require different models. Finally in this age range considered the development rates for females vs males can be quite different and thus the mostly null result for individual differences in connectivity or cognitive variables may look quite different when the sexes are treated separately. I think the work should be repeated on males and females separately in addition to this group data.

Response: We thank the reviewer for this excellent suggestion. We agree that males and females likely exhibit distinct developmental patterns in both FC and cognitive ability. We have therefore repeated multiple analyses separately in males and females to explicitly examine sex-specific patterns and assessed whether sex differences are present.

(1) Sex differences in longitudinal cognitive changes. For both males and females, cognitive abilities at baseline and Year 2 were positively correlated (i.e., cognitively stable), consistent with the result of the full sample combining males and females. There was no sex difference in terms of cognitive stability for all cognitive measures. Longitudinal changes were also similar for males and females, with the exception of the Rey Auditory Verbal Learning Test (RAVLT), where males showed improvement over time, while females exhibited a slight decline. Sex differences in the magnitude of individual variability in cognitive changes were also observed for RAVLT and Pattern Comparison Processing Speed Test. We have updated the manuscript as follows:

pages 5-6 (Results)

> Cognitive ability at baseline and Year 2 was positively correlated, suggesting that individuals with higher baseline cognition generally maintained their cognitive advantage over their peers at Year 2 (Figure 2a). No significant sex differences were observed in cognitive stability (Figure 2b; Figure S2).
>
> At the group level, cognitive performance improved from baseline to Year 2 across most measures (Figure 2c), with the notable exception of the Rey Auditory Verbal Learning Test (RAVLT). A mixed-effects model revealed a significant sex-by-age interval interaction for RAVLT ($t = -4.41$, FDR $q < 0.05$; Table S1), with males showing improvement over time and females exhibiting a slight decline (Figure 2d). Consequently, there was no improvement in RAVLT for the full sample (Figure 2c). In addition, males demonstrated slightly greater rate of longitudinal improvement than females in overall cognition (PC1; $t = -2.40$, FDR $q < 0.05$; Figure 2d).
>
> Beyond group-level changes, there was substantial individual variability in longitudinal cognitive change across most cognitive measures (Figure 2e), and within each sex (Figure 2f). Sex differences in the magnitude of individual variability were observed for RAVLT and Pattern Comparison Processing Speed Test, with males exhibiting greater variability than females for both measures (FDR $q < 0.05$).

page 30 (Methods)

Sex-stratified and sex-difference analyses
To assess sex-specific effects, all analyses were additionally repeated separately in females and males. Statistical tests were performed to compare the sexes. Detailed descriptions of sex-stratified and sex-comparison procedures are provided in the Supplementary Methods.

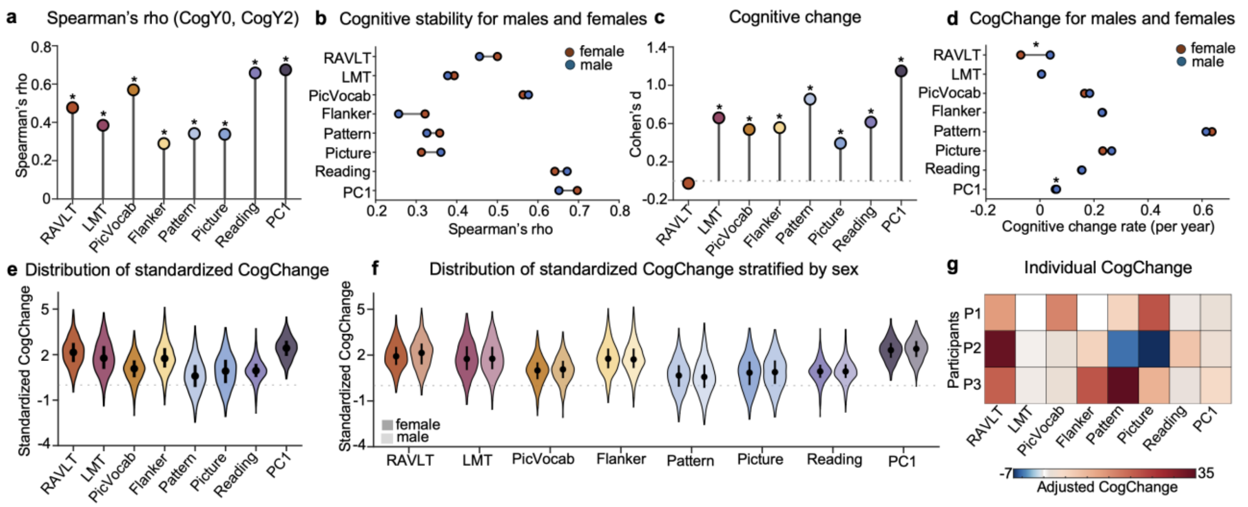

Figure 2. Individual differences in longitudinal cognitive change in early adolescence. (a) Spearman's correlation between baseline and Year 2 cognitive measures (i.e., cognitive stability). The positive correlations indicate that children with higher baseline cognition generally maintained their cognitive advantage over their peers at Year 2. (b) Spearman's correlation between baseline and Year 2 cognitive measures (i.e., cognitive stability) stratified by sex. (c) Longitudinal cognitive change at the group level estimated from a linear mixed effects model. (d) Cognitive change rate for males and females estimated from a linear mixed effects model. (e) Individual variability in standardized longitudinal cognitive change. For each individual and each cognitive measure, cognition change was defined as the difference in scores between the two timepoints. Sex, baseline age and age interval (between baseline and Year 2) were regressed out. The residual change scores were then standardized by the baseline standard deviation of each measure. Violin plots show the distribution of standardized cognitive change, with dots indicating the median and bars indicating the interquartile range (25th–75th percentile). (f) Individual variability in standardized longitudinal cognitive change stratified by sex. (g) Longitudinal changes in the eight cognitive measures for three participants. RAVLT: Rey Auditory Verbal Learning Test (verbal memory); LMT: Little Man Task (spatial reasoning); PicVocab: Picture Vocabulary Task (vocabulary); Flanker: Flanker Task (executive function); Pattern: Pattern Comparison Processing Speed Test (processing speed); Picture: Picture Sequence Memory Test (episodic memory); Reading: Oral Reading Recognition Task (reading ability). PC1: the first principal component of the above seven cognitive measures. Asterisks (*) indicate statistical significance after false discovery rate (FDR) correction at $q < 0.05$[32].

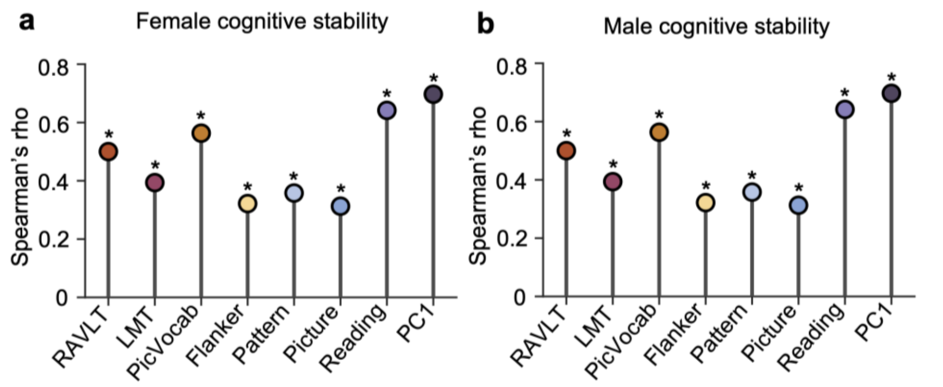

Figure S2. *Cognitive stability for* ***females*** *and* ***males***. (a) Spearman's correlation (stability) between baseline and Year 2 cognitive measures in females. The positive correlations indicate that children with higher baseline cognition generally maintained their cognitive advantage over their peers at Year 2. (b) Spearman's correlation (stability) between baseline and Year 2 cognitive measures in males. RAVLT: Rey Auditory Verbal Learning Test (verbal memory); LMT: Little Man Task (spatial reasoning); PicVocab: Picture Vocabulary Task (vocabulary); Flanker: Flanker Task (executive function); Pattern: Pattern Comparison Processing Speed Test (processing speed); Picture: Picture Sequence Memory Test (episodic memory); Reading: Oral Reading Recognition Task (reading ability). PC1: the first principal component of the above seven cognitive measures. Asterisks (*) indicate statistical significance after false discovery rate (FDR) correction at q < 0.05[1].

Table S1. Linear mixed-effects model results for females, males and sex differences in cognitive change.

	Female				Male				Sex differences			
	β	SE	t	p	β	SE	t	p	β	SE	t	p
RAVLT	-0.07119	0.01766	-4.03	**5.6×10^{-5}**	0.03784	0.01734	2.18	**0.03**	-0.10903	0.02475	-4.41	**1.1×10^{-5}**
LMT	0.00580	0.00023	24.96	**$< 1\times10^{-16}$**	0.00587	0.00023	25.72	**$< 1\times10^{-16}$**	-0.00007	0.00033	-0.22	0.82
PicVocab	0.16511	0.00854	19.34	**$< 1\times10^{-16}$**	0.18394	0.00839	21.92	**$< 1\times10^{-16}$**	-0.01882	0.01197	-1.57	0.12
Flanker	0.23165	0.01093	21.20	**$< 1\times10^{-16}$**	0.22971	0.01073	21.41	**$< 1\times10^{-16}$**	0.00194	0.01531	0.13	0.90
Pattern	0.63692	0.01927	33.04	**$< 1\times10^{-16}$**	0.61494	0.01894	32.47	**$< 1\times10^{-16}$**	0.02198	0.02702	0.81	0.42
Picture	0.23247	0.01661	14.00	**$< 1\times10^{-16}$**	0.26548	0.01631	16.28	**$< 1\times10^{-16}$**	-0.03301	0.02328	-1.42	0.16
Reading	0.15380	0.00663	23.19	**$< 1\times10^{-16}$**	0.15538	0.00652	23.85	**$< 1\times10^{-16}$**	-0.00158	0.00930	-0.17	0.86
PC1	0.05677	0.00135	41.99	**$< 1\times10^{-16}$**	0.06132	0.00133	46.18	**$< 1\times10^{-16}$**	-0.00455	0.00189	-2.40	**0.02**

Note that bold p-values denote effects that remained significant after false discovery rate (FDR) correction at q < 0.05.

(2) Sex differences in longitudinal FC changes. The spatial patterns of longitudinal FC stability and longitudinal FC changes were similar in males and females, and consistent with the full sample. Although females exhibited higher FC stability than male across the whole brain, differences were small and only 0.36% of edges survived FDR q < 0.05. In the case of longitudinal FC changes, females exhibited lower rate of FC decrease than males in certain brain regions, e.g., anterior insula. There was no statistically significant sex difference in the inter-individual variability in longitudinal FC change. We have updated the manuscript as follows:

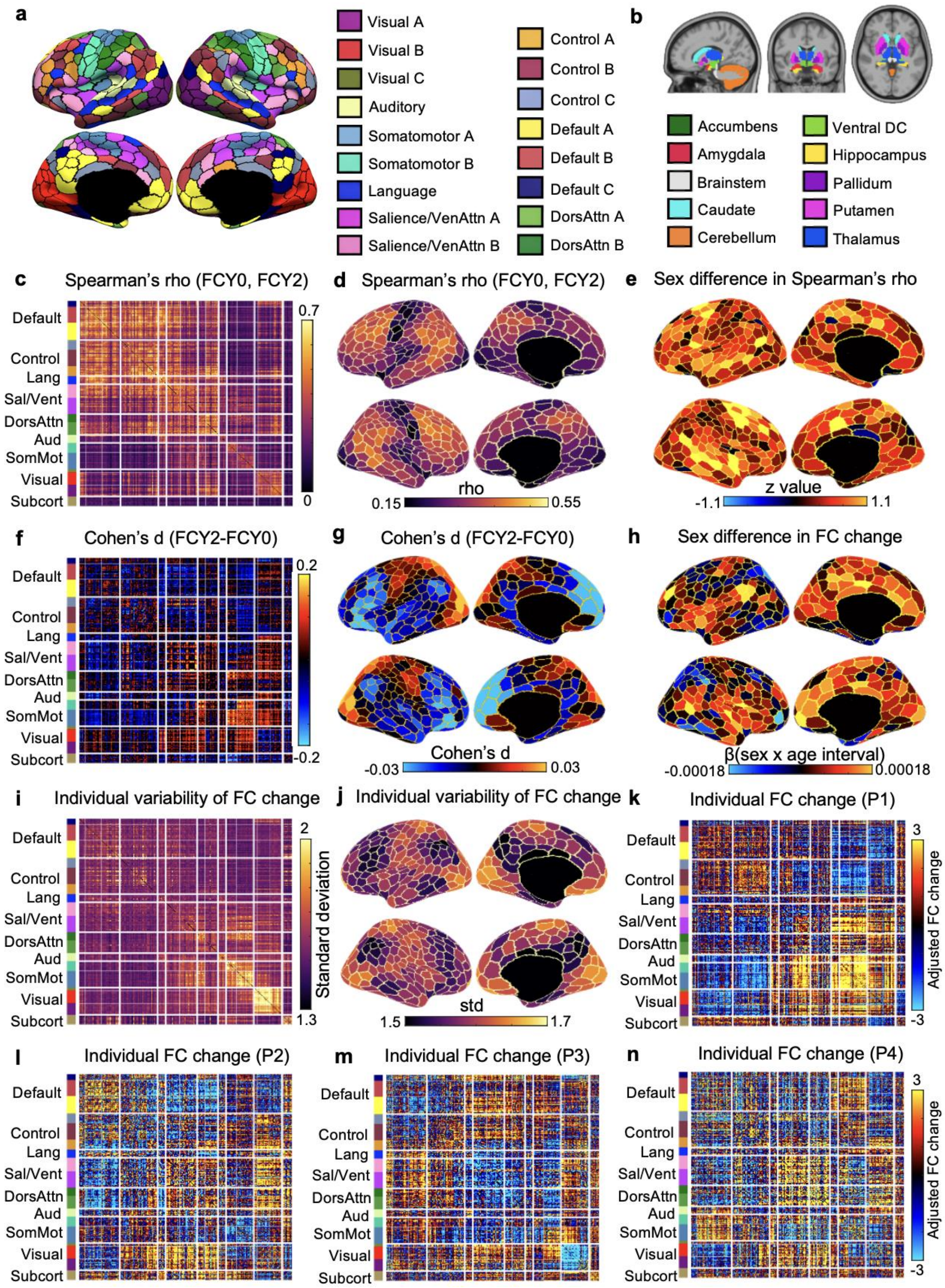
a
Visual A
Visual B
Visual C
Auditory
Somatomotor A
Somatomotor B
Language
Salience/VenAttn A
Salience/VenAttn B
Control A
Control B
Control C
Default A
Default B
Default C
DorsAttn A
DorsAttn B
b
Accumbens
Amygdala
Brainstem
Caudate
Cerebellum
Ventral DC
Hippocampus
Pallidum
Putamen
Thalamus
c Spearman's rho (FCY0, FCY2)
d Spearman's rho (FCY0, FCY2)
e Sex difference in Spearman's rho
rho
z value
f Cohen's d (FCY2-FCY0)
g Cohen's d (FCY2-FCY0)
h Sex difference in FC change
Cohen's d
β(sex x age interval)
i Individual variability of FC change
j Individual variability of FC change
k Individual FC change (P1)
Standard deviation
std
Adjusted FC change
l Individual FC change (P2)
m Individual FC change (P3)
n Individual FC change (P4)
Default
Control
Lang
Sal/Vent
DorsAttn
Aud
SomMot
Visual
Subcort

Figure 3. Individual differences in longitudinal FC change. (a) Cortical parcellation of 400 regions[33], which is the homotopic variant of the Schaefer parcellation[37]. Parcel colors are assigned corresponding to 17 large-scale networks[38]. (b) 19 subcortical regions[34]. 419 × 419 FC matrices were computed based on the 419 cortical and subcortical regions. (c) Spearman's correlation (stability) between baseline (FCY0) and Year 2 FC (FCY2) for each FC edge. Positive correlations indicate that children exhibiting stronger functional brain connectivity at baseline continued to exhibit stronger functional brain connectivity than their peers at Year 2. 99.995% of entries were significant after FDR correction with $q < 0.05$. (d) Visualization of FC stability at the regional level, by averaging the rows of panel c. (e) Sex differences in FC stability between baseline and Year 2, summarized at the regional level without thresholding. At the edge level, sex differences were sparse, with only 0.36% of connections reaching statistical significance in the linear mixed-effects model, after correcting for multiple comparisons with FDR $q < 0.05$ (Figure S3g). (f) Longitudinal FC change at the group level based on a linear mixed effects model. 54.96% of entries were significant after FDR correction with $q < 0.05$. (g) Visualization of longitudinal FC change at the regional level, by averaging the rows of panel f. (h) Sex differences in FC change rate between baseline and Year 2, summarized at the regional level without thresholding. At the edge level, only 4% connections reaching statistical significance in the linear mixed-effects model, after correcting for multiple comparisons with FDR $q < 0.05$ (Figure S3o). (i) Individual variability in longitudinal FC change. FC change (z value) was computed for each FC edge[39]. Sex, baseline age, age interval (between baseline and Year 2) and head motion at two timepoints were regressed out. Standard deviation was then computed across individuals. (j) Visualization of individual variability in longitudinal FC change at the regional level, by averaging the rows of panel i. (k-n) Individual level FC change for four participants.

page 7 (Results)

By averaging the rows of Figure 3c, clear regional variation was observed along the sensory-association (S-A) axis[35,36], with heteromodal association cortex being the most stable, while sensory-motor and visual systems were the least stable (Figure 3d). Similar patterns were observed separately for males and females (Figures S3a–S3d). Females exhibited higher FC stability than males across almost all brain regions (Figure 3e), but these differences were small and few regions survived FDR correction (Figures S3e–S3h).

Longitudinal FC change also mirrored the S-A axis: sensory-motor and visual systems showed the greatest longitudinal increase, while heteromodal association cortex exhibited the greatest longitudinal decrease (Figures 3f and 3g). Similar patterns were observed separately for males and females (Figures S3i–S3l), but females exhibited statistically lower rate of FC decrease than male in association cortex, such as the anterior insula and anterior cingulate cortex (Figure 3h; Figures S3m–S3p).

Beyond group-level changes, we also observed notable individual differences in FC change, especially for somatomotor and visual networks, as well as medial prefrontal

cortex (Figures 3i and 3j). A similar pattern was observed for males and females with no statistically significant sex difference (Figures S3q–S3t).

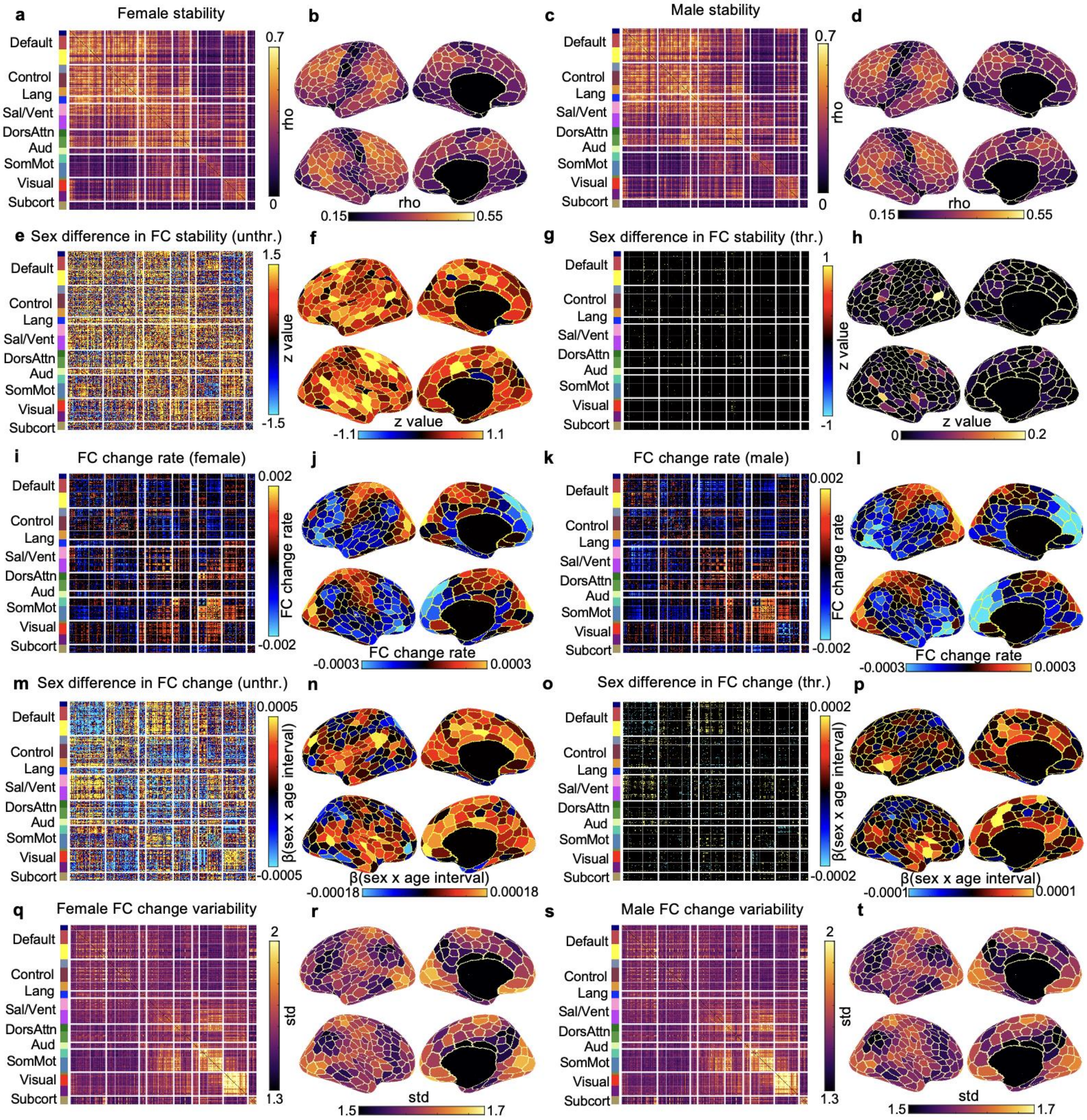

Figure S3. FC stability and longitudinal change in **females** and **males**. (a) Spearman's correlation (stability) between baseline (FCY0) and Year 2 FC (FCY2) for each FC edge in females. Positive correlations indicate that children exhibiting stronger brain connectivity at baseline continued to exhibit stronger brain connectivity than their peers at Year 2. (b) Regional visualization of female FC stability, by averaging the rows of panel a. (c) Spearman's correlation (stability) between baseline (FCY0) and Year 2 FC (FCY2) for each FC edge in males. (d) Regional visualization of male FC stability, by averaging the rows of panel c. (e) Unthresholded sex differences in FC stability (female – male). Positive implies greater stability in females. (f) Regional visualization of unthresholded sex differences in FC stability, by averaging the rows of panel e. (g) Thresholded sex differences in FC stability. (h) Regional visualization of thresholded

sex differences in FC stability, by averaging the rows of panel g. (i) Longitudinal FC change rate in females at the group level based on a linear mixed effects model. (j) Regional visualization of female longitudinal change rate, by averaging the rows of panel i. (k) Longitudinal FC change rate in males at the group level based on the linear mixed effects model. (l) Regional visualization of male longitudinal change, by averaging the rows of panel k. (m) Unthresholded sex differences in FC change (female – male). Positive indicates greater change in females. (n) Regional visualization of unthresholded sex differences in FC change, by averaging the rows of panel m. (o) Thresholded sex differences in FC change. (p) Regional visualization of thresholded sex differences in FC change, by averaging the rows of panel (o). (q) Female variability in longitudinal FC change. FC change (z value) was computed for each FC edge [2]. Baseline age, age interval (between baseline and Year 2) and head motion at two timepoints were regressed out. Standard deviation was then computed across individuals. (r) Regional visualization of female variability in longitudinal FC change, by averaging the rows of panel q. (s) Male variability in longitudinal FC change. (t) Regional visualization of male variability in longitudinal FC change, by averaging the rows of panel s. FC change rates correspond to the fixed effects of the age-interval term ($\mathrm{Age}_{it} - \mathrm{Age}_{i1}$) in the mixed-effects model (β_2 for males; $\beta_2 + \beta_4$ for females); see Equation 2 in Supplementary Methods S1.

(3) Sex differences in cross-sectional prediction. Both females and males showed strengthened FC–cognition relationships during development, consistent with the full sample combining males and females. In the case of cross-sectional prediction, there was no sex difference in prediction accuracies across all cognitive measures after multiple comparisons correction. We have updated the manuscript as follows:

page 10 (Results)

> In sex-stratified analyses, the three predictive models (FCY0→CogY0, FCY0→CogY2, FCY2→CogY2) were re-trained and re-evaluated separately for males and females. All cognitive measures were predicted better than chance, except that the Pattern Comparison Processing Speed Test was not predicted better than chance in males in the FCY0→CogY0 model (Figures S6 and S7). For both sexes, prediction accuracy also improved when predicting Year 2 cognition compared with baseline cognition. There was no sex difference in prediction accuracy for any model or cognitive measure after multiple comparisons correction with FDR $q < 0.05$.

page 12 (Results)

> In sex-stratified analyses, model transfer was also significantly better than chance for all cognitive measures for both females (Figure S10) and males (Figure S11). Similar to the full sample combining both males and females, model transfer prediction performance in Year 2 data was generally better than baseline data. There was no sex difference in model transfer prediction performance between males and females after multiple comparisons correction with FDR $q < 0.05$.

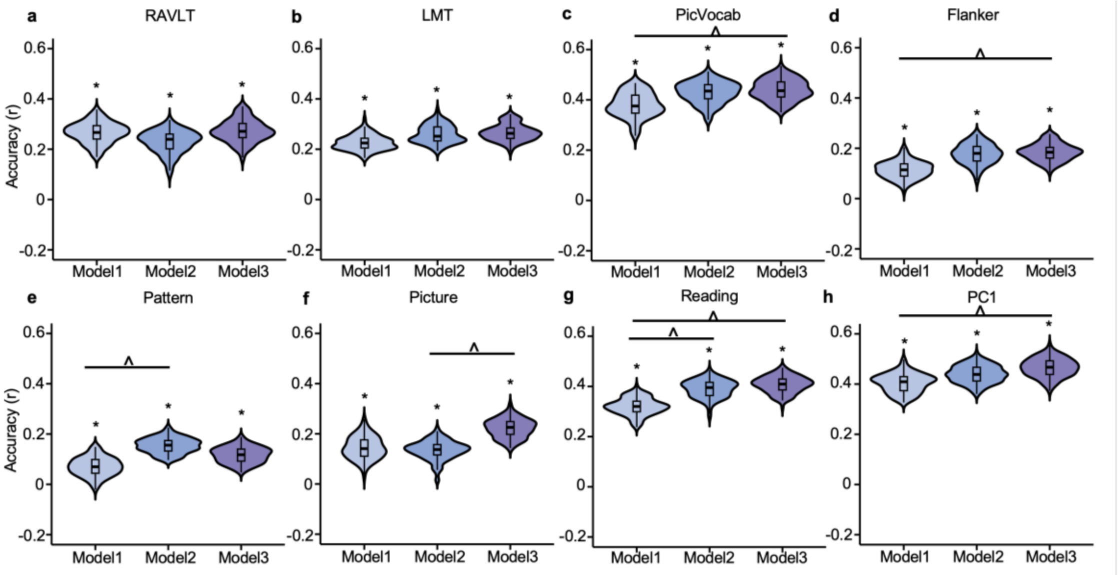

Figure S6. Enhanced FC-cognition relationship during development in **females**. (a-h) Comparison of prediction accuracies across the three models for the eight cognitive measures in females. Model 1 predicted baseline cognition using baseline FC (FCY0 → CogY0). Model 2 predicted Year 2 cognition using baseline FC (FCY0 → CogY2). Model 3 predicted Year 2 cognition using Year 2 FC (FCY2 → CogY2). Each value in the violin plot represents the accuracy (r) for a single cross-validation fold. Asterisks (*) denote above chance prediction after multiple comparisons correction (FDR $q < 0.05$). Carets (^) denote statistically significant differences between models based on the corrected resampled t-test (FDR $q < 0.05$). RAVLT: Rey Auditory Verbal Learning Test (verbal memory); LMT: Little Man Task (spatial reasoning); PicVocab: Picture Vocabulary Task (vocabulary); Flanker: Flanker Task (executive function); Pattern: Pattern Comparison Processing Speed Test (processing speed); Picture: Picture Sequence Memory Test (episodic memory); Reading: Oral Reading Recognition Task (reading ability). PC1: the first principal component of the above seven cognitive measures.

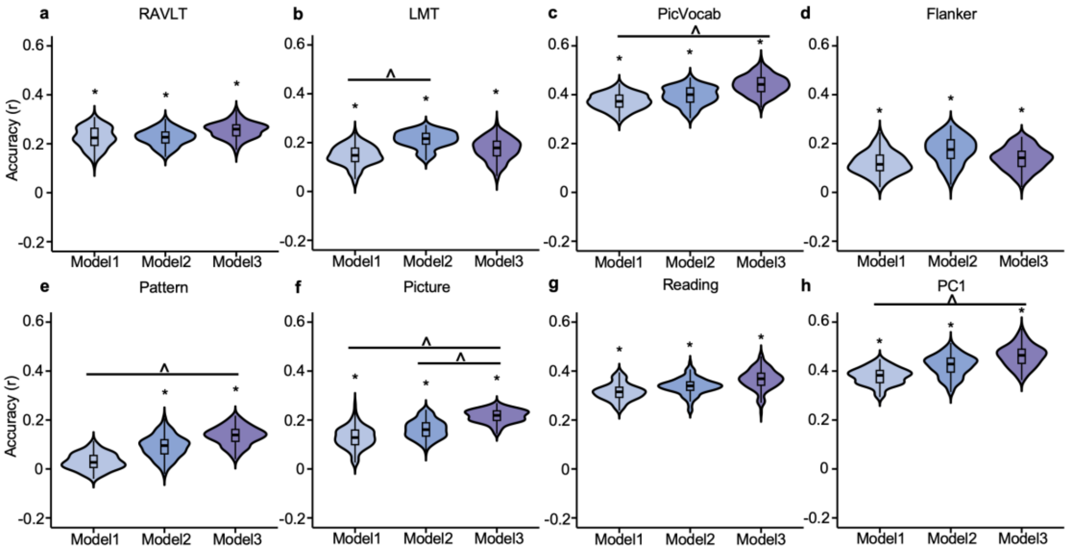

Figure S7. Same as Figure S6, but for **males**.

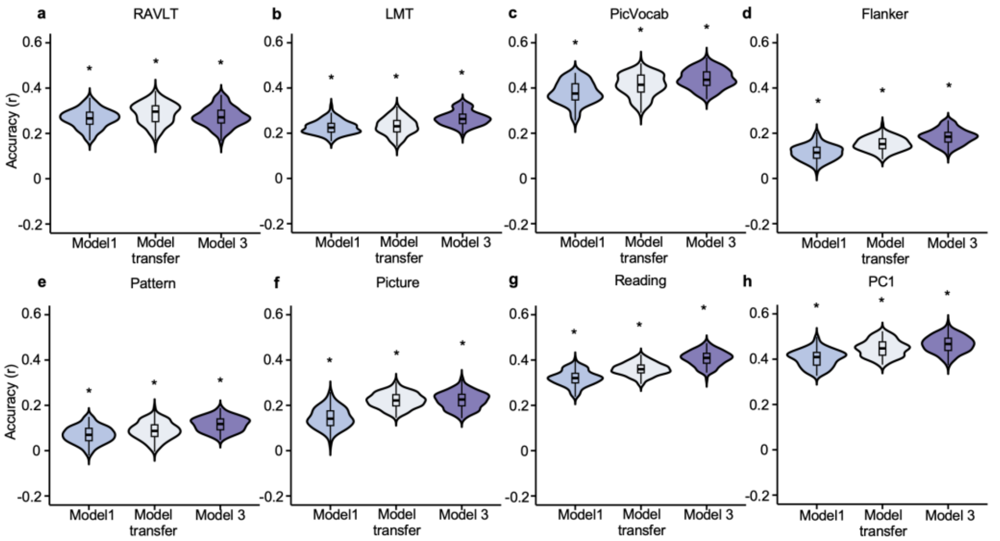

Figure S10. Models trained on baseline FC to predict baseline cognition improve in accuracy when applied to Year 2 FC and Year 2 cognition in **females**. Each plot corresponds to a different cognitive measure. “Model 1” are the models obtained by training baseline FC to predict baseline cognition (FCY0 → CogY0). These models were then used to predict Y2 cognition from Year 2 FC, which we refer to as “Model transfer”. Finally, for reference, “Model 3” are the models obtained by training Y2 FC to predict Y2 cognition (FCY2 → CogY2). Asterisks (*) denote above chance prediction after multiple comparisons correction (FDR $q < 0.05$). Carets (^) denote statistically significant differences between models based on the corrected resampled t-test (FDR $q < 0.05$). RAVLT: Rey Auditory Verbal Learning Test (verbal memory); LMT: Little Man Task (spatial reasoning); PicVocab: Picture Vocabulary Task (vocabulary); Flanker: Flanker Task (executive function); Pattern: Pattern Comparison Processing Speed Test (processing speed); Picture: Picture Sequence Memory Test (episodic memory); Reading: Oral Reading Recognition Task (reading ability). PC1: the first principal component of the above seven cognitive measures.

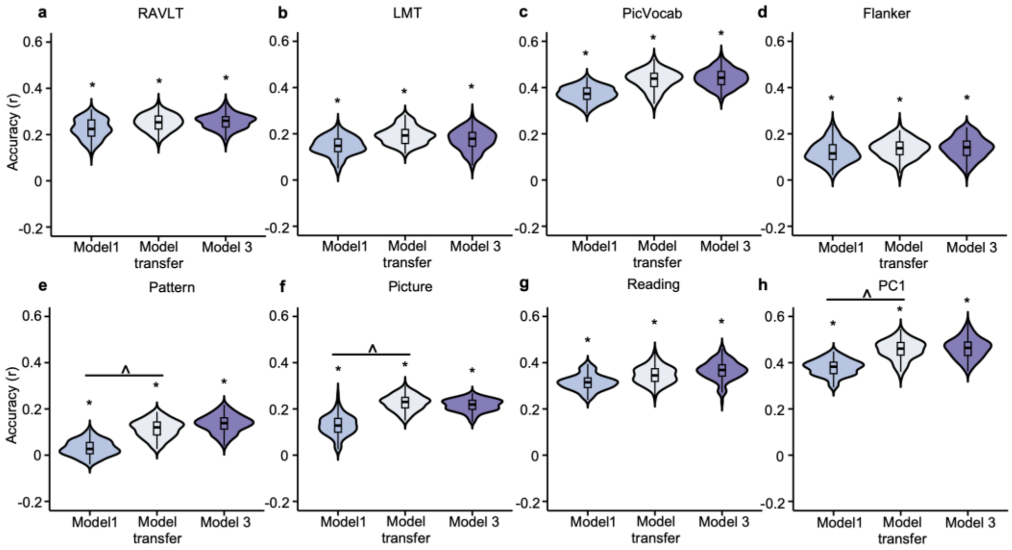

Figure S11. Same as S10, but for **males**.

(4) Sex differences in prediction of Year 2 cognition based on baseline FC or longitudinal FC change. For both males and females, baseline FC was better than longitudinal FC change in predicting Year 2 cognition. The result persisted even when we shorten scan duration of baseline fMRI to 4 mins. Across all predictive models, there was no sex difference in prediction performance. We have updated the manuscript as follows:

page 14 (Results)

> Consistent with the full sample, both baseline FC and 4-min baseline FC were better than longitudinal FC change in predicting Year 2 cognition for both females (Figure S14) and males (Figure S15). There was no sex difference in prediction performance for all predictive models after multiple comparisons correction with FDR $q < 0.05$.

(5) Sex differences in prediction of longitudinal cognition change. For both males and females, longitudinal change in cognition was poorly predicted by baseline FC, as well as longitudinal FC change. There was no sex difference for all predictive models. We have updated the manuscript as follows:

page15 (Results)

> Consistent with the full sample, longitudinal cognitive change was poorly predicted by baseline FC and longitudinal FC change for both females (Figure S17) and males (Figure S18). Due to the smaller sample size, no prediction of longitudinal cognitive change was better than chance. There was also no sex difference in prediction performance for all cognitive measures after multiple comparisons correction with FDR $q < 0.05$.

(6) Sex differences in convergent and divergent predictive network features (PNFs) between cross-sectional and longitudinal estimates of brain–cognition relationship. Consistent with the full sample, there were both convergent and divergent PNFs between cross-sectional and longitudinal models when models were trained separately for each sex. For example, connectivity within the salience network was convergent between cross-sectional and longitudinal models, not only for the full sample, but also separately for males and females. Similarly, connectivity within the visual network was divergent between cross-sectional and longitudinal models, not only for the full sample, but also separately for males and females.

On the other hand, there were also clear sex differences. Predictive network features were substantially more similar between males and females in the cross-sectional models than in the longitudinal models, giving rise to sex-specific convergences and divergences when compared with cross-sectional models. We have updated our manuscript as follows:

page 18 (Results)

> Consistent with results from the full sample, models trained separately for each sex showed both convergent and divergent predictive network features (PNFs) between cross-sectional and longitudinal analyses. For example, greater connectivity within the salience network predicted better baseline LMT performance in the cross-sectional models (Figures 8a and 8b), and greater longitudinal increases in salience network connectivity predicted greater improvements in LMT performance (Figures 8c and 8d), indicating feature-level convergence between cross-sectional and longitudinal models

(Figures 8e and 8f). In contrast, although greater visual network connectivity predicted better baseline LMT performance for both sexes in the cross-sectional models (Figures 8a and 8b), greater longitudinal decreases in visual network connectivity predicted better longitudinal improvement in LMT performance (Figures 8c and 8d), reflecting divergence between cross-sectional and longitudinal models (Figures 8g and 8h).

Predictive network features were substantially more similar between males and females in the cross-sectional models (r = 0.5; Figures 8a and 8b) than in the longitudinal models (r = −0.03; Figures 8c and 8d), indicating greater sex-specific divergence in longitudinal predictions. For example, in both females and males, weaker baseline FC within the somatomotor network predicted better baseline LMT performance in the cross-sectional models (Figures 8a and 8b). However, in females, greater longitudinal increases in somatomotor connectivity predicted greater improvement in LMT performance (Figure 8c), diverging from the cross-sectional pattern (Figure 8g). In contrast, in males, greater longitudinal decreases in somatomotor connectivity predicted greater improvement in LMT performance (Figure 8d), converging with the male-only cross-sectional model (Figure 8f).

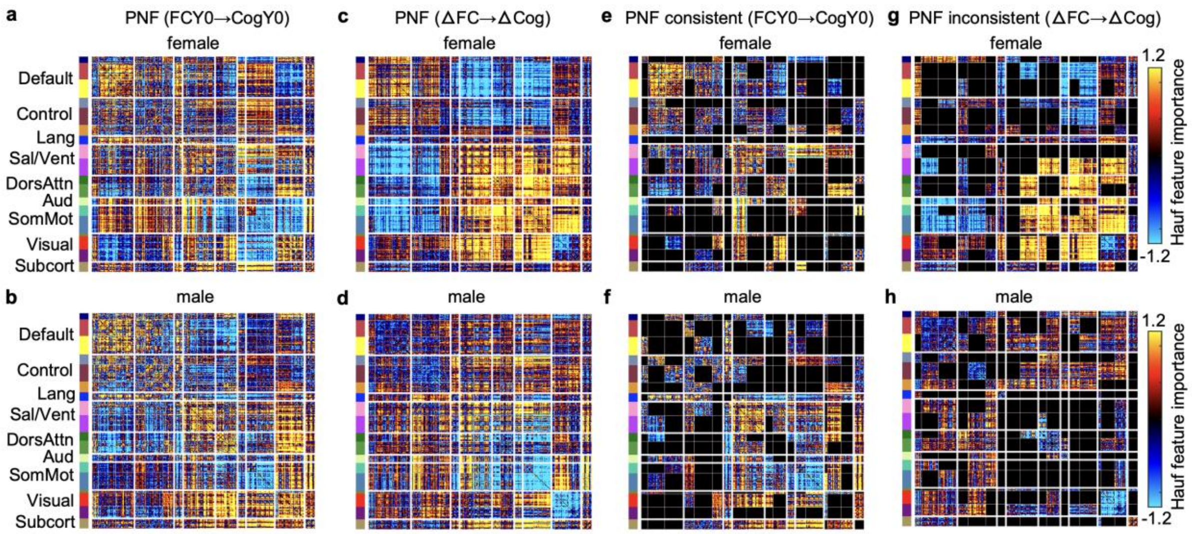

Figure 8. Convergent and divergent predictive network features (PNFs) between cross-sectional and longitudinal estimates of FC–cognition relationship within each sex. (a-b) PNFs from cross-sectional models using baseline FC (FCY0) to predict baseline Little Man Task (LMT) score (CogY0) in (a) females and (b) males separately. (c-d) PNFs from longitudinal models using changes in FC (ΔFC) to predict changes in LMT performance (ΔCog) across the two timepoints in (c) females and (d) males separately. (e-f) Network blocks with consistent PNFs between cross-sectional and longitudinal models in (e) females and (f) males. (g-h) Network blocks with inconsistent PNFs between cross-sectional and longitudinal models in (g) females and (h) males. PNFs were considered consistent if the average feature value for a network block had the same sign in both cross-sectional and longitudinal models. For visualization purposes, each predictive network feature matrix was normalized by dividing all values by the standard deviation of the entire matrix. Thresholded PNFs are shown in Figure S19.

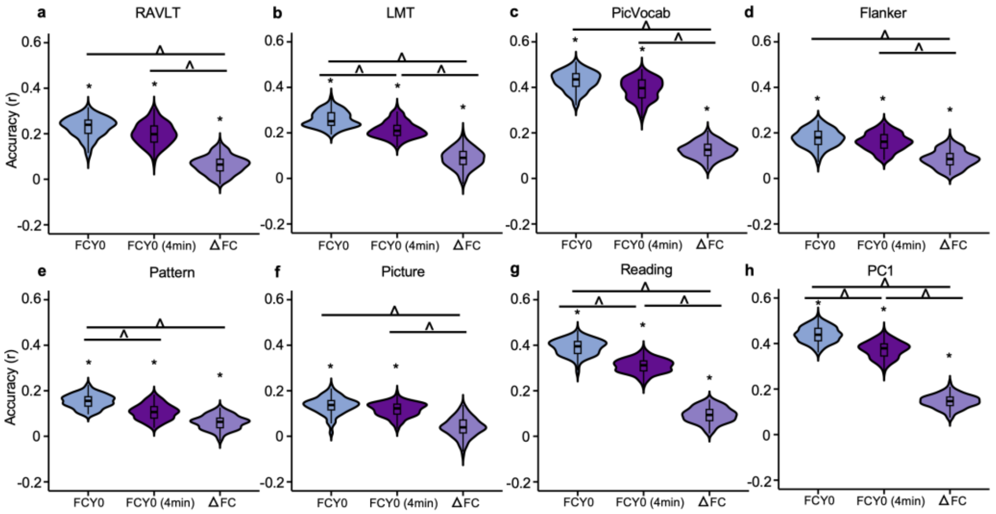

Figure S14. Baseline FC is more predictive of cognition at Year 2 than longitudinal FC change in **females**, even accounting for reliability differences. Each panel corresponds to the prediction accuracy of a different cognitive measure at Year 2. "FCY0" are the models obtained by training baseline FC to predict Year 2 cognition (same as model 2 in Figure 4). "FC (4min)" uses baseline FC computed from the first 4 minutes of fMRI data to predict Year 2 cognition. "ΔFC" are the models obtained by using FC change (between Year 2 and baseline) to predict cognition at Year 2. Asterisks (*) denote above chance prediction after multiple comparisons correction (FDR $q < 0.05$). Carets (^) denote statistically significant differences between models based on the corrected resampled t-test (FDR $q < 0.05$). RAVLT: Rey Auditory Verbal Learning Test (verbal memory); LMT: Little Man Task (spatial reasoning); PicVocab: Picture Vocabulary Task (vocabulary); Flanker: Flanker Task (executive function); Pattern: Pattern Comparison Processing Speed Test (processing speed); Picture: Picture Sequence Memory Test (episodic memory); Reading: Oral Reading Recognition Task (reading ability). PC1: the first principal component of the above seven cognitive measures.

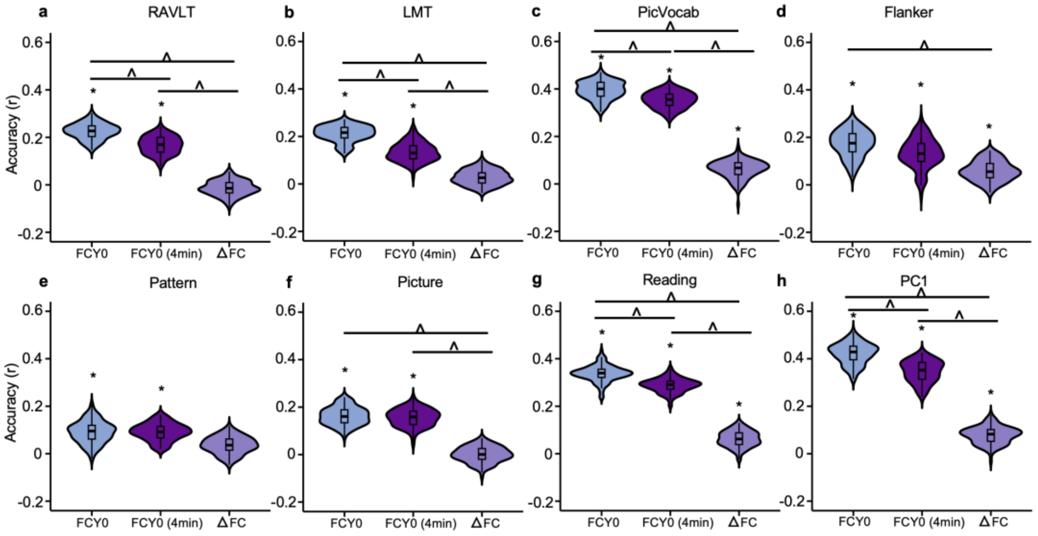

Figure S15. Same as Figure 14, but for **males**.

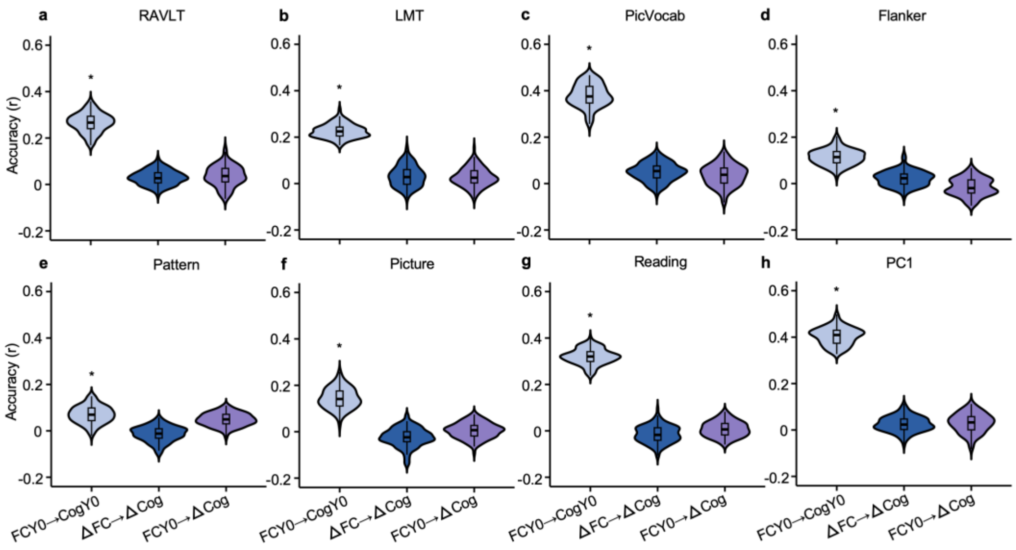

Figure S17. Limited prediction of cognitive change from baseline FC and longitudinal FC change in **females**. Each panel corresponds to the prediction accuracy of a different cognitive measure. Within each panel, baseline FC and longitudinal FC change (ΔFC) were used to predict cognitive change (ΔCog). Predictions of baseline cognition from baseline FC (FCY0 → CogY0) were also shown for reference. Asterisks (*) denote above chance prediction after multiple comparisons correction (FDR q < 0.05). RAVLT: Rey Auditory Verbal Learning Test (verbal memory); LMT: Little Man Task (spatial reasoning); PicVocab: Picture Vocabulary Task (vocabulary); Flanker: Flanker Task (executive function); Pattern: Pattern Comparison Processing Speed Test (processing speed); Picture: Picture Sequence Memory Test (episodic memory); Reading: Oral Reading Recognition Task (reading ability). PC1: the first principal component of the above seven cognitive measures.

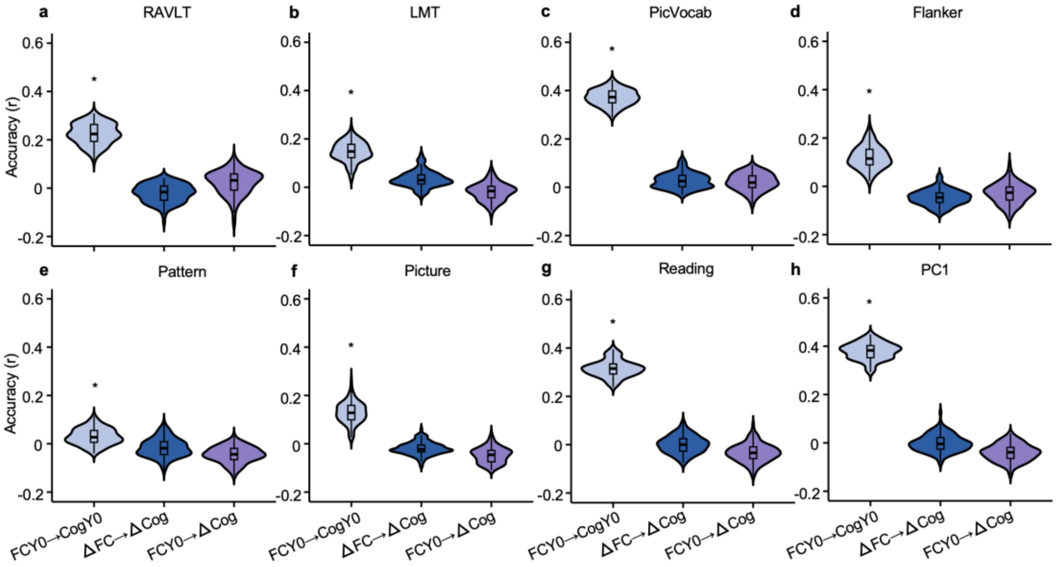

Figure S18. Same as Figure 17, but in **males**.

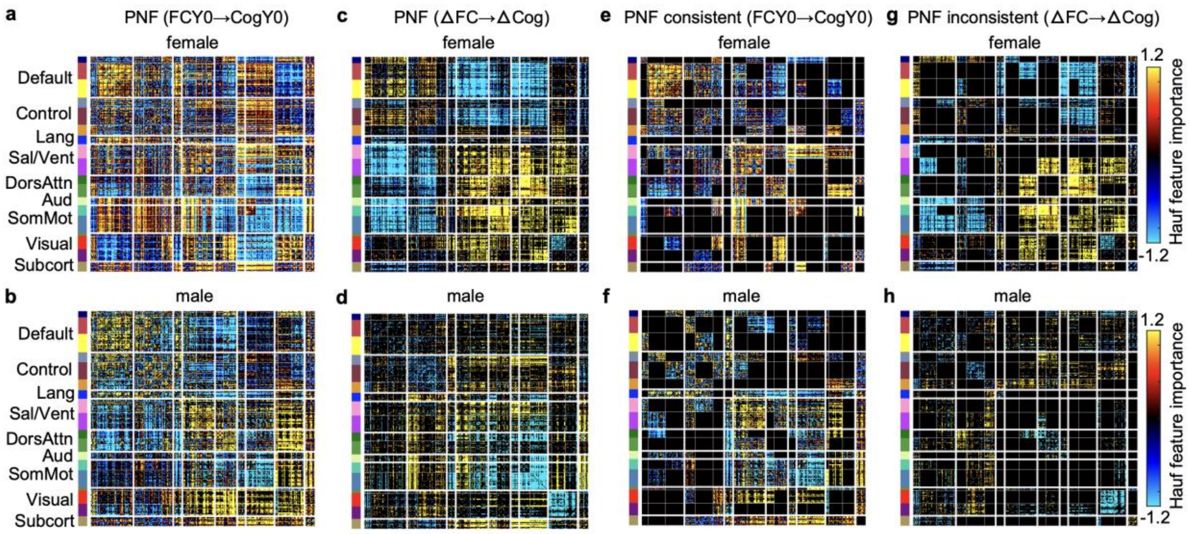

Figure S19. Convergent and divergent predictive network features (PNFs) between cross-sectional and longitudinal estimates of FC–cognition relationship within each sex. This figure is the same as Figure 8, except that only PNF values that survived FDR $q < 0.05$ are shown. (a-b) PNFs from cross-sectional models using baseline FC (FCY0) to predict baseline Little Man Task (LMT) score (CogY0) in (a) females and (b) males separately. Values were thresholded with FDR $q < 0.05$. (c-d) PNFs from longitudinal models using changes in FC (ΔFC) to predict changes in LMT performance (ΔCog) across the two timepoints in (c) females and (d) males separately. Values were thresholded with FDR $q < 0.05$. (e-f) Network blocks with consistent PNFs between cross-sectional and longitudinal models in (e) females and (f) males. (g-h) Network blocks with inconsistent PNFs between cross-sectional and longitudinal models in (g) females and (h) males. PNFs were considered consistent if the average feature value for a network block had the same sign in both cross-sectional and longitudinal models. For visualization purposes, each predictive network feature matrix was normalized by dividing all values by the standard deviation of the entire matrix.

page 22-23 (Discussion)

Sex differences in cognition, FC, and FC-cognition relationships

Previous studies have documented sex differences in cognition, brain function, and their interrelationships[75-80]. However, much of this literature relies on cross-sectional designs spanning broad and heterogeneous age ranges, limiting inferences into sex-specific developmental trajectories. Our study systematically examined longitudinal developmental patterns in cognition, FC, and FC–cognition relationships across sexes during the transition from childhood to early adolescence.

With respect to cognitive development, we did not observe a significant overall longitudinal improvement in RAVLT performance, consistent with a previous ABCD study[81]. Notably, however, we detected a significant sex-by-age interval interaction, with males showing a positive slope and females showing a negative slope over time (Figure 2). These opposing trajectories likely attenuated sensitivity to longitudinal effects when sexes were analyzed together. In addition, sex differences in the variance of individual-

level longitudinal cognitive change were observed for RAVLT and the Pattern Comparison Processing Speed Test, indicating measure-specific heterogeneity rather than pervasive sex differences. In the case of longitudinal FC change, females generally exhibited a slower rate of FC decrease than males, especially in association cortex (Figure 3).

Turning to brain–behavior relationships, prior cross-sectional analyses of ABCD data have reported some sex differences[79,82]. Consistent with these findings, we observed sex-specific patterns in cross-sectional predictive models (Figures 8a and 8b), alongside substantial overlap in predictive network features, paralleling results from cross-sectional studies in young adults[83]. In contrast, predictive network features diverged more strongly between males and females in the longitudinal models (Figures 8c and 8d), giving rise to sex-specific convergences and divergences when compared with cross-sectional models (Figures 8e to 8h).

pages 4-6 (Supplementary Methods)

S1. Sex-stratified and sex difference analyses

Longitudinal cognitive stability. Based on the harmonized cognitive scores obtained after longitudinal ComBat (as described in the main Methods), analyses were conducted separately in females and males. For each sex, baseline age was regressed from the harmonized baseline cognitive scores, and Year 2 age was regressed from the harmonized Year 2 cognitive scores. Sex was not included as a covariate in these models, as analyses were performed within sex. Longitudinal cognitive stability was then assessed by computing Spearman's correlations between the resulting residuals.

To compare sex difference in cognitive stability, we compared the correlation coefficients between baseline and Year 2 cognitive scores across females and males for each cognitive measure. Specifically, correlation coefficients were then transformed to Fisher's z values using the Fisher r-to-z transformation. The difference between the two Fisher-transformed correlations was evaluated using a z test, with the standard error calculated as $\sqrt{\frac{1}{n_{female}-3}+\frac{1}{n_{male}-3}}$, where n_{female} and n_{male} denote the sample sizes for females and males, respectively. Two tailed p values were derived from the standard normal distribution. Multiple comparisons were controlled using the false discovery rate (FDR) with $q < 0.05$ [1].

Longitudinal cognitive change. To examine longitudinal change in cognitive performance and potential sex differences, we fitted linear mixed-effects models including an interaction between sex and age interval. Analyses were conducted on cognitive scores harmonized using longitudinal ComBat. Specifically, baseline age (Age_{i1}), age interval between baseline and Year 2 ($\text{Age}_{it} - \text{Age}_{i1}$), sex, and the sex × age-interval interaction were included as fixed effects. In addition, subject-specific random intercepts and random slopes for age interval were modeled to capture individual differences in baseline cognition and rates of longitudinal change:

$$\text{Cognition}_{it} = \beta_0 + \beta_1\text{Age}_{i1} + \beta_2 (\text{Age}_{it} - \text{Age}_{i1}) + \beta_3 \text{Sex}_i + \beta_4(\text{Age}_{it} - \text{Age}_{i1}) \times \text{Sex}_i + b_i + c_i (\text{Age}_{it} - \text{Age}_{i1}) + \varepsilon_{it}, \quad (1)$$

where *i* indexes the *i*-th participant, *t* references the time point, Cognition_{it} is the cognition of the *i*-th participant at timepoint t after longitudinal ComBat. The term b_i and c_i represent participant-specific random intercepts and random slope, respectively. However, we note that Equation 1 can only capture a group-level estimate of longitudinal change. Therefore, to capture the potential individual differences in longitudinal cognition change, for each participant and each cognitive score, we computed the longitudinal cognition change as: Cognition_{i2} - Cognition_{i1}, where Cognition_{it} is the cognition of the *i*-th participant at timepoint t (from longitudinal ComBat). We then regressed out baseline age and age interval (i.e., between Year 2 and baseline) from the individual-level longitudinal cognition change estimate.

We also examined sex differences in the variability of longitudinal cognitive change. Specifically, residualized longitudinal cognitive change values from females and males were pooled and grouped by sex, and the Brown–Forsythe test was applied to assess differences in variance between the two groups. In addition, the standard deviation (SD) of longitudinal cognitive change was computed separately for females and males for each cognitive measure, and the ratio of SDs (female/male) was calculated to summarize the magnitude and direction of sex differences in variability, with males used as the reference group. Two-tailed p values from the Brown–Forsythe tests were retained for each cognitive measure.

Longitudinal FC stability. Based on functional connectivity (FC) measures harmonized using longitudinal ComBat (as described in the main Methods), analyses were performed separately in females and males. Within each sex, baseline age and mean framewise displacement (FD) were regressed from the harmonized baseline FC, and Year-2 age and mean FD were regressed from the harmonized Year-2 FC. Sex was not included as a covariate in these models, as all analyses were conducted within sex. Longitudinal FC stability was then quantified by computing Spearman's correlations between the resulting residuals.

Sex differences in FC stability were assessed following the same procedure used for cognitive stability, by comparing Fisher z–transformed Spearman correlation coefficients between females and males.

Longitudinal FC change. To examine longitudinal change in FC and potential sex differences, we fitted linear mixed-effects models including an interaction between sex and age interval. Analyses were conducted on FC harmonized using longitudinal ComBat. Specifically, baseline age (Age_{i1}), age interval between baseline and Year 2 ($\text{Age}_{it} - \text{Age}_{i1}$), sex, sex × age-interval interaction and mean FD were included as fixed effects. In addition, subject-specific random intercepts and random slopes for age interval were modeled to capture individual differences in baseline cognition and rates of longitudinal change:

$$\mathrm{FC}_{it} = \beta_0 + \beta_1 \mathrm{Age}_{i1} + \beta_2 (\mathrm{Age}_{it} - \mathrm{Age}_{i1}) + \beta_3 \mathrm{Sex}_i + \beta_4 (\mathrm{Age}_{it} - \mathrm{Age}_{i1}) \times \mathrm{Sex}_i + \beta_5 \mathrm{meanFD}_{it} + \mathrm{b}_i + \mathrm{c}_i (\mathrm{Age}_{it} - \mathrm{Age}_{i1}) + \varepsilon_{it}, \quad (2)$$

where i indexes the i-th participant, t references the time point. FC_{it} is the FC value of a particular FC edge of the i-th participant at timepoint t after longitudinal ComBat. The term b_i and c_i represent participant-specific random intercepts and random slope, respectively.

However, we note that Equation 2 captures only the group-level estimate of longitudinal change. Therefore, to capture the potential individual differences in longitudinal FC change, for each participant and each FC edge, we computed the z value of FC change across the two timepoints:

$$z = \frac{r_2 - r_0}{\sqrt{s_2 + s_0}}, \quad (3)$$

where r_2 and r_0 denote Fisher r-to-z transformed FC value for the FC edge at Year 2 and baseline respectively. s_2 and s_0 denote the variance of the FC value for each FC edge at Year 2 and baseline respectively. The estimated variance needed to account for auto-correlation in the fMRI time series, so we used the MATLAB function xDF.m (https://github.com/asoroosh/xDF) to estimate r and s for each FC edge at each time point [2]. We then regressed, baseline age and age interval (between Year 2 and baseline), mean head motion (FD) at baseline, mean head motion (FD) at Year 2 from the z-statistic across participants.

Sex differences in the variability of longitudinal FC change were assessed following the same procedure used for longitudinal cognitive change.

Prediction analysis. All prediction analyses were repeated separately in females and males following the same procedure as the pooled analysis, with sex omitted as a covariate. We also compared sex differences in prediction accuracy for each model using corrected resampled t tests. Specifically, for each model, prediction accuracies were obtained across 120 cross-validation folds separately for females and males, and sex differences in accuracy were evaluated by comparing fold-wise accuracies across the two groups while accounting for dependencies across folds.

Minor clarifications

R2Q4. Line 247 – what fold for nested cross-validation? It would be good to outline this here – I realize it's 3 sites left out etc but this could be somewhat clarified here.

Response: Thanks for the suggestion! We have updated the text to include "leave-3-site-cluster-out":

page 9 (Results)

This was achieved via a leave-3-site-cluster-out nested cross-validation procedure, repeated 120 times for robustness (see Methods for details).

R2Q5. Line 253 - Just predicting PC1. Is there not more to learn with respect to the predictive powers for the 7 scores? This seems to be under treated and a lot more could be done with the data. The predictive powers shown in the supplementary section are mostly quite high.

Response: We thank the reviewer for raising this point. To clarify, all analyses were conducted for all cognitive measures. However, to streamline the presentation of results, we focus on PC1 when the overall trend was consistent across cognitive measures. PC1 was chosen as the representative cognitive measure because PC1 is conceptually a summary of general cognitive performance and yielded the highest prediction accuracy in most scenarios. Focusing on PC1 thus facilitates a clearer presentation of the overall FC–cognition relationships. When results for the seven individual cognitive measures are not shown in the main text, they are provided as Supplementary Results for completeness.

We have now made this rationale explicit in the manuscript. With regards to the particular section mentioned by the reviewer, we have also added a paragraph highlighting individual cognitive measures with high prediction accuracy like PC1.

page 9-10 (Results)

> The cross-validation procedure was repeated for all eight cognitive measures. To streamline the result representation, we will focus on the first cognitive principal component (PC1) when a similar trend was observed across all eight cognitive measures. PC1 was chosen as the representative measure because PC1 provides a parsimonious summary of general cognitive performance and often yielded the highest prediction accuracy. Results for the seven individual cognitive measures are reported in the supplement when not shown in the main text.
>
> …
>
> Figure S4 shows the comparisons for the other seven cognitive measures. Picture Vocabulary Task and Oral Reading Recognition Task exhibited comparatively high prediction accuracy, consistent with their strong contributions to the general cognitive ability component captured by PC1. All seven cognitive measures exhibited the same trend as PC1, in which prediction of Year 2 cognition was better than prediction of baseline cognition.

R2Q6. Line 262 – Here it is stated that at the later age there may be enhanced relationships between brain and behavior but this is why we need the motion data – it could also just be better MRI data because there is less motion as the kids get older and gain experience. What's the motion difference by release?

Response: We thank the reviewer for raising this very important point. As detailed in our response to R2Q2, we report the raw mean and standard deviation of framewise displacement (mean FD and std FD) in Table 1. Results from linear mixed-effects models examining the relationship between motion and age are reported in the text. In addition, relationships between

motion and cognition in Table S2. As suspected by the reviewer, as children grow older, they move less. Children with greater cognitive ability also move less.

We note that in our original manuscript, we did perform a control analysis in which we repeated the analyses using a subset of participants with similar motion across the two timepoints, and found similar conclusions, i.e., prediction of Year 2 cognition was better than prediction of baseline cognition. For your convenience, we have included the original results below.

page 12 (Results)

> To ensure that our results were not due to better quality fMRI data at Year 2, we repeated the "model transfer" analysis in this section using a subset of participants with comparable motion levels across the two time points (p = 0.18), yielding similar conclusions (Figure S9).

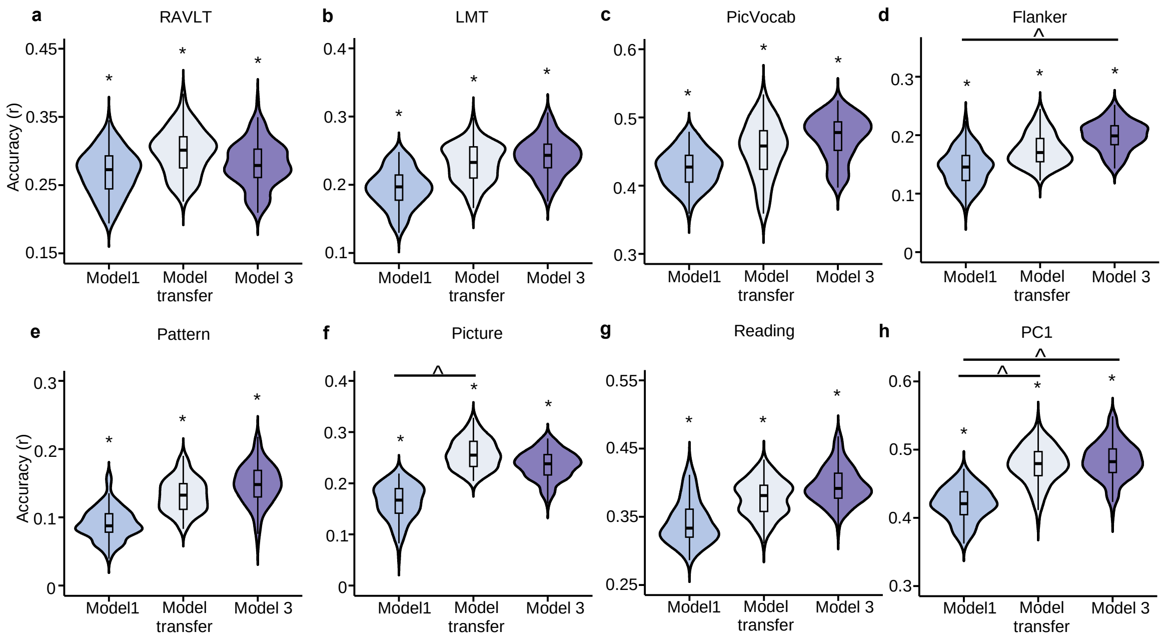

Figure S9. Models trained on baseline FC to predict baseline cognition improve in accuracy when applied to Year 2 FC and Year 2 cognition, even after controlling for head motion. Head motion was controlled by selecting a subset of participants (n = 2642) with no significant difference in mean frame-wise displacement across the two timepoints (p = 0.18). Each plot corresponds to a different cognitive measure. "Model 1" are the models obtained by training baseline FC to predict baseline cognition (FCY0 → CogY0). These models were then used to predict Year 2 cognition from Year 2 FC, which we refer to as "Model transfer". Finally, for reference, "Model 3" are the models obtained by training Y2 FC to predict Year 2 cognition (FCY2 → CogY2). Asterisks (*) denote above chance prediction after multiple comparisons correction (FDR q < 0.05). Carets (^) denote statistically significant differences between models based on the corrected resampled t-test (FDR q < 0.05). Note: Y-axes differ across panels to enhance visibility and emphasize the performance of models within each task. Comparisons across tasks should be made with caution due to varying scales. RAVLT: Rey Auditory Verbal Learning Test (verbal memory); LMT: Little Man Task (spatial reasoning); PicVocab: Picture

Vocabulary Task (vocabulary); Flanker: Flanker Task (executive function); Pattern: Pattern Comparison Processing Speed Test (processing speed); Picture: Picture Sequence Memory Test (episodic memory); Reading: Oral Reading Recognition Task (reading ability). PC1: the first principal component of the above seven cognitive measures.

In addition, as suggested in R1Q8, we also performed a control analysis using Year 2 FC to predict baseline cognition (FCY2→CogY0). If the higher prediction accuracy at Year 2 were primarily driven by lower motion or improved fMRI quality, then FCY2 should outperform FCY0 in predicting CogY0. However, FCY2→CogY0 did not surpass FCY0→CogY0 (Table S3), indicating that higher-quality Year 2 FC estimates did not inherently yield higher predictive performance compared to baseline FC.

Taken together, these control analyses demonstrate that the enhanced FC–cognition associations observed at Year 2 cannot be attributed to differences in head motion or MRI data quality.

page 12 (Results)

> In addition, we also used Year 2 FC to predict baseline cognitive ability (FCY2→CogY0). If the higher prediction accuracy at Year 2 were primarily driven by lower motion or improved fMRI quality, then FCY2 should outperform FCY0 in predicting CogY0. However, prediction accuracies of FCY2→CogY0 were similar to the baseline model (FCY0→CogY0), but lower than prediction of Year 2 cognition (FCY2→CogY2) across all cognitive measures (Table S3).

Table S3. Prediction accuracy for each cognitive measure and each cross-sectional model

	FCY0-CogY0	FCY0-CogY2	FCY2-CogY2	ModelTransfer	FCY2-CogY0
RAVLT	0.28	0.27	0.30	0.31	0.28
LMT	0.20	0.25	0.26	0.25	0.22
PicVocab	0.42	0.45	0.47	0.46	0.42
Flanker	0.14	0.20	0.19	0.17	0.12
Pattern	0.08	0.12	0.15	0.12	0.10
Picture	0.19	0.19	0.24	0.25	0.18
Reading	0.35	0.39	0.41	0.39	0.36
PC1	0.43	0.46	0.49	0.48	0.44

Reviewer #3:

R3Q1. In the current work, Xie et al investigated concurrent and predictive relationships between 10-12-year-olds brain functional connectivity and cognitive outcomes. 2949 subjects from the ABCD project were used in this investigation. The results revealed that baseline FC correlates with baseline cognition better than baseline cognition does. Baseline FC predicted Yr2 cognition but not as good as Yr2 FC predicted Yr2 cognition. When applying the baseline FC-cognition prediction model to Yr2 FC, it predicted Yr2 cognition better than baseline cognition. Finally, Baseline FC is better at predicting Yr2 cognition than longitudinal changes do, even after accounting for reliability differences. Strengths of this work include large sample size from the well-curated ABCD data, solid methodological selection and implementation, and careful comparisons of different prediction models. I do have some concerns on the generalizability of the very specific findings and claims.

Response: We thank the reviewer for the positive comments and valuable suggestions.

R3Q2. It is well known that missing data in ABCD is not necessarily random and the odds of a participant's missing data is related to demographic, behavioral, and/or clinical variables. In this project, only 2949 subjects were selected from >10000 datasets with strict inclusion criteria of having good quality MRIs at two consecutive time points as well as complete cognitive measures, the potential bias associated with this exclusion practice can not be overlooked and whether the conclusion is generalizable is questionable.

Response: The reviewer has raised an important point. Indeed, the excluded participants tend to be male, non-White children and those from lower socioeconomic backgrounds. To ensure our results are not due to a non-representative sample, we have constructed a subset of participants (N = 2,020) that matched the full ABCD baseline cohort in terms of age, sex, household income, race/ethnicity, and baseline cognition. This subset of participants did not statistically differ from the excluded participants on any of the matching variables after multiple comparisons correction. We then repeated all primary analyses in this subsample, and we found that very similar conclusions as the original main analysis. We have updated out manuscript as follows.

page 19 (Results):

> **Replication in a more representative subsample**
> Filtering participants based on missing data and head motion can reduce the representativeness of the data sample[11,47-50]. To ensure our results are not due to a non-representative sample, we constructed a matched subsample from the sample used in the main analyses by jointly matching age, sex, household income, race/ethnicity, and baseline cognition (PC1) to the full ABCD baseline cohort. This procedure yielded a matched subsample of 2,020 participants that approximated the demographic and cognitive distributions of the full ABCD baseline cohort. Included and excluded participants did not differ significantly on any matching variables after multiple comparisons correction with FDR $q < 0.05$ (Table S4).
>
> Across all primary analyses, results obtained from the representativeness-matched subsample closely mirrored those from the full analytic cohort (Figures S21 to S27).

More specifically, FC stability and longitudinal change mirrored the S-A axis (Figure S22). Furthermore, the relationship between FC and cognition strengthened with development (Figures S23 and S24). Baseline FC was more predictive of cognition at Year 2 than longitudinal FC change (Figure S25). Both baseline FC and longitudinal FC change weakly predicted cognitive change (Figure S26). Finally, we observed convergent and divergent predictive network features between cross-sectional and longitudinal models (Figure S27).

page 30-31 (Methods)

Replication in a more representative subsample

To ensure our results are not due to a non-representative sample, we generated a subset of participants (N = 2,020) from the main analytic cohort (N = 2,949) such that its joint distribution of key demographic variables (age, sex, household income, and race/ethnicity) and baseline cognition approximated that of the full ABCD baseline cohort. All matching variables were discretized into predefined bins, with two bins for age and sex, five bins for race/ethnicity, four bins for household income, and three bins for baseline cognition (PC1). Participants in the analytic cohort were assigned to multi-dimensional bins defined by the cross-classification of these variables, and target counts for each bin were set to approximate their proportions in the full ABCD baseline cohort, subject to availability constraints in the analytic sample. A fixed-size subsample was then selected to match these target bin counts.

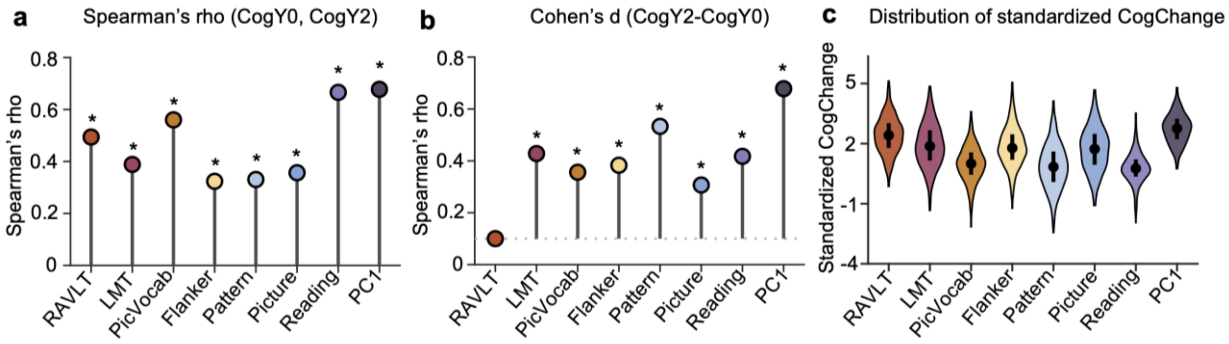

Figure S21. Individual differences in longitudinal cognitive change during the transition from childhood to adolescence in a representative subsample (N = 2020). (a) Spearman's correlation between baseline and Year 2 cognitive measures. The positive correlations indicate that children with higher baseline cognition generally maintained their cognitive advantage over their peers at Year 2. (b) Longitudinal cognitive change at the group level estimated from a linear mixed effects model. (c) Individual variability in standardized longitudinal cognitive change. For each individual and each cognitive measure, cognition change was defined as the difference in scores between the two timepoints. Sex, baseline age and age interval (between baseline and Year 2) were regressed out. The residualized change scores were then standardized by the baseline standard deviation of each measure. Violin plots show the distribution of standardized cognitive change, with dots indicating the median and bars indicating the interquartile range (25th–75th percentile)). Distributions are shown for descriptive purposes only.

Table S4. Compare distribution of included and excluded participants after matching

Variable	Included (Matched)	Excluded	p
Sample size (N)	2020	9848	N.A.
Age (month)	118.89 (7.40)	119 (7.52)	0.53
Female (%)	48.37	47.74	0.64
race (%)	A/B/H/W/O	A/B/H/W/O	0.03
Asian	54.21	51.57	—
Black	13.07	15.44	—
Hispanic	20.74	20.22	—
White	1.68	2.21	—
Other	10.30	10.55	—
Income (%)	L1/L2/L3/U	L1/L2/L3/U	0.51
L1: < 50K	27.13	27.15	—
L2: >= 50K & < 100K	26.14	25.79	—
L3: >=100K	39.01	38.31	—
Unknown	7.72	8.74	—
RAVLT	68.66 (15.36)	67.71(15.53)	0.03
LMT	0.60 (0.17)	0.59 (0.17)	0.01
PicVocab	84.70 (7.97)	84.40 (8.09)	0.20
Flanker	94.40 (8.62)	93.92 (9.18)	0.08
Pattern	88.13 (13.91)	88.04 (14.60)	0.73
Picture	103.20 (12.06)	102.73 (11.98)	0.17
Reading	90.98 (6.86)	90.83 (6.87)	0.26
PC1	0.09 (1.61)	-0.02 (1.66)	0.04

Values are means ± SD for continuous variables and percentages for categorical variables. For categorical variables with multiple levels (sex, race and household income), p-values reflect χ^2 tests comparing overall distributions between included and excluded participants, and sub-category rows are descriptive only. For continuous variables, p-values were obtained using Wilcoxon rank-sum tests. Baseline cognitive matching was performed using PC1; individual cognitive measures are shown for descriptive assessment of balance. There is no significant difference after multiple comparisons correction with FDR q <0.05.

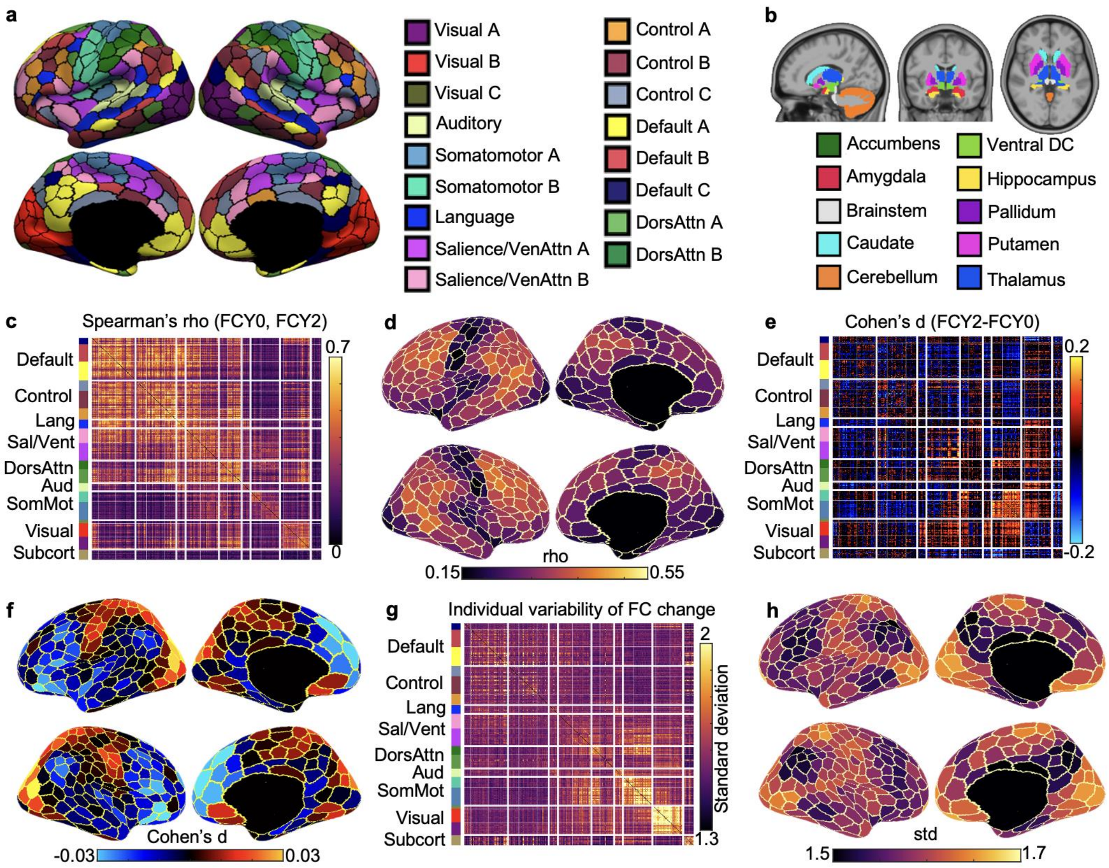

Figure S22. Individual differences in longitudinal functional connectivity (FC) change in a representative subsample (N = 2020). Individual differences in longitudinal functional connectivity (FC) change. (a) Cortical parcellation of 400 regions [4], which is the homotopic variant of the Schaefer parcellation [5]. Parcel colors are assigned corresponding to 17 large-scale networks [6]. (b) 19 subcortical regions [7]. 419 × 419 FC matrices were computed based on the 419 cortical and subcortical regions. (c) Spearman's correlation (stability) between baseline (FCY0) and Year 2 FC (FCY2) for each FC edge. Positive correlations indicate that children exhibiting stronger brain connectivity at baseline continued to exhibit stronger brain connectivity than their peers at Year 2. 99.995% of entries were significant after FDR correction with $q < 0.05$. (d) Visualization of FC stability at the regional level, by averaging the rows of panel (c). (e) Longitudinal FC change at the group level based on a linear mixed effects model. 54.96% of entries were significant after FDR correction with $q < 0.05$. (f) Visualization of longitudinal FC change at the regional level, by averaging the rows of panel (e). (g) Individual variability in longitudinal FC change. FC change (z value) was computed for each FC edge [2]. Sex, baseline age, age interval (between baseline and Year 2) and head motion at two timepoints were regressed out. Standard deviation was then computed across individuals. (h) Visualization of individual variability in longitudinal FC change at the regional level, by averaging the rows of panel (g).

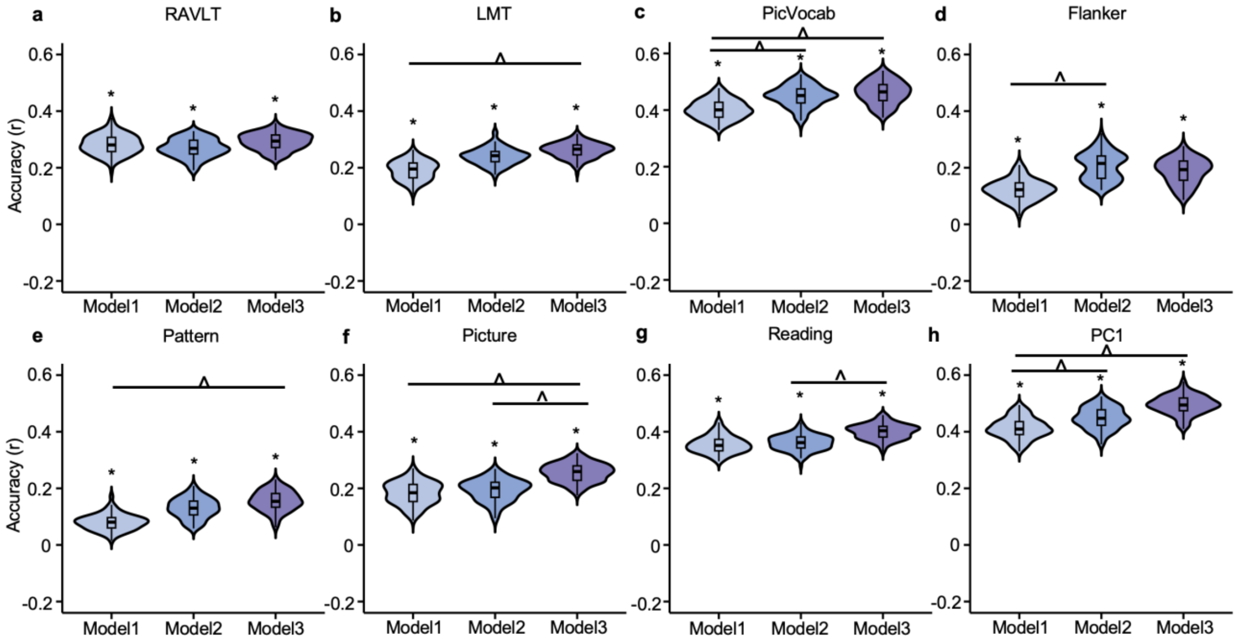

Figure S23. Enhanced FC–cognition relationships during development in a representative subsample (N = 2020). (a-h) Comparison of prediction accuracies across the three models for the eight cognitive measures. Model 1 predicted baseline cognition using baseline FC (FCY0 → CogY0). Model 2 predicted Year 2 cognition using baseline FC (FCY0 → CogY2). Model 3 predicted Year 2 cognition using Year 2 FC (FCY2 → CogY2). Each value in the violin plot represents the accuracy (r) for a single cross-validation fold. Asterisks (*) denote above chance prediction after multiple comparisons correction (FDR $q < 0.05$). Carets (^) denote statistically significant differences between models based on the corrected resampled t-test (FDR $q < 0.05$). RAVLT: Rey Auditory Verbal Learning Test (verbal memory); LMT: Little Man Task (spatial reasoning); PicVocab: Picture Vocabulary Task (vocabulary); Flanker: Flanker Task (executive function); Pattern: Pattern Comparison Processing Speed Test (processing speed); Picture: Picture Sequence Memory Test (episodic memory); Reading: Oral Reading Recognition Task (reading ability). PC1: the first principal component of the above seven cognitive measures.

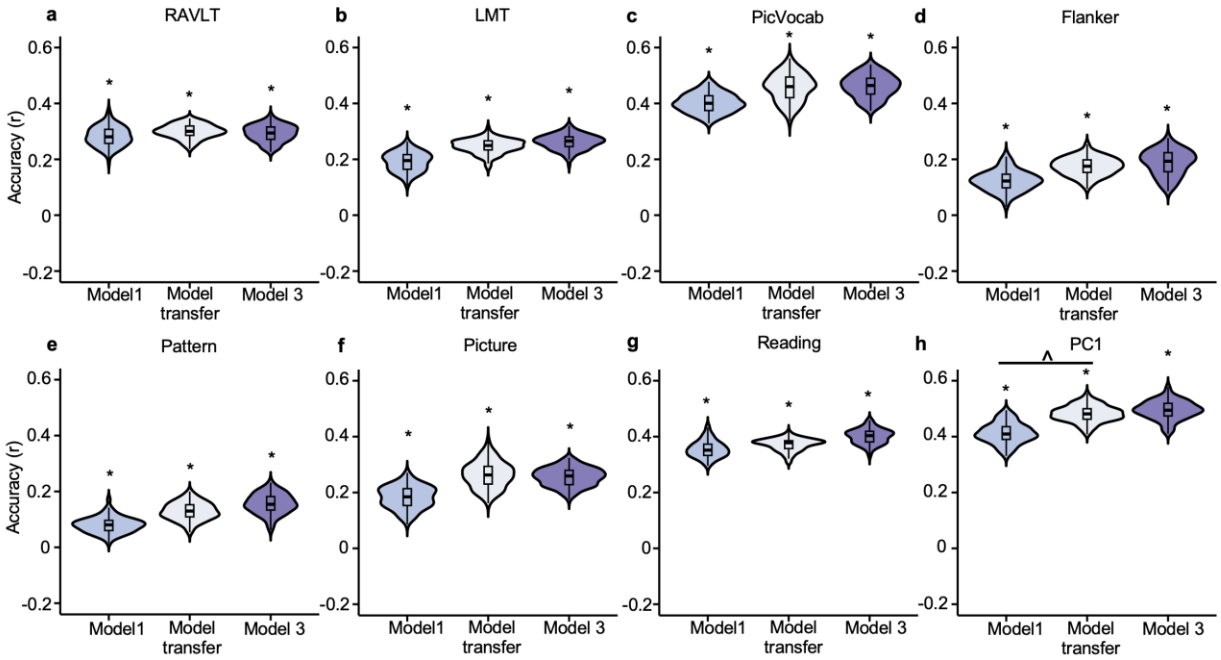

Figure S24. Models trained on baseline FC to predict baseline cognition improve in accuracy when applied to Year 2 FC and Year 2 cognition in a representative subsample (N = 2020). Each plot corresponds to a different cognitive measure. "Model 1" are the models obtained by training baseline FC to predict baseline cognition (FCY0 → CogY0). These models were then used to predict Y2 cognition from Year 2 FC, which we refer to as "Model transfer". Finally, for reference, "Model 3" are the models obtained by training Y2 FC to predict Y2 cognition (FCY2 → CogY2). Asterisks (*) denote above chance prediction after multiple comparisons correction (FDR $q < 0.05$). Carets (^) denote statistically significant differences between models based on the corrected resampled t-test (FDR $q < 0.05$). RAVLT: Rey Auditory Verbal Learning Test (verbal memory); LMT: Little Man Task (spatial reasoning); PicVocab: Picture Vocabulary Task (vocabulary); Flanker: Flanker Task (executive function); Pattern: Pattern Comparison Processing Speed Test (processing speed); Picture: Picture Sequence Memory Test (episodic memory); Reading: Oral Reading Recognition Task (reading ability). PC1: the first principal component of the above seven cognitive measures.

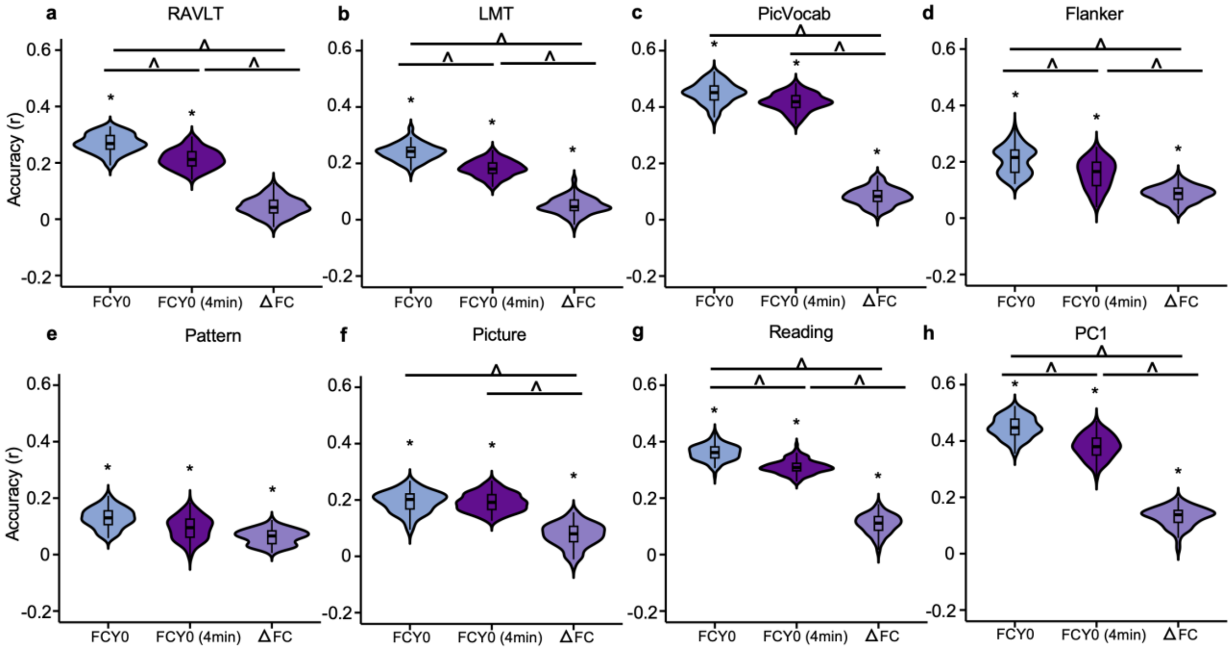

Figure S25. Baseline FC is more predictive of cognition at Year 2 than longitudinal FC change, even accounting for reliability differences in a representative subsample (N = 2020). Each panel corresponds to the prediction accuracy of a different cognitive measure at Year 2. "FCY0" are the models obtained by training baseline FC to predict Year 2 cognition (same as model 2 in Figure 4). "FC (4min)" uses baseline FC computed from the first 4 minutes of fMRI data to predict Year 2 cognition. "ΔFC" are the models obtained by using FC change (between Year 2 and baseline) to predict cognition at Year 2. Asterisks (*) denote above chance prediction after multiple comparisons correction (FDR $q < 0.05$). Carets (^) denote statistically significant differences between models based on the corrected resampled t-test (FDR $q < 0.05$). RAVLT: Rey Auditory Verbal Learning Test (verbal memory); LMT: Little Man Task (spatial reasoning); PicVocab: Picture Vocabulary Task (vocabulary); Flanker: Flanker Task (executive function); Pattern: Pattern Comparison Processing Speed Test (processing speed); Picture: Picture Sequence Memory Test (episodic memory); Reading: Oral Reading Recognition Task (reading ability). PC1: the first principal component of the above seven cognitive measures.

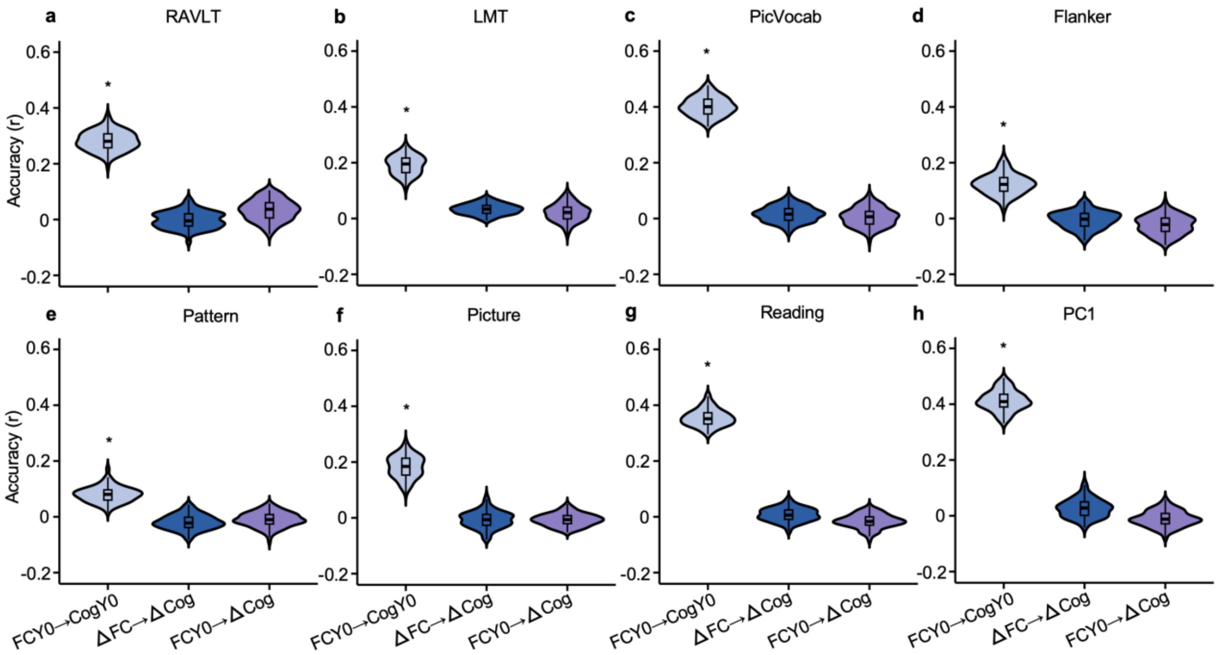

Figure S26. Limited prediction of cognitive change from baseline FC and longitudinal FC change in a representative subsample (N = 2020). Each panel corresponds to the prediction accuracy of a different cognitive measure. Within each panel, baseline FC and longitudinal FC change (ΔFC) were used to predict cognitive change (ΔCog). Predictions of baseline cognition from baseline FC (FCY0 → CogY0) were also shown for reference. Asterisks (*) denote above chance prediction after multiple comparisons correction (FDR $q < 0.05$). RAVLT: Rey Auditory Verbal Learning Test (verbal memory); LMT: Little Man Task (spatial reasoning); PicVocab: Picture Vocabulary Task (vocabulary); Flanker: Flanker Task (executive function); Pattern: Pattern Comparison Processing Speed Test (processing speed); Picture: Picture Sequence Memory Test (episodic memory); Reading: Oral Reading Recognition Task (reading ability). PC1: the first principal component of the above seven cognitive measures.

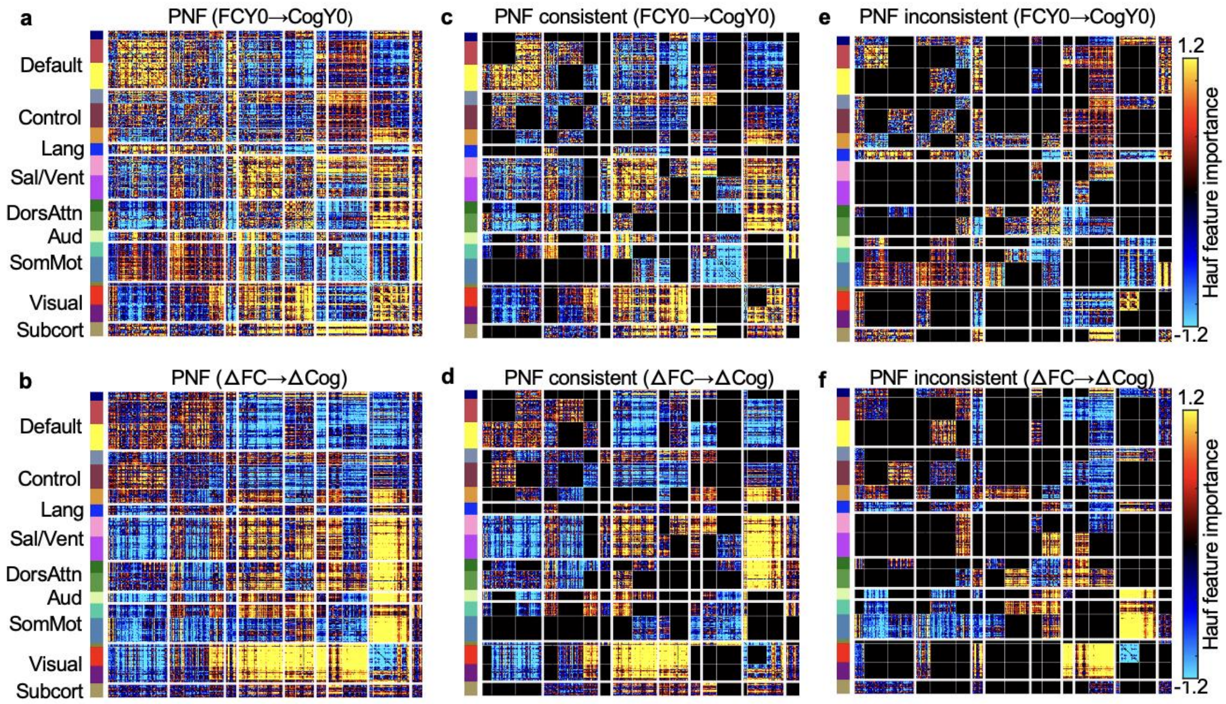

Figure S27. Convergent and divergent predictive network features (PNFs) between cross-sectional and longitudinal estimates of FC–cognition relationship in a representative subsample (N = 2020). (a) PNFs from cross-sectional model using baseline FC (FCY0) to predict baseline Little Man Task (LMT) score (CogY0). (b) PNFs from longitudinal model using changes in FC (ΔFC) to predict changes in LMT performance (ΔCog) across the two timepoints. (c) Network blocks with consistent PNFs in the cross-sectional model. (d) Network blocks with consistent PNFs in the longitudinal model. PNFs were considered consistent if the average feature value for a network block had the same sign in both cross-sectional and longitudinal models. (e) Network blocks with inconsistent PNFs in the cross-sectional model. (f) Network blocks with inconsistent PNFs in the longitudinal model. PNFs were considered inconsistent if the average feature value had opposite signs across models.

R3Q3. The relationships were also only examined across a single 2-year span between 10 and 12 years of age. Whether the conclusion generalizes to either shorter or longer age spans, or to any other age periods is hard to know. This can hardly justify the very general title of "Convergent and Divergent Brain-Cognition Development". Actually, the hypothesis of "convergent and divergent" relationships was only tested in Figure 7 and related results were only represented in a small fraction of the discussion, so the title seems to be a bit off in covering the main findings of the study.

Response: We agree with the reviewer that the title is too general. In the revised manuscript, we have revised the title to be "Convergent and divergent brain–cognition development **in early adolescence**."

Furthermore, we have carefully checked and edited the manuscript to ensure that the limited age range was clearly stated early in the manuscript.

page 2 (Abstract)

Using resting-state functional MRI (rs-fMRI) and cognitive data at baseline and Year 2 of 2,949 individuals in the Adolescent Brain Cognitive Development (ABCD) Study, we examine how individual-level brain–cognition predictive models develop longitudinally in early adolescence.

page 3 (Introduction)

Here, we leverage longitudinal resting-state fMRI and cognitive data from 2,949 children (ages 8.9 to 13.5) in the Adolescent Brain Cognitive Development (ABCD) Study at baseline and Year 2 to examine how stable and changing features of brain network organization predict cognitive development during this critical period.

…

In this study, we examined how FC and cognition co-evolve during early adolescence using longitudinal rs-fMRI and cognitive data collected at baseline and Year 2 from 2949 participants of the ABCD Study[28-30].

Investigation of additional timepoints in the ABCD study will be conducted in future work.

page 23 (Discussion)

Limitations and future work

Our analysis was limited to the first two timepoints. While this restricts our ability to capture nonlinear or individual-specific trajectories of functional connectivity (FC) and cognitive development[84,85], the dataset remains the largest longitudinal sample currently available for investigating brain–cognition relationships during the transition from childhood to early adolescence. Although change estimates derived from only two timepoints may be less precise than those based on more frequent measurements, our findings provide an important early benchmark and a foundation for predictive modeling of developmental processes. Future work leveraging additional timepoints from the ABCD study will be able to explore trajectories that extend further into adolescence.

We also agree with the reviewer that in the original framing of the introduction (and title) focused on “feature-level” convergence and divergence of cross-sectional and longitudinal predictive models, which were only explored in Figure 7. In the revised manuscript, we note that there is now a new Figure 8 exploring sex-specific convergences and divergences between cross-sectional and longitudinal models. Please see our response to R3Q4 for further details.

Furthermore, we have also re-worked the introduction to discuss how “predictive power” might also be convergent or divergent between cross-sectional and longitudinal predictive models. More specifically, baseline connectivity might be predictive of baseline cognition. However, longitudinal change in connectivity may or may not be predictive of future cognitive ability or longitudinal change in cognitive ability. The updated introduction thus foreshadows the results in Figures 4 to 6. We have also expanded Figure 1 to include the new framing.

page 3 (Introduction)

Conceptually, cross-sectional analyses reveal between-individual traits, which might differ from dynamic within-individual changes captured by longitudinal designs[23,24].

Figure 1a illustrates the classical example of how cross-sectional (between-individual) and longitudinal (within-individual) estimates can diverge for a single phenotype, a phenomenon known as the Simpson's paradox[25-27]. When extended to predictive models of brain and cognition, the situation becomes more complex. For example, salience network FC may robustly predict cognitive ability cross-sectionally across individuals (Figure 1b). However, longitudinal change in salience network FC might not be predictive of future cognitive ability or longitudinal change in cognitive ability (Figure 1b).

Beyond predictive power, network features supporting prediction might also differ between cross-sectional and longitudinal models. For example, stronger salience network FC predicted better cognitive ability at the individual level[11]. If this relationship reflects a causal mechanism, one would expect that children with greater increases in salience network FC over time would also show greater cognitive improvement (Figure 1c). However, cross-sectional and longitudinal models may not always align: it is also possible that children with larger reductions in salience network FC exhibit the most cognitive gains (Figure 1d). To put it in another way, suppose Luca is a cognitively advanced child whose brain connectivity patterns differ from his peers. As his peers grow older and become more cognitively capable, do their brains become more like Luca's? If so, this would reflect feature-level convergence between cross-sectional and longitudinal estimates (Figure 1c). If not, this would indicate feature-level divergence (Figure 1d).

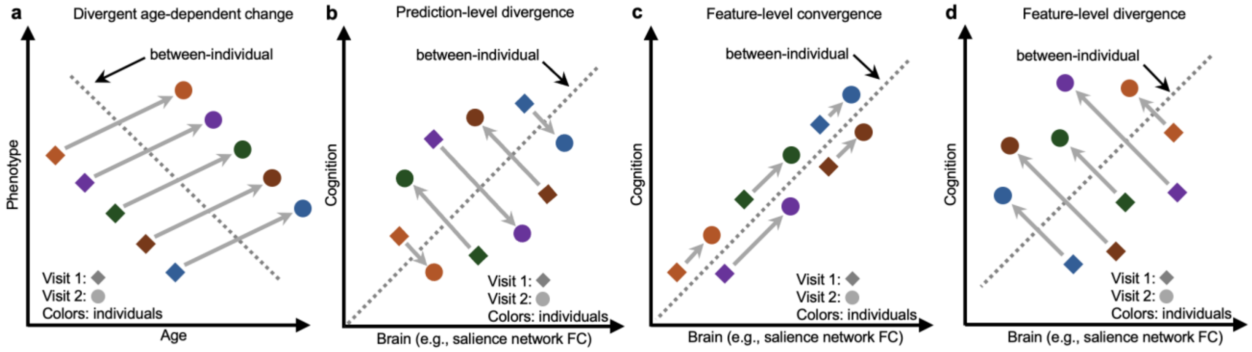

Figure 1. Illustration of how cross-sectional and longitudinal estimates can converge or diverge. (a) Classical (Simpson's paradox) illustration, where within-individual changes (arrows) diverge from between-individual differences (dashed line). (b) Predictive power divergence between cross-sectional and longitudinal predictive models. In this example, salience network FC yields strong cross-sectional prediction of cognitive ability (dotted line). However, longitudinal within-individual change in salience network connectivity is not predictive of cognitive ability at visit 2, as well as longitudinal cognitive change (gray arrows). (c) Feature-level convergence between cross-sectional and longitudinal models. For example, greater salience network connectivity predicts better cognitive ability among children cross-sectionally (dashed line). Assuming a causal relationship, individuals with larger increases in salience network FC should enjoy greater cognitive gains longitudinally (arrows). (d) Feature-level divergence between cross-sectional and longitudinal models. Similar to panel (c), greater salience network connectivity predicts better cognitive ability among children cross-sectionally (dashed line). However, individuals with larger reductions in salience network FC enjoy greater cognitive gains longitudinally (arrows).

Figure S1 illustrates other possible divergences between cross-sectional and longitudinal estimates of brain–cognition relationship. We hypothesize that convergence and divergence vary across brain networks.

R3Q4. Finally, boys and girls are known to develop differently so how about examining these relationships in each sex group?

Response: We thank the reviewer for raising this important point. We have now repeated analyses in males and females separately to investigate sex-specific patterns and to determine whether there were significant sex differences. Below is the summary of our findings:

(1) Sex differences in longitudinal cognitive changes. For both males and females, cognitive abilities at baseline and Year 2 were positively correlated (i.e., cognitively stable), consistent with the result of the full sample combining males and females. There was no sex difference in terms of cognitive stability for all cognitive measures. Longitudinal changes were also similar for males and females, with the exception of the Rey Auditory Verbal Learning Test (RAVLT), where males showed improvement over time, while females exhibited a slight decline. Sex differences in the magnitude of individual variability in cognitive changes were also observed for RAVLT and Pattern Comparison Processing Speed Test.

(2) Sex differences in longitudinal FC changes. The spatial patterns of longitudinal FC stability and longitudinal FC changes were similar in males and females, and consistent with the full sample. Although females exhibited higher FC stability than male across the whole brain, differences were small and only 0.36% of edges survived FDR $q < 0.05$. In the case of longitudinal FC changes, females exhibited lower rate of FC decrease than males in certain brain regions, e.g., anterior insula. There was no statistically significant sex difference in the inter-individual variability in longitudinal FC change.

(3) Sex differences in cross-sectional prediction. Both females and males showed strengthened FC–cognition relationships during development, consistent with the full sample combining males and females. In the case of cross-sectional prediction, there was no sex difference in prediction accuracies across all cognitive measures after multiple comparisons correction.

(4) Sex differences in prediction of Year 2 cognition based on baseline FC or longitudinal FC change. For both males and females, baseline FC was better than longitudinal FC change in predicting Year 2 cognition. The result persisted even when we shorten scan duration of baseline fMRI to 4 mins. Across all predictive models, there was no sex difference in prediction performance.

(5) Sex differences in prediction of longitudinal cognition change. For both males and females, longitudinal change in cognition was poorly predicted by baseline FC, as well as longitudinal FC change. There was no sex difference for all predictive models.

(6) Sex differences in convergent and divergent predictive network features (PNFs) between cross-sectional and longitudinal estimates of brain–cognition relationship.

Consistent with the full sample, there were both convergent and divergent PNFs between cross-sectional and longitudinal models when models were trained separately for each sex. For example, connectivity within the salience network was convergent between cross-sectional and longitudinal models, not only for the full sample, but also separately for males and females. Similarly, connectivity within the visual network was divergent between cross-sectional and longitudinal models, not only for the full sample, but also separately for males and females.

On the other hand, there were also clear sex differences. Predictive network features were substantially more similar between males and females in the cross-sectional models than in the longitudinal models, giving rise to sex-specific convergences and divergences when compared with cross-sectional models.

For changes to the manuscript, please see our response to R2Q3.

Reply to reviewers NCOMMS-25-48986A (Convergent and Divergent Brain–Cognition Development in Early Adolescence)

We thank the reviewers and the editor for their evaluation of our manuscript. All reviewer comments were addressed in the previous revision, and no additional comments were received in the final decision round. Therefore, no further point-by-point response is required.
